# Heterogeneity and convergence across seven neuroimaging modalities: a review of the autism spectrum disorder literature

**DOI:** 10.3389/fpsyt.2024.1474003

**Published:** 2024-10-16

**Authors:** Amanda R. Halliday, Samuel N. Vucic, Brianna Georges, Madison LaRoche, María Alejandra Mendoza Pardo, Liam O. Swiggard, Kaylee McDonald, Michelle Olofsson, Sahit N. Menon, Sunday M. Francis, Lindsay M. Oberman, Tonya White, Isabelle F. van der Velpen

**Affiliations:** ^1^ Section on Social and Cognitive Developmental Neuroscience, National Institute of Mental Health, National Institutes of Health, Bethesda, MD, United States; ^2^ Department of Clinical Neuroscience, Karolinska Institutet, Stockholm, Sweden; ^3^ Noninvasive Neuromodulation Unit, Experimental Pathophysiology Branch, National Institute of Mental Health, National Institutes of Health, Bethesda, MD, United States; ^4^ School of Medicine, University of California, San Diego, San Diego, CA, United States

**Keywords:** autism spectrum disorder, neuroimaging, magnetic resonance imaging, diffusion tensor imaging, magnetic resonance spectroscopy, magnetoencephalography, electroencephalography, near infrared spectroscopy

## Abstract

**Background:**

A growing body of literature classifies autism spectrum disorder (ASD) as a heterogeneous, complex neurodevelopmental disorder that often is identified prior to three years of age. We aim to provide a narrative review of key structural and functional properties that differentiate the neuroimaging profile of autistic youth from their typically developing (TD) peers across different neuroimaging modalities.

**Methods:**

Relevant studies were identified by searching for key terms in PubMed, with the most recent search conducted on September 1, 2023. Original research papers were included if they applied at least one of seven neuroimaging modalities (structural MRI, functional MRI, DTI, MRS, fNIRS, MEG, EEG) to compare autistic children or those with a family history of ASD to TD youth or those without ASD family history; included only participants <18 years; and were published from 2013 to 2023.

**Results:**

In total, 172 papers were considered for qualitative synthesis. When comparing ASD to TD groups, structural MRI-based papers (n = 26) indicated larger subcortical gray matter volume in ASD groups. DTI-based papers (n = 14) reported higher mean and radial diffusivity in ASD participants. Functional MRI-based papers (n = 41) reported a substantial number of between-network functional connectivity findings in both directions. MRS-based papers (n = 19) demonstrated higher metabolite markers of excitatory neurotransmission and lower inhibitory markers in ASD groups. fNIRS-based papers (n = 20) reported lower oxygenated hemoglobin signals in ASD. Converging findings in MEG- (n = 20) and EEG-based (n = 32) papers indicated lower event-related potential and field amplitudes in ASD groups. Findings in the anterior cingulate cortex, insula, prefrontal cortex, amygdala, thalamus, cerebellum, corpus callosum, and default mode network appeared numerous times across modalities and provided opportunities for multimodal qualitative analysis.

**Conclusions:**

Comparing across neuroimaging modalities, we found significant differences between the ASD and TD neuroimaging profile in addition to substantial heterogeneity. Inconsistent results are frequently seen within imaging modalities, comparable study populations and research designs. Still, converging patterns across imaging modalities support various existing theories on ASD.

## 1 Introduction

The prevalence of autism spectrum disorder (ASD) has risen drastically from 6.7 to 27.6 per 1,000 in children in the United States over the past twenty years; thus, investigating its neurobiological underpinnings is critically important (1, 2). ASD is a neurodevelopmental condition characterized by social communication deficits and restrictive interests or repetitive behaviors (3). Its clinical presentation is highly heterogeneous, and different symptom profiles are associated with varying levels of severity and impairment (4, 5). Notably, factors such as age, IQ, sex/gender, race, and ethnicity can greatly affect clinical presentation, others’ perception of the ASD phenotype, and subsequent diagnosis (6–9). As such, it is likely that more than the estimated 1 in 23 male children and 1 in 88 female children in the United States are affected (2, 10). Various factors likely contribute to increasing prevalence of ASD diagnoses, including change of diagnostic criteria, increased awareness of parents and health care providers, and improved recognition of ASD symptoms in girls. Still, these factors do not fully explain the rising incidence of ASD, and care should be taken to not dismiss the notion that the actual number of autistic individuals is increasing, rather than just the number of diagnoses (11, 12).

As the community of individuals affected by ASD continues to grow, it is essential to understand the neurobiological and environmental processes that contribute to autistic people’s highly diverse experiences. While no single neurobiological mechanism or cause has been identified for ASD, numerous genetic and epigenetic processes are implicated in its etiology (13, 14). The genetic heritability of the disorder is well established, with twin studies estimating the proportion of the phenotype variance due to genetic factors to be between 60-90% (15, 16). This should provide a target in the effort to characterize the genetic contribution to the etiology of ASD. However, autistic individuals have substantial genotypic as well as phenotypic variability (17). Developmental processes such as executive function (18, 19) and myelination (20) have heterogeneous trajectories from childhood into young adulthood. Understanding how genetic differences lead to various neurobiological and behavioral presentations will allow clinicians to better understand and support autistic youth.

We aimed to conduct this research through a neurodiversity framework, acknowledging the work that autistic scholars are doing to decrease stigma and increase acceptance in the autism research space (21–23). There is an ongoing discussion in the autism research and advocacy communities about whether person-first language (i.e., “youth with autism”) or identity-first language (i.e., “autistic youth”) should be used when discussing people on the autism spectrum (24, 25). We have chosen to use identity-first (also known as disability-first) language in this review because it currently represents the most accepted language in the autism community (24, 26–28). However, we recognize that all language has the possibility to offend or exclude some individuals, and that language changes over time. Similarly, research priorities in the autism field are changing. Participatory research with ASD stakeholders has highlighted the imbalance between existing research, largely in the biomedical and neuroscience spheres, and the need to investigate issues like stigma and support needs (29–31). Despite the wealth of existing literature on the neurobiology of ASD, it has proven difficult to translate these findings into a causal framework or into real-world change in the lives of autistic people.

Autism research in the 1980s and 1990s focused largely on behavioral phenotyping, genetics, and identifying cognitive features of ASD (32–34). Since the turn of the century, neuroimaging tools have been instrumental in the quest to uncover biological mechanisms underlying the disorder. Structural and functional magnetic resonance imaging (sMRI and fMRI, respectively) techniques, as well as electroencephalography (EEG) and magnetoencephalography (MEG), have steadily advanced and become more accessible to researchers. Novel neuroimaging methods including magnetic resonance spectroscopy (MRS), diffusion tensor imaging (DTI), and functional near-infrared spectroscopy (fNIRS) have been increasingly adopted in ASD research. These technological advancements have led to a large body of literature examining differences between the brain in those with and without ASD. Currently, prominent theories for the etiology of ASD include the excitation/inhibition (35, 36), maternal-immune (37), cerebral connectivity (38), dopamine (39), and mitochondrial dysfunction (40) hypotheses. While neuroimaging research has improved our collective understanding of ASD, the results from these studies have been mixed. For example, there is research supporting increased excitation, increased inhibition, and no excitation/inhibition imbalance in autistic participants (41). These inconsistencies may be due in part to discrepancies in research methods: e.g., many researchers opt to include participants across wide age ranges, making it difficult to identify specific neurodevelopmental patterns. Study populations also differ in the inclusion and consideration of autistic participants with more severe symptoms, greater support needs, intellectual disability, language impairment, and co-occurring disorders. Moreover, hypothesis vs. data-driven approaches in neuroimaging research yield heterogeneous results, and many researchers may be hesitant to report null findings.

Analyses using a single neuroimaging technique may not be sufficient to capture the complex neural correlates underlying such a heterogeneous condition. Comparing similar constructs of brain structure or function (e.g., connectivity) across different neuroimaging modalities (e.g., fMRI, DTI, EEG, and MEG) could provide stronger evidence for specific phenotypic findings. There have been both empirical studies (42–45) and reviews (46–48) describing multimodal investigation into the neural correlates of ASD. However, there have not been any large-scale analyses exploring whether there are consistent brain findings in autistic youth across the vast range of modern neuroimaging modalities. In this literature review, we aim to examine MRI, DTI, MRS, fNIRS, MEG, and EEG findings from the past decade to determine what structural, functional, and chemical differences exist between autistic and neurotypical youth. Moreover, we intend to compare findings across modalities to identify converging patterns and highlight inconsistencies in ASD neuroimaging research.

## 2 Methods

### 2.1 Search strategy

Relevant studies were identified by searching PubMed with the terms detailed in **Supplementary Table 1**. The most recent search was conducted on September 1, 2023. We used the PICOTS framework (population, intervention, comparison, outcome, timing) to formulate search terms. The population included autistic youth across the autism spectrum; the intervention included neuroimaging modalities; the comparison included typically developing youth; the outcome included structural, functional and chemical brain differences between autistic and neurotypical youth; the timing included articles published between January 1, 2013, and September 1, 2023. A separate search was completed for each imaging modality to ensure that a maximum number of papers would be included (**Supplementary Table 1**).

### 2.2 Inclusion and exclusion criteria

Articles were initially included based on the following criteria: empirical research with cross-sectional or longitudinal design; publication in 2013 or later; population with an ASD diagnosis, ASD traits, or family history of ASD and typically developing comparison group; study population mean age under 25 years old; use of at least one neuroimaging modality (computed tomography (CT), DTI, EEG, MEG, fMRI, sMRI, MRS, fNIRS, positron emission tomography (PET), single-photon emission computed tomography (SPECT), ultrasound); article published in peer-reviewed journal; full text available in English.

Articles were excluded based on the following criteria: reviews, meta-analyses, case studies/series, qualitative studies, theses, editorials, clinical trials, and book chapters; unavailable full texts, abstract-only texts, papers published before 2013, papers not published in English; animal research; articles unrelated to autism or imaging, study populations not containing participants with ASD or not containing a TD group; studies of clinical groups with known ASD causes or where the focus is on specific diagnoses (e.g. Fragile X, tuberous sclerosis); does not use a neuroimaging modality listed in inclusion criteria (e.g., electromyography (EMG), postmortem); mean age of study population over 25 years-of-age.

### 2.3 Study selection

**Figure 1:**
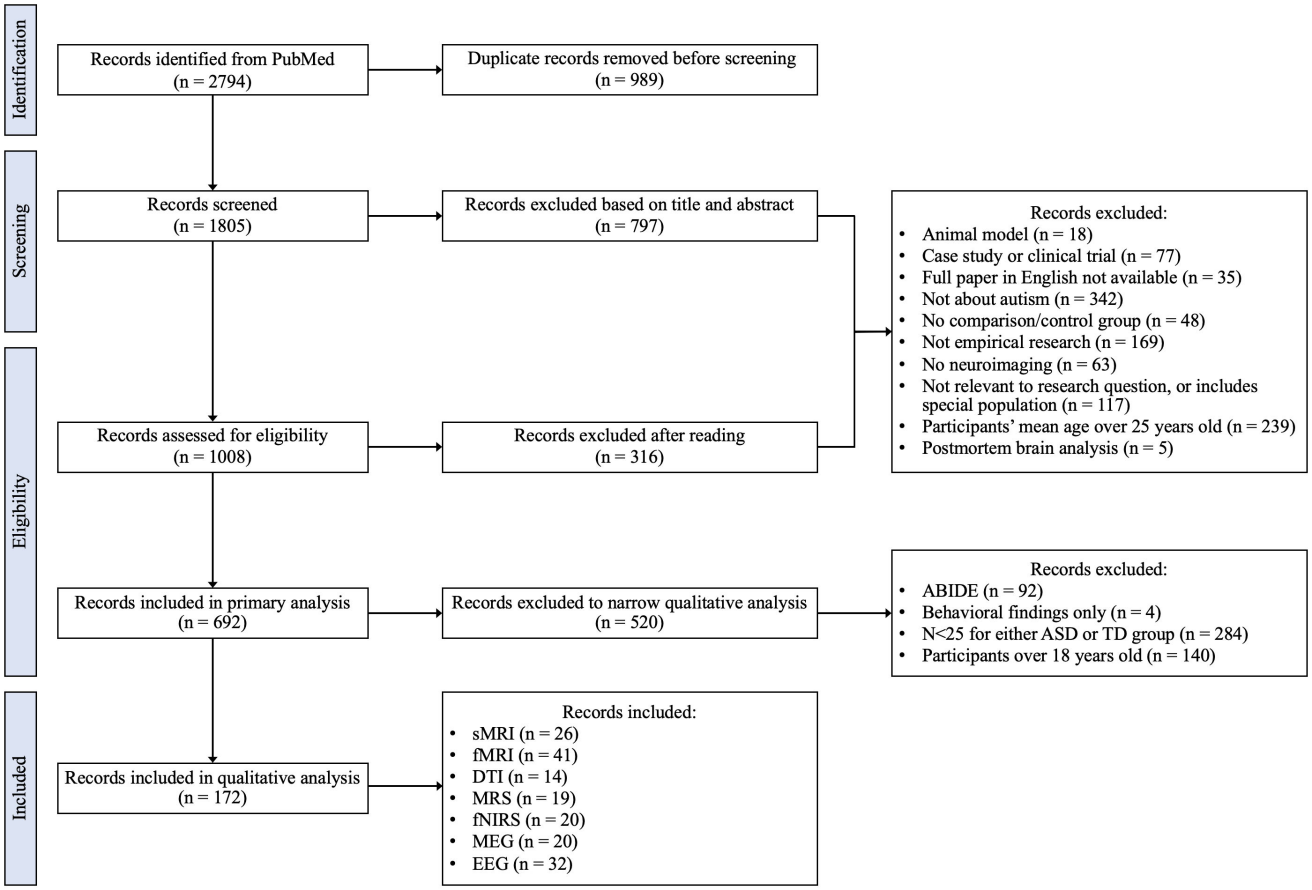
Study selection flowchart.

Study selection involved four separate stages as shown in **Figure 1**. Duplicates were removed prior to title/abstract screening. The work associated with screening, reading, and synthesis was distributed among seven authors (ARH, SNV, BG, ML, MAMP, LOS, MO) at all stages. Team members met weekly to review progress, discuss challenges, and cross-check results and inconsistencies.

Records included in primary evaluation were required to meet all inclusion/exclusion criteria. A breakdown of records included and excluded at each stage in each neuroimaging modality can be found in **Supplementary Table 2**. During primary analyses, the authors reached consensus that additional inclusion/exclusion criteria needed to be implemented to address the heterogeneity of samples and outcomes across papers. Articles that included any participants over the age of 18 were subsequently excluded to focus the literature review on a pediatric population. We based the sample size cutoff on the central limit theorem and only included papers with N > 25 for both the case (ASD) and the control (typically developing youth) groups. This sample size has a greater probability of approximating a normal distribution for the continuous imaging metrics and decreases the risk of including false positives (49, 50). This criterium was not applied to MRS and fNIRS studies, since sample size for these modalities is generally smaller than N = 25, and this exclusion criterium would prohibit any meaningful analysis for these specific modalities. Several articles used data from the Autism Brain Imaging Data Exchange (ABIDE), which resulted in substantial overlap in study populations. Further, not all studies that used ABIDE data specified which ABIDE research sites were included. To limit overlap between study populations in our literature review, only the 2014 ABIDE summary paper was included; all other studies using data from ABIDE were excluded (51). Ultimately, 172 full-text articles were included for the qualitative synthesis.

### 2.4 Data extraction and synthesis

The following data were extracted from each paper: sample size; participants’ age, sex/gender, IQ; ASD measurement tools; study design; country; neuroimaging outcomes comparing ASD and TD groups. Since most articles did not specify how they determined the sex or gender of participants, describe their framework for considering sex differences, and/or did not include female participants, we did not include sex/gender differences in our qualitative analysis (52). We chose to include articles that compared participants with and without a family history of ASD (as opposed to a formal ASD diagnosis) to encompass autistic youth who may be too young to receive a formal diagnosis. Additionally, two papers that did not include participants formally diagnosed with ASD were included to account for the full spectrum of subclinical autism traits. Outcomes specific to behavioral symptoms and within-group comparisons were not included.

We performed a qualitative synthesis to summarize the evidence, since heterogeneous methods, samples, and data reporting across included studies did not allow for quantitative analyses. Key findings from each paper were summarized into **Supplementary Tables 3**–**9**, which were originally organized by modality and ordered based on the average age of the sample. If a paper reported outcomes relevant to two or more modalities, those outcomes were listed under each relevant modality. Team members discussed key findings and brain regions that emerged as relevant across multiple modalities. One team member (SNV) summarized key findings for relevant brain regions: findings relevant to the regions that were most frequently identified in each of **Supplementary Tables 3**–**9** were included. These findings were organized by region, then modality, and data was extracted for total sample size (ASD and TD groups), mean age (weighted mean between ASD and TD groups), and neuroimaging outcome.

Key outcomes for MEG and EEG papers followed a different framework since encephalography does not provide adequate spatial specificity for region-specific evaluation. MEG and EEG findings were organized into tables based on domain (e.g. event-related potentials, frequency/power). Team members then discussed concordant findings between MEG and EEG studies, as well as commonalities between these studies and those using MR-based neuroimaging methods.

To safeguard consistency during qualitative synthesis, two separate team members read each paper. The primary reader synthesized key findings of the paper. The second reader cross-validated the synthesized outcomes with the original findings reported in the paper. In case of disagreement between the primary and a second reader, a third reader (IFV) reviewed the paper and determined the final outcome.

Since we did not perform a formal systematic or scoping review, we did not perform a formal quality and bias assessment of each paper. However, we identified areas of potential and major limitations during reading and consensus meetings, and we will discuss these limitations in Section 4.1 below.

### 2.5 Figures

Box and violin plots were created using R Statistical Software (v4.4.0, R Core Team 2024) with the following packages: magrittr v2.0.3 (53), tibble v3.2.1 (54), vctrs v0.6.5 (55), ggplot2 v3.5.1 (56), tidyr v1.3.1 (57), and plyr v1.8.9 (58). We summarized all findings from 172 papers into three-dimensional brain figures and bubble plots. To visualize recurring themes in neuroimaging literature by modality, bubble plots were generated to identify mentions of specific brain regions and metrics/domains measured across studies. Findings were organized by neuroimaging modality. Each finding was registered as an entry including sample size, brain region, and the associated metric/domain. Findings were manually assigned directional values (-1, -0.5, 0, 0.5, and 1) indicating negative, partial negative, neutral, partial positive, or positive directionality relative to participants with ASD. For example, language indicating ‘ASD group has more’, ‘ASD group has higher’, or ‘ASD group has faster’ corresponded to positive values, while terms like ‘less’, ‘lower’, and ‘slower’ were assigned negative values. Findings without group differences were assigned values of 0. Partial directionality was assigned to findings involving sex-related or ASD-subtype group differences. Additionally, findings were weighted by sample size. Eight bubble plots were generated to depict the aggregated directionality and sample size weights for findings categorized by brain region and related metric/domain. The “whole brain” row in each bubble plot represents outcomes in papers that performed whole-brain analysis for a given metric, not the sum of our own analyses for that metric. We have chosen not to display numeric values for aggregated directionality because of the qualitative nature of this review, and the numeric values do not reflect statistical significance. Future studies that combine the raw data from each modality and each study and perform meta-analyses would be well suited to provide quantitative findings. Bubble plots were created in RStudio, using packages forcats v1.0.0 (59) and ggplot2 v3.5.1 (56).

To create the three-dimensional brain figures, we documented the directionality of differences between ASD and TD groups findings per imaging modality, associated brain regions, and sample size. Next, we created two values for every brain region found in each modality: directionality and opacity. Cumulative directionality for each brain region was calculated by combining all papers that found a significant difference or null finding between ASD and TD group: a positive direction indicated that ASD group had a higher value of a given neuroimaging metric compared to TD groups; a negative direction indicated that the ASD group had a lower value of a given neuroimaging metric compared to TD groups; neutral indicated a null finding. For example: several papers with conflicting findings on regional volume may result in a cumulative null as directionality score. Opacity ranges from 0 to 1 and is calculated by summing the sample sizes of all papers contributing to a region’s directionality score and dividing this number by the largest combined sample size found in that region. Thus, the brain region which has the largest overall sample size will have an opacity of 1, being fully visible, while regions that do not have any findings will have a sample size of 0, thus being invisible in the figure. We then applied these directionality and opacity scores to the brain regions included in the Desikan-Killiany atlas (60). All brain figures were created in ITK-SNAP Version 4.2.0. The directionality score dictates the red/green/blue (RGB) value of the region, ranging from blue (negative) to red (positive) (61). In summary, the color is determined by the directionality and the opacity by sample size. For DTI, we applied the same process but used the ITT Human Brain Atlas (v.5.0) WM atlas. In this atlas, every white matter voxel is assigned a label indicating the two most likely gray matter regions that are connected by the white matter in the voxel (62, 63). For fMRI, we included a functional connectivity figure, which was visualized using the BrainNet Viewer Version 1.7 (64). We presented functional connectivity via a node network. The nodes represented brain regions (i.e. amygdala, anterior cingulate cortex, posterior cingulate cortex, prefrontal cortex, dorsolateral prefrontal cortex, temporal lobe central gyri, occipital lobe, parietal lobe, thalamus, cerebellum, and frontal lobe). The edges represented the functional connectivity findings between these regions.

## 3 Results

**Figure 2:**
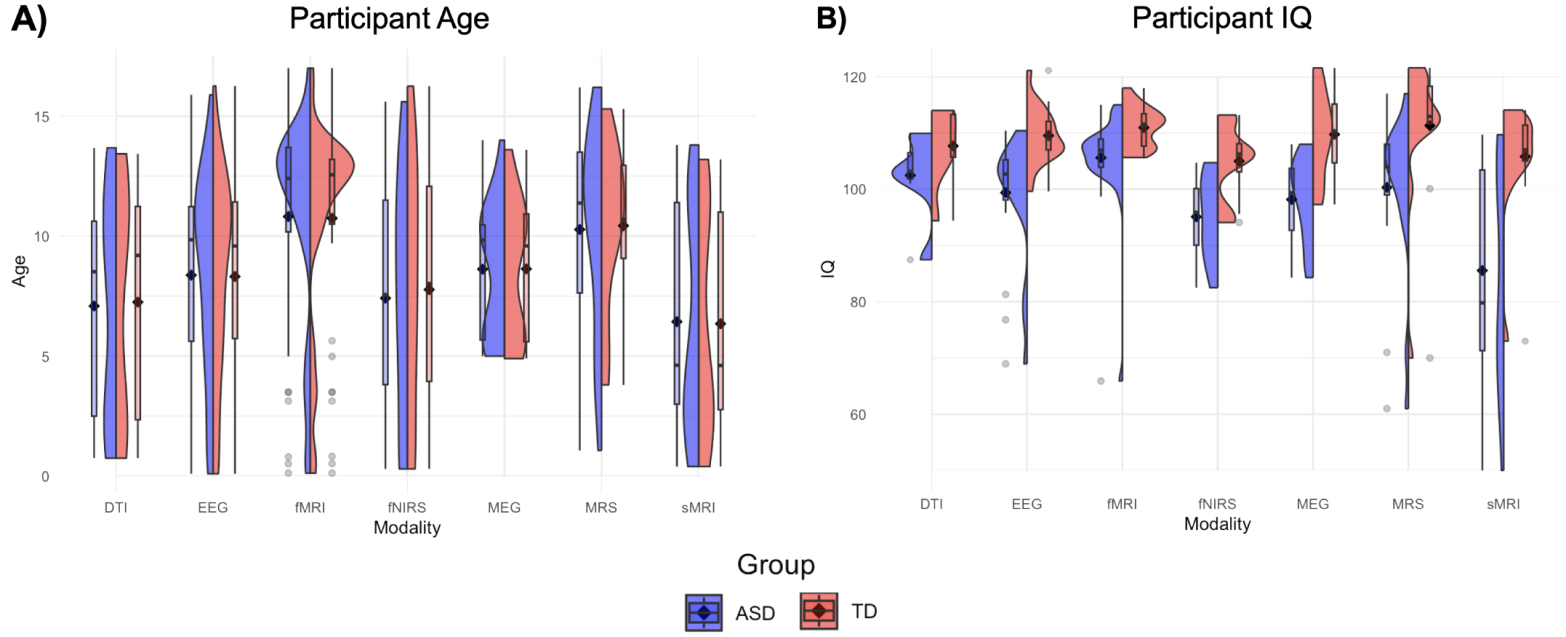
Age and IQ violin plots. **(A)** Box plots depict mean age (black diamond) and median age (black dash) for ASD (left, blue) and TD (right, red) participants across all publications within a modality. Violin plots convey distribution of mean ages across studies within a neuroimaging modality. **(B)** Box and violin plots depict mean, median, and distribution of mean IQ for studies within each neuroimaging modality.

**Figure 3:**
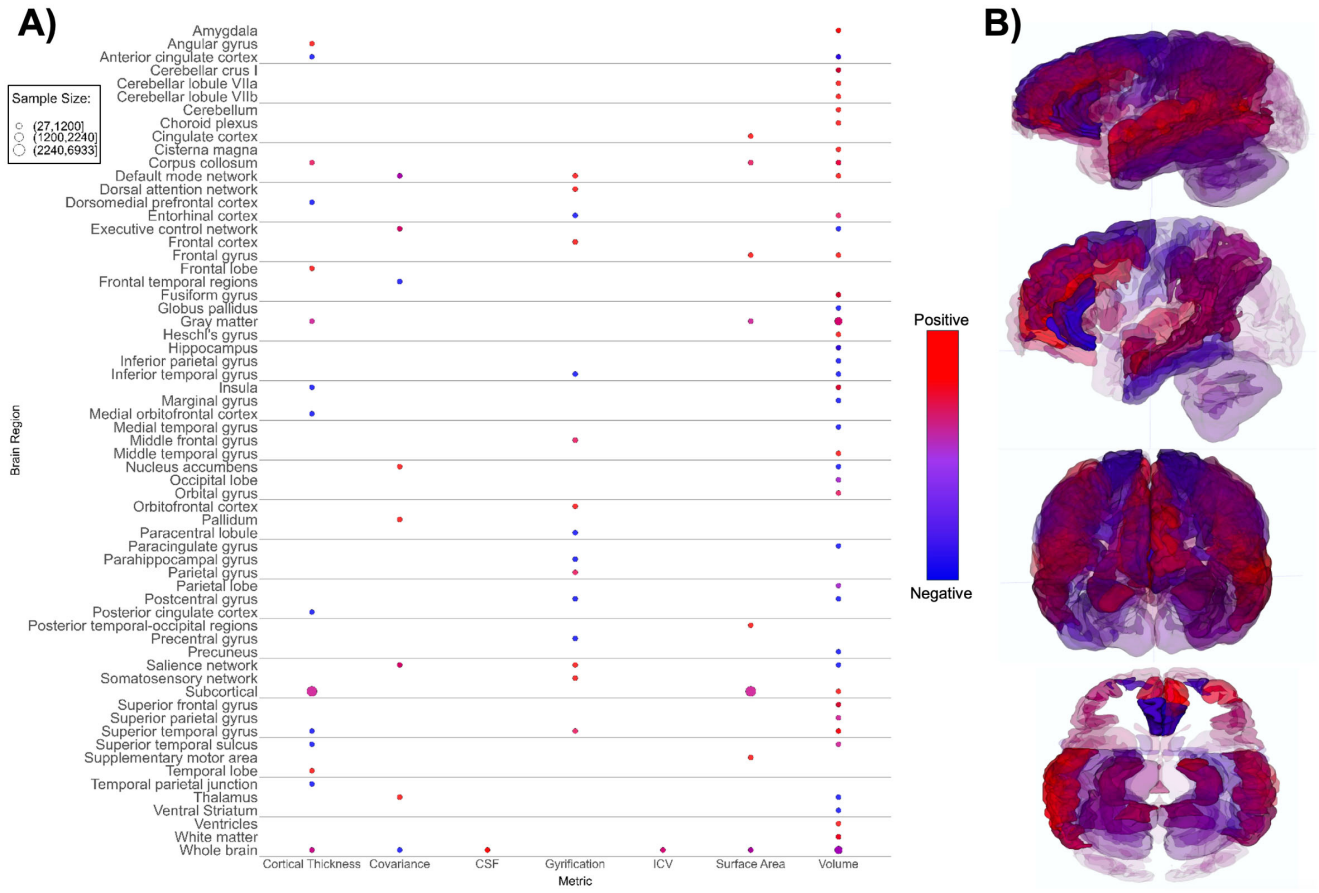
Patterns of sMRI findings. **(A)** Bubble plot and **(B)** brain map depicting regional patterns of structural MRI findings. Red indicates positive findings (e.g. larger volume), while blue indicates negative findings (e.g. smaller volume). Bubble size in **(A)** and opacity in **(B)** represent the number of participants associated with each finding.

**Figure 4:**
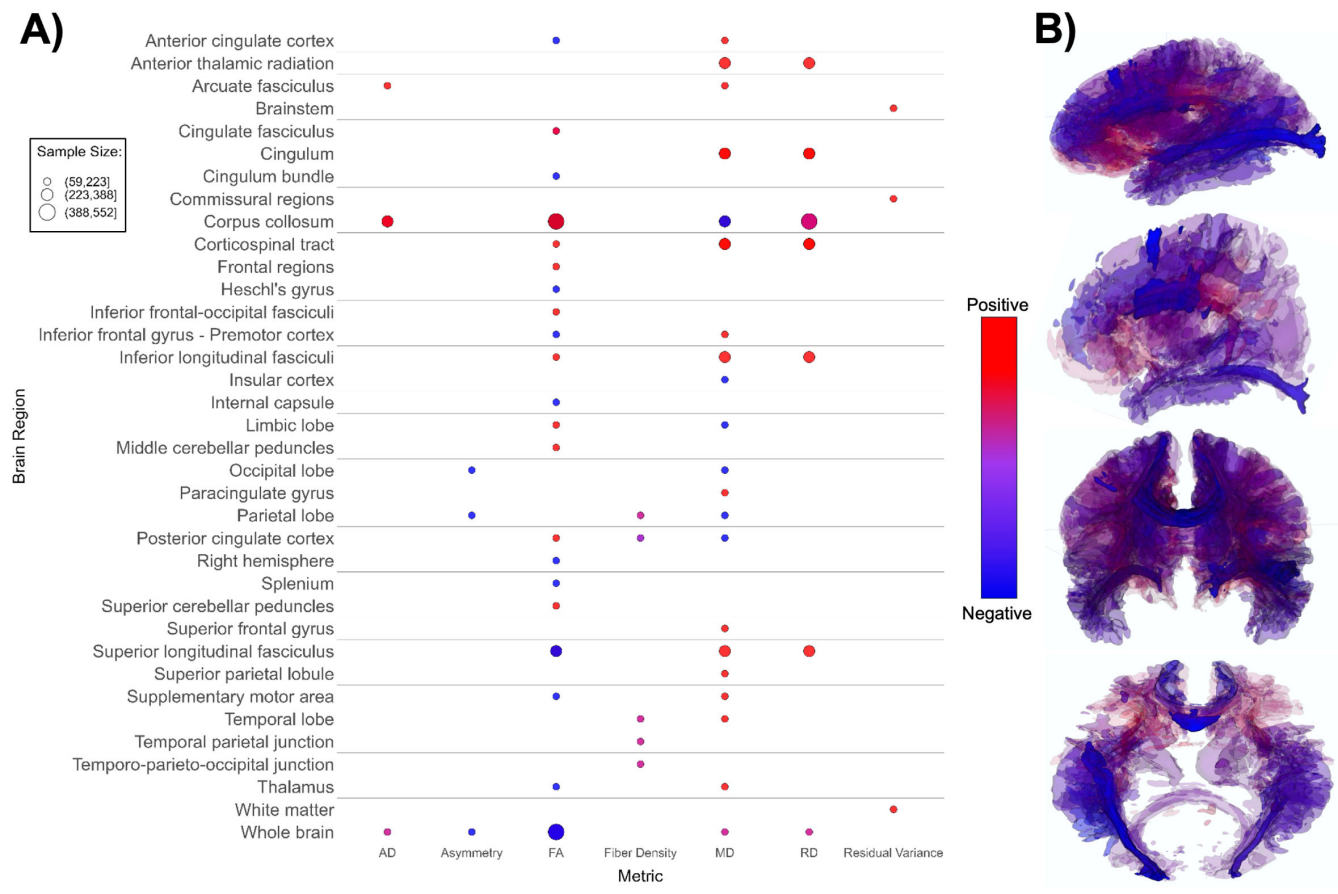
Patterns of DTI findings. **(A)** Bubble plot and **(B)** brain map depicting regional patterns of DTI findings. Red indicates positive findings (e.g. higher diffusivity), while blue indicates negative findings (e.g. lower fractional diffusivity). Diffusivity directionality signage was inversed for brain maps to reflect white matter integrity (i.e. higher mean diffusivity values and lower fractional anisotropy values are both mapped as blue). Bubble size in **(A)** and opacity in **(B)** represent the number of participants associated with each finding.

**Figure 5:**
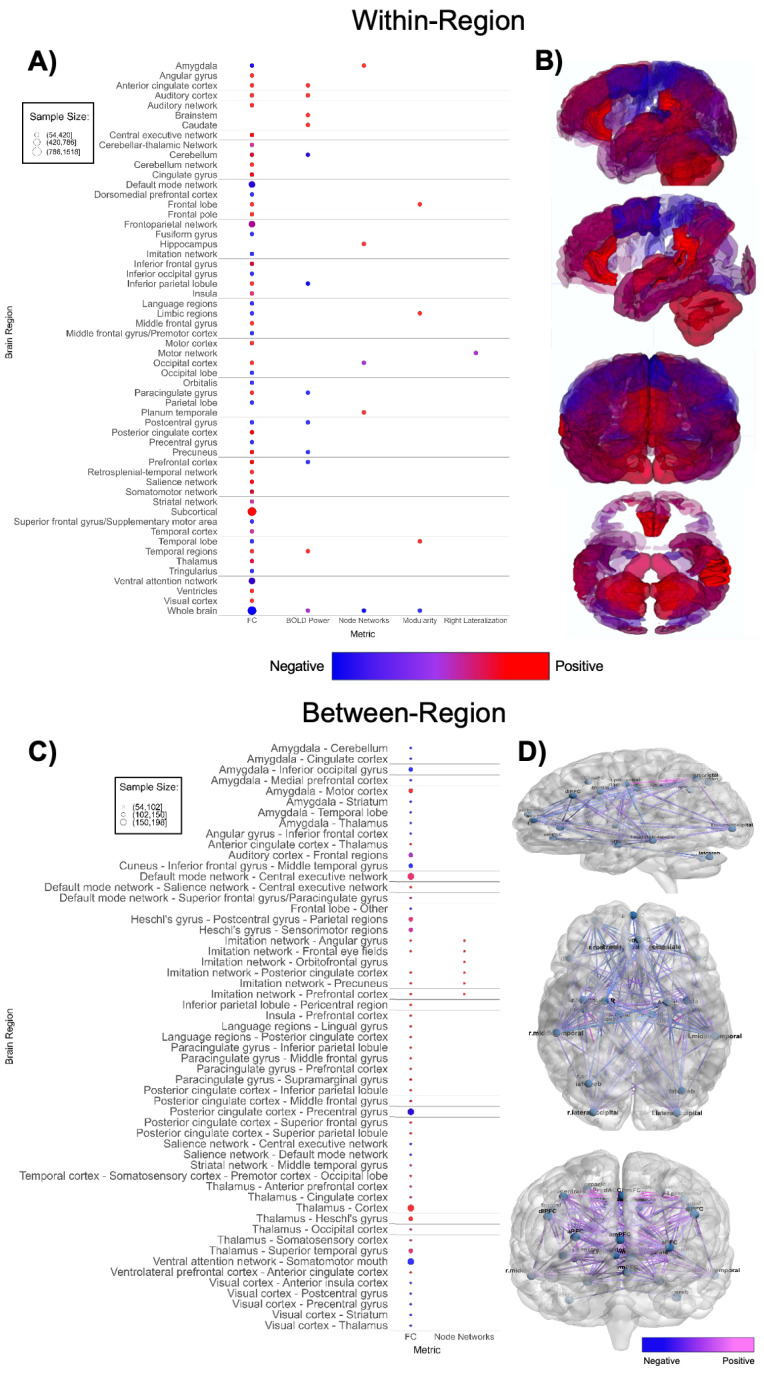
Patterns of fMRI findings. **(A)** Bubble plot and **(B)** brain map depicting intra-regional patterns of functional MRI findings. Red indicates positive findings (e.g. increased local functional connectivity), while blue indicates negative findings (e.g. decreased local functional connectivity). **(C)** Bubble plot and **(D)** brain map depicting inter-regional patterns of functional MRI findings. Pink indicates positive findings (e.g. increased long-range functional connectivity), while blue indicates negative findings (e.g. decreased long-range functional connectivity). Bubble size in **(A, C)** and opacity in **(B, D)** represent the number of participants associated with each finding.

**Figure 6:**
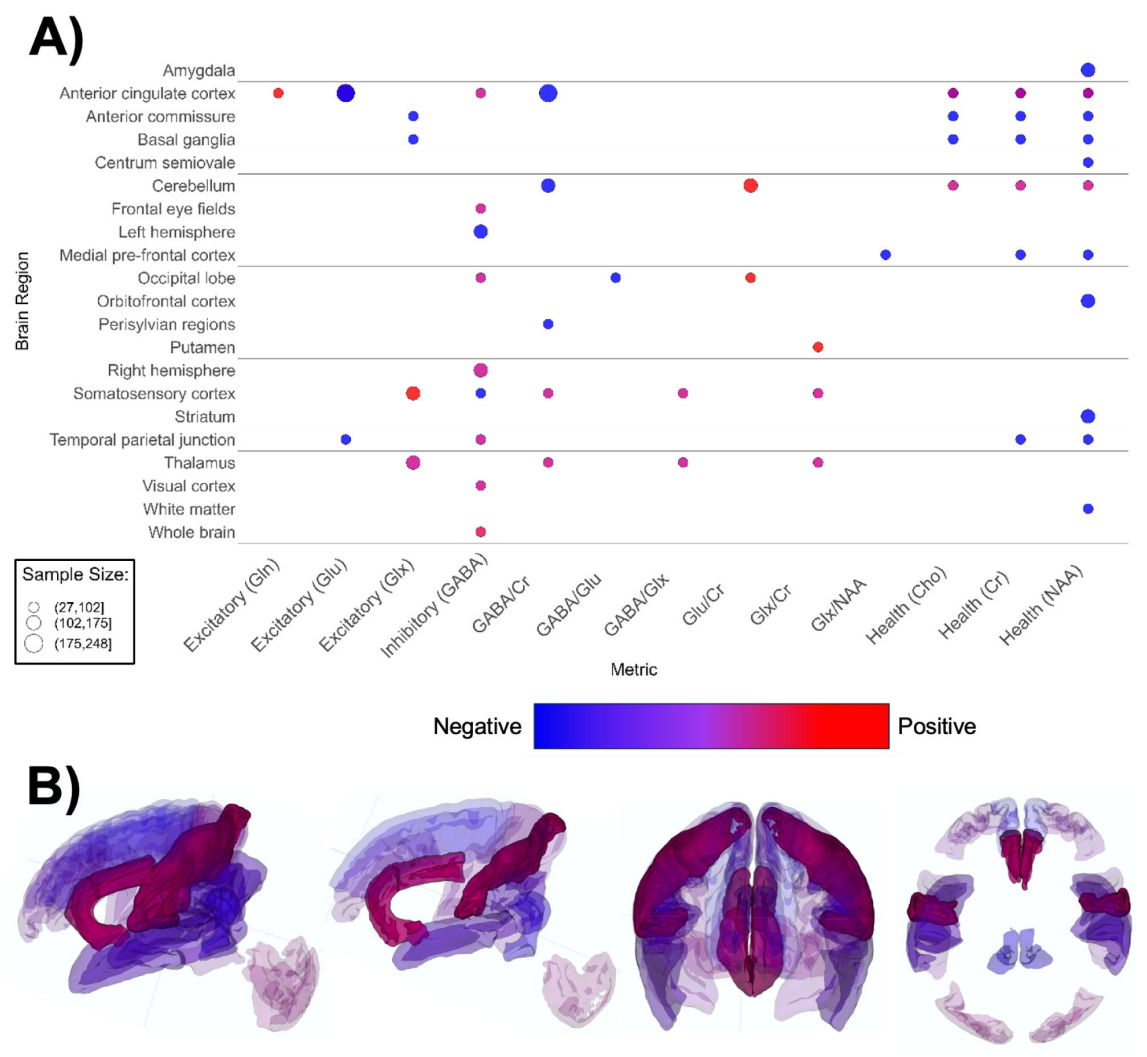
Patterns of MRS findings. **(A)** Bubble plot and **(B)** brain map depicting regional patterns of MRS findings. Red indicates positive findings (e.g. increased metabolites associated with excitation), while blue indicates negative findings (e.g. increased metabolites associated with inhibition). Bubble size in **(A)** and opacity in **(B)** represent the number of participants associated with each finding.

**Figure 7:**
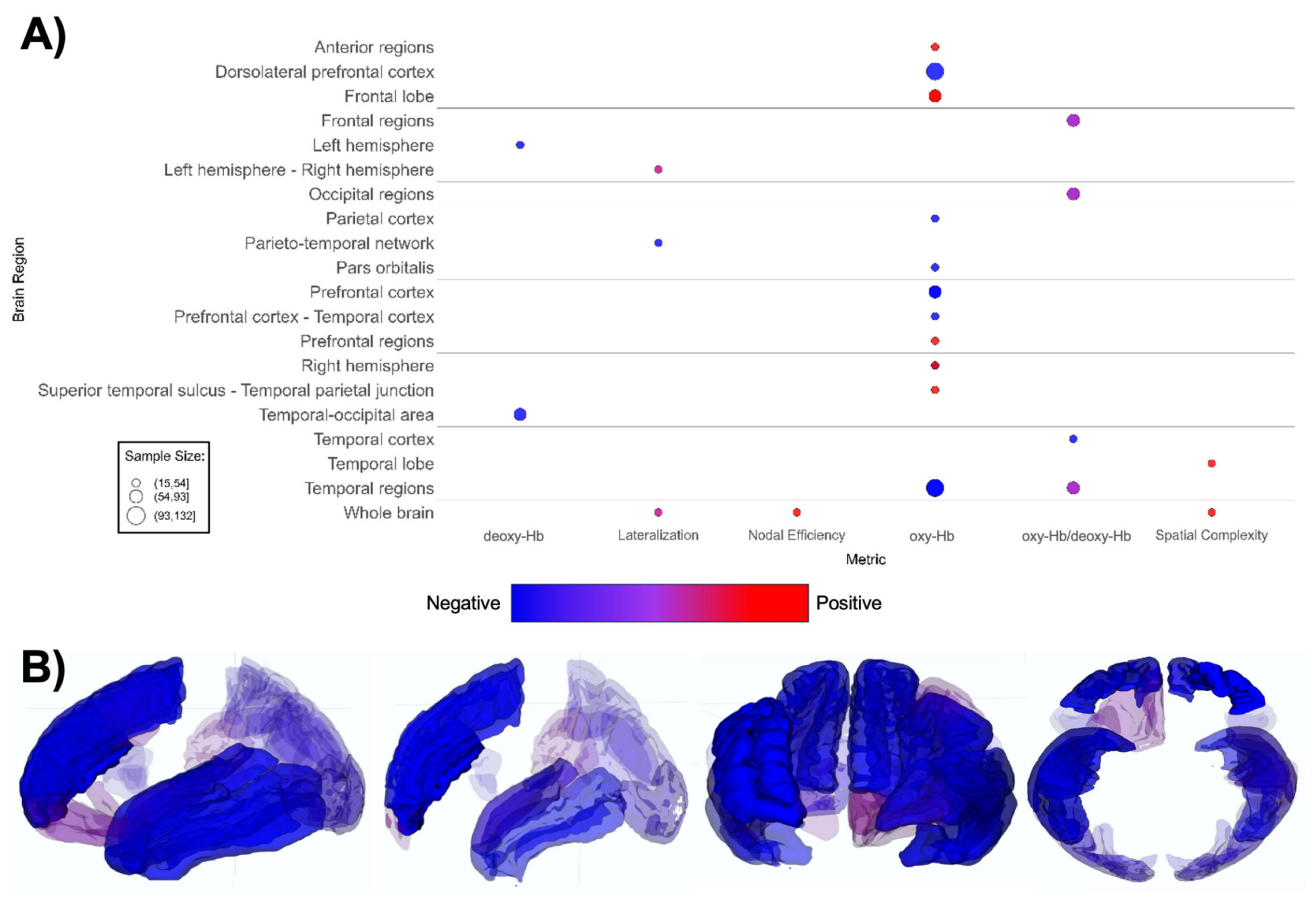
Patterns of fNIRS findings. **(A)** Bubble plot and **(B)** brain map depicting regional patterns of fNIRS findings. Red indicates positive findings (e.g. increased oxy-Hb), while blue indicates negative findings (e.g. increased deoxy-Hb). Bubble size in **(A)** and opacity in **(B)** represent the number of participants associated with each finding.

**Figure 8:**
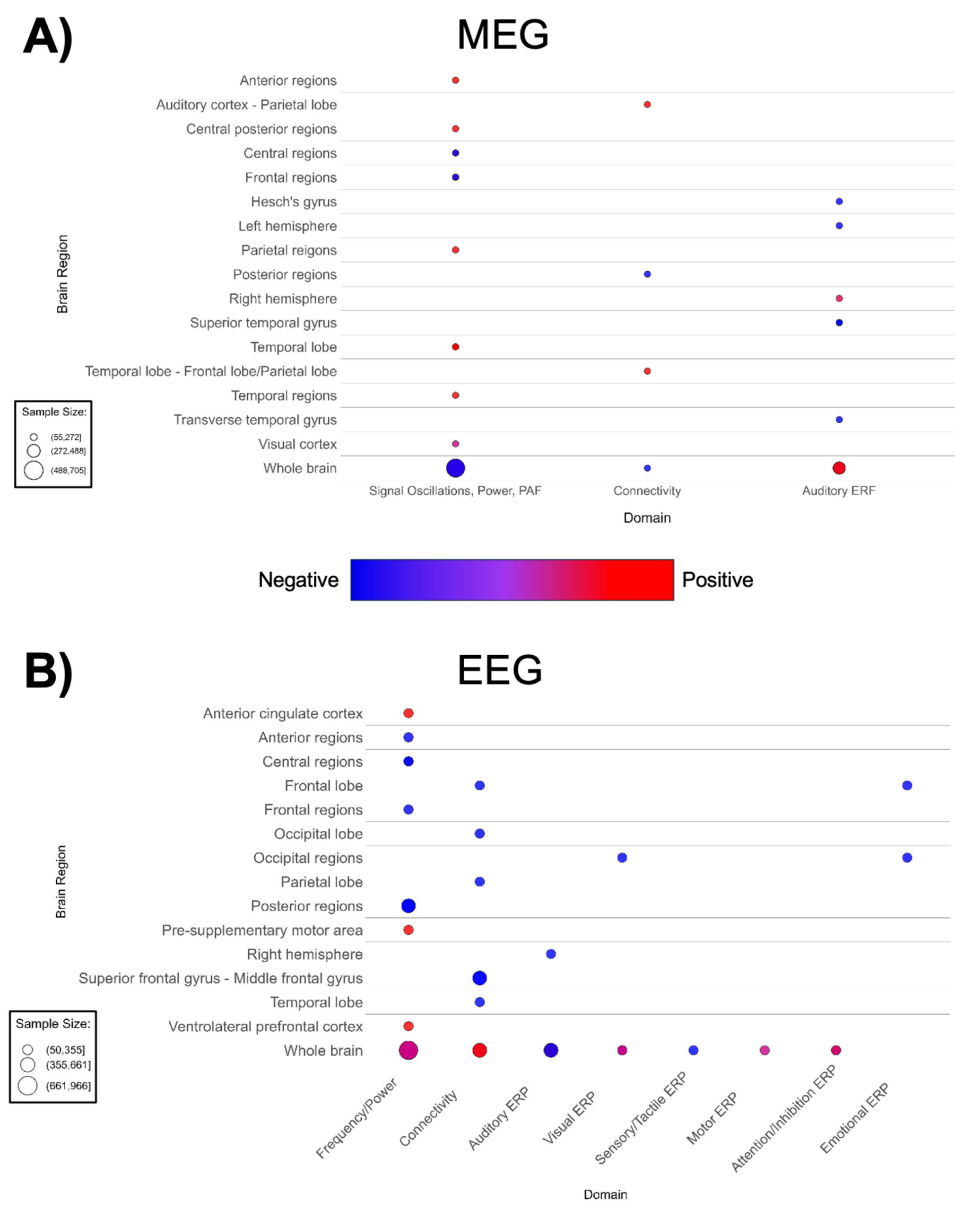
Patterns of MEG and EEG findings. **(A)** Bubble plots depicting regional patterns of **(A)** MEG and **(B)** EEG findings. Red indicates positive findings (e.g. increased total power), while blue indicates negative findings (e.g. decreased total power). Bubble size in represents the number of participants associated with each finding.

Results are presented in the following order: study selection process (**Figure 1**), characteristics of selected papers (**Table 1**, **Figure 2**, Section 3.1), within-modality findings in Section 3.2, including global and brain map/bubble plot-based findings, followed by region-specific findings across modalities in Section 3.3. Global findings reference **Supplementary Tables 3**–**9**, brain maps and bubble plot-based findings reference **Figures 3**–**8**, and region-specific findings reference **Tables 2**–**4**. An additional brain map depicting subcortical regions relevant in each modality is available in **Supplementary Figure 1**.

**Table 1:** Study characteristics.

Characteristic	N (% Total N)
**Total Records**	172
Age	
0-5 years old	55 (32)
6-10 years old	48 (28)
11-15 years old	66 (38)
15-18 years old	3 (2)
Sex/Gender	
Females Included	141 (82)
All Male	28 (16)
Sex/gender Not Reported	3 (2)
IQ	
IQ Not Reported	53 (31)
IQ Matched Groups	38 (22)
ASD Avg. IQ Lower	35 (20)
No Group Difference	25 (15)
Threshold 75	13 (7)
Threshold 80	8 (5)
Sample Size	
N<50 (MRS, MEG, fNIRS)	28 (16)
51-100	77 (45)
101-200	49 (28)
N>200	18 (11)
Design	
Cross-sectional	152 (88)
Longitudinal	20 (12)
Population Type	
Case/Control	157 (91)
Family history/No family history	13 (8)
TD Only	2 (1)
Region	
North America	100 (58)
Asia	43 (25)
Europe	20 (12)
Europe, North America	4 (2)
Asia, Europe	1 (0.6)
Asia, North America	1 (0.6)
Australia	1 (0.6)
Europe, Middle East	1 (0.6)
Middle East	1 (0.6)
Modality	
fMRI	41 (24)
EEG	32 (18)
sMRI	26 (15)
fNIRS	20 (12)
MEG	20 (12)
MRS	19 (11)
DTI	14 (8)

**Table 2:** Regional findings: cortical gray matter.

Region	Modality	Total N	Mean age (y)	Finding (ASD vs. TD)	Ref.
**Anterior Cingulate Cortex (ACC)**	**sMRI**	180	13.5	ASD group has a 3% decrease in cortical volume, partially driven by ACC	(75)
		414	3.2	ASD males show one of the greatest regional volumetric differences compared to TD males around 3 years of age in ACC	(73)
	**DTI**	142	13.4	Increased MD and decreased FA in the tract connecting thalamus to right ACC	(133)
	**fMRI**	111	12.9	Increased regional homogeneity in bilateral ACC at rest	(143)
		54	15.7	Increased functional connectivity between ACC and ventrolateral prefrontal cortex during the Preparing to Overcome Potency Task	(136)
	**MRS**	53	5.3	Children with Asperger’s have decreased NAA, Cr, Cho and mI	(152)
		177	6.5	Increased Glu	(156)
		133	10.6	Increased Glu	(159)
		14	14.0	Increased Glu	(157)
		27	14.8	Increased Gln	(146)
		74	11.0	Glu decreases more between roughly ages 11-13 (longitudinal)	(155)
	**EEG**	50	10.8	Reduced increase of task-related theta current density in 600 to 900 ms window during the Wisconsin Card Sorting Task	(234)
**Insula (INS)**	**sMRI**	78	2.5	Increased white matter volume in ASD toddlers in right INS (longitudinal)	(102)
		191	11.6	Decreased cortical thickness in right INS	(66)
	**DTI**	78	13.4	Decreased MD in insular cortex	(102)
	**fMRI**	65	12.0	Increased functional connectivity between left INS and left prefrontal cortex when viewing neutral faces	(107)
		56	12.0	Increased functional connectivity between left INS and left prefrontal cortex when viewing neutral faces	(106)
		61	12.9	Increased connectivity between anterior INS and sensorimotor areas during passive auditory and tactile stimulation	(118)
		93	13.6	Decreased connectivity between anterior INS and visual cortices at rest	(121)
**Prefrontal Cortex (PFC)**	**sMRI**	146	12.5	Significantly less gray matter in left anterior PFC	(78)
	**fMRI**	72	3.5	Weaker functional connectivity between medial PFC and amygdala during sleep	(134)
		63	12.0	Decreased activation in dorsomedial PFC during executive function tasks	(125)
		65	12.0	Increased functional connectivity between left PFC and left insula when viewing neutral faces	(107)
		95	12.6	Posterior cingulate cortex-medial PFC connectivity at rest decreases with age in ASD group but increases with age in TD group	(111)
		57	12.8	Weaker positive linear relationships between functional activation and cognitive load in bilateral PFC during Letter Matching Task	(138)
		70	13.5	Significant overconnectivity between frontopolar PFC and nodes within the imitation network at rest	(113)
		80	13.0	Increased functional connectivity between paracingulate gyrus and inferior PFC at rest	(108)
		58	13.7	Strong local connectivity in medial PFC at rest	(123)
		79	13.7	Increased functional activation in PFC during mild aversive auditory and tactile stimulation	(122)
		70	13.8	Higher thalamic GABA/Cr is correlated more strongly with lower functional connectivity between the thalamus and anterior PFC at rest (fMRI combined with MRS)	(163)
		54	15.7	Between early and late adolescence, the ASD group shows an increase in functional connectivity between ventrolateral PFC and anterior cingulate cortex during the Preparing to Overcome Potency Task, while TD shows a decrease	(136)
	**MRS**	34	13.0	Decreased NAA, Cr, Glx/NAA in the medial PFC	(145)
	**fNIRS**	98	5.0	Significantly lower oxy-Hb in right dorsolateral PFC in children when “socially interacting” with video clips of a robot	(167)
		32	11.5	Lower oxy-Hb signal during a theory of mind task	(168)
		32	12.7	Lower peak relative oxy-Hb signal in response to a noxious cold stimulus	(177)
	**EEG**	50	10.8	Significantly decreased task-related theta current density in 600 to 900 ms time window in left ventrolateral PFC during Wisconsin Card Sorting Task	(234)

Cho, choline-containing compounds; Cr, creatine/phosphocreatine; FA, functional anisotropy; GABA/Cr, gamma-aminobutyric acid to creatine ratio; Glu, glutamate; Gln, glutamine; Glx/NAA, combined glutamate/glutamine signal to n-acetylaspartate ratio; MD, medial diffusivity; mI, myo-inositol; NAA, n-acetylaspartate.

**Table 3:** Regional findings: subcortical gray matter.

Region	Modality	Total N	Mean age (y)	Finding (ASD vs. TD)	Ref.
**Amygdala (AMYG)**	**sMRI**	126	1.9	Increased volume in right hemisphere	(86)
		420	3.0	Increased volume in right hemisphere	(79)
		408	0.5	AMYG is of a typical volume at 6 months, but disproportionately large in volume at 12 months, radical overgrowth between 6 and 24 months (longitudinal)	(85)
		414	3.2	Increased gray matter network structure in areas of monosynaptic connection	(73)
	**fMRI**	72	3.5	Weaker functional connectivity with the medial prefrontal cortex, striatum, thalamus, cingulate cortex, and cerebellum during sleep	(134)
		106	10.4	Decreased degree centrality during rest	(110)
		142	13.4	Decreased functional connectivity within AMYG during rest	(133)
		105	13.6	Decreased functional connectivity with inferior occipital gyrus, increased functional connectivity with primary motor and somatosensory cortex during rest	(114)
	**MRS**	34	13.0	Decreased NAA	(158)
**Thalamus (THL)**	**sMRI**	78	5.4	Decreased inter-hemispheric covariation and increased intra-hemispheric structural covariation	(68)
		210	4	Smaller regional volume	(87)
		121	12.5	ASD group has no positive correlation between THL volume and age, TD group has both	(189)
	**DTI**	142	13.4	Increased MD, decreased FA	(133)
	**fMRI**	72	3.5	Weaker functional connectivity with amygdala, weaker functional connectivity with primary visual cortex during sleep	(134)
		117	12.4	Higher functional connectivity within THL during rest	(127)
		198	12.8	Widespread overconnectivity with cortex during rest	(225)
		70	13.8	Higher thalamic GABA/Cr is correlated more strongly with lower functional connectivity between the THL and the somatosensory cortex, occipital cortex, anterior prefrontal cortex, and cerebellum during rest (fMRI combined with MRS)	(163)
		78	13.8	Increased connectivity between subregions of the auditory cortex and THL during rest	(129)
		125	0.125	9-month-old high-risk infants relative to low-risk infants have increased connectivity between THL and right superior temporal gyrus during sleep (longitudinal)	(130)
		116	9.7	Increased functional connectivity between THL and Heschl’s gyrus during sleep (longitudinal)	(128)
**Cerebellum (CB)**	**sMRI**	210	4	Lobule 3 of CB shows lower relative volume until 11 years of age, followed by a larger relative volume in adolescence	(87)
	**fMRI**	72	3.5	Weaker functional connectivity with amygdala during sleep	(134)
		114	11.7	Increased task-related functional activation during rest	(132)
		111	12.9	Increased regional homogeneity in right posterior CB, decreased regional homogeneity in right anterior CB during rest	(143)
		70	13.8	Thalamic GABA/Cr is more negatively correlated with functional connectivity between the thalamus and CB at rest (fMRI combined with MRS)	(163)
	**MRS**	177	6.5	Decreased GABA/Cr, increased Glu/Cr	(156)

FA, functional anisotropy; GABA/Cr, gamma-aminobutyric acid to creatine ratio; Glu/Cr, glutamate to creatine ratio; MD, medial diffusivity; NAA, n-acetylaspartate.

**Table 4:** Regional findings: white matter and functional networks.

Region	Modality	Total N	Mean age (y)	Finding (ASD vs. TD)	Ref.
**Corpus Callosum (CC)**	**sMRI**	100	2.8	Increased volume, ASD females have increased volume compared to ASD males and TD controls	(90)
		80	4.1	Increased sub-region volumes in younger ASD males compared to older ASD males.	(71)
		83	7.2	Higher rates of hypoplastic CC	(69)
	**DTI**	78	2.5	In toddlers, increased FA and decreased MD in left CC	(102)
		181	3.2	Increased FA, increased AD in ASD females relative to TD females, decreased AD in ASD males relative to TD males	(92)
		109	3.5	Lower FA, higher MD, higher RD, higher AD	(93)
		194	2	Less age-associated FA increase	(91)
**Default Mode Network (DMN)**	**sMRI**	244	3.1	Increased volume and structural covariance in structures underlying the DMN	(144)
		137	12.6	Increased gyrification in structures underlying the DMN	(77)
	**fMRI**	96	10.9	Decreased functional connectivity with salience network during the Preparing to Overcome Potency task	(137)
		56	12.0	Reduced connectivity with the right superior frontal gyrus/paracingulate during rest	(106)
		119	13.4	Overconnectivity within the DMN during rest	(131)
		169	13.4	Female ASD group relative to male ASD group has greater functional connectivity between the DMN and central executive network during rest	(126)
		75	13.4	Overconnectivity with salience network and central executive network during rest	(105)
		106	4.6	Reduced connectivity in social cognitive subnetwork (between DMN, limbic, facial processing, and language networks) while sedated	(65)

AD, axial diffusivity; FA, functional anisotropy; MD, medial diffusivity; RD, radial diffusivity.

**Figure 1** shows the flowchart of the study selection process. Of the 2,794 initial records identified, 989 duplicates were excluded. The remaining 1,805 records were screened, with 797 being excluded based on title and abstract. The remaining 1,008 full-text records were assessed for eligibility, resulting in exclusion of 316 additional records, leaving 692 full-text records included in a primary evaluation. Following application of the secondary set of exclusion criteria, 520 records were excluded, and 172 final papers including 26 sMRI (65–90), 14 DTI (91–104), 41 fMRI (51, 105–144), 19 MRS (145–163), 20 fNIRS (164–183), 20 MEG (184–203), and 32 EEG (204–235) articles were considered in qualitative analysis.

### 3.1 Characteristics of selected papers

Characteristics of the included articles are presented in **Table 1**. The majority of studies followed a cross-sectional design (n = 152); a small number (n = 20) had longitudinal designs. The age distribution was right-skewed, with 32% of the selection having a mean age within 0-5 years (n = 55), and 48% of the selection having a mean age within 6-10 years old (n = 48). Approximately 38% of the articles had mean age fall between 11-15 years old (n = 66), and only 2% fell between 15-18 years old (n = 3). Distributions of mean age across modalities are presented in **Figure 2A**. Distributions of mean age between ASD and TD groups were similar across all seven modalities, as would be expected given frequent age-matching of groups. DTI, MEG, and sMRI showed distributions with mean ages in early childhood and early adolescence. EEG, MRS, and fMRI had mean ages of around 10, 12, and 13 years, respectively. The distribution of fNIRS papers ranged from 0 to 16 years. Aside from several outliers, the fMRI group distributions most closely approximated normality, with a peak in early adolescence.

Female participants were included in 82% of the studies (n = 141), 16% of studies included male participants only (n = 28), and 3 papers did not report sex. When both male and female participants were included, the percentage of female participants fell between 9.8% and 76.0%, with a mean value of 23.7%. IQ distribution was not reported in 53 studies (31%). IQ thresholds were used as exclusion criteria in 21 studies (12%), using thresholds of greater than either 75 or 80. About one quarter of studies (22%) matched ASD and TD groups on IQ. The ASD group had a lower average IQ score in 20% of papers that did not IQ-match participants. Distributions of mean IQ scores across modalities are presented in **Figure 2B**. Across imaging modalities, mean IQ values were lower for ASD groups than for TD groups. ASD groups showed wider distributions of mean IQ, and more outliers, with mean IQ values occasionally falling below 80. sMRI is the only modality with an ASD distribution containing mean IQ values below 60, and very few mean IQ values in any distribution outside sMRI fell below 80.

Most papers (n = 157) featured ASD/TD case-control study arms. Some articles compared infants with a family-history of ASD to infants without ASD family history to compare high- vs. low-likelihood cases (n = 13). Two papers contained only a TD group, where the authors assessed ASD traits in school-aged children. ASD traits in school-aged children may still reflect part of the autism spectrum, despite being undiagnosed or subclinical.

Nearly 60% of studies were carried out in North America (n = 100), while 25% were carried out in Asia (n = 44) or 12% in Europe (n = 20). Most studies used fMRI (n = 41), EEG (n = 32) or sMRI (n = 26) modalities. Less frequent modalities by number of papers included fNIRS (n = 20), MEG (n = 20), MRS (n =19), and DTI was the least frequent modality (n = 14). Sample sizes in MRS, MEG and fNIRS papers were small (n < 50 per modality), following our inclusion criteria. Most other modalities had a sample size between 51-100 participants (45% of studies). There were no CT, PET, SPECT, or ultrasound studies that met inclusion criteria for the qualitative analysis. We hypothesize that the modern non-invasive neuroimaging modalities included in this review are favored in the literature because they do not require radiation exposure and have improved spatial resolution.

### 3.2 Within-modality qualitative assessment

We identified differences between the ASD and TD neuroimaging profiles within each of the seven neuroimaging modalities. Structural MRI-based papers (n = 26, **Supplementary Table 3**; **Figure 3**) demonstrated larger overall subcortical gray matter volume in ASD groups. However, there were positive and negative differences in cortical gray matter volumes, i.e. both larger and smaller volumes in ASD groups compared to TD across various brain regions (65, 70, 78, 89). The amygdala, thalamus, hippocampus, and corpus callosum were the most prominent regions assessed in sMRI papers. Evaluation of cortical thickness, structural covariance, cerebrospinal fluid volume, gyrification, intracranial volume, surface area, and total brain volume did not reveal any consistent patterns (**Figure 3**).

DTI-based papers (n = 14, **Supplementary Table 4**; **Figure 4**) reported overall higher mean and radial diffusivity in ASD groups compared to TD groups (94, 96, 99, 102, 104, 108, 113, 133), and mixed directionality of fractional anisotropy (FA) findings (91, 92, 98, 100, 102, 103, 198). A small number of papers indicated higher global axial diffusivity in ASD (92, 93, 95, 99). Several DTI studies focused specifically on the corpus callosum, superior longitudinal fasciculus, and cingulum (91–93, 96, 102, 104). The corpus callosum (CC) appeared most often in DTI studies, with ASD groups showing overall higher axial diffusivity, notably higher FA values, lower mean diffusivity, and mixed radial diffusivity findings compared to TD groups (**Figure 4**) in this structure. Overall, findings suggest lower white matter microstructural integrity in the ASD group (**Figure 4B**).

Functional MRI-based papers (n = 41, **Supplementary Table 5**; **Figure 5**) also demonstrated substantial variability in findings. Within-network, regional connectivity and task-based functional activity patterns were highly mixed (**Figures 5A, B**), showing both increased and decreased connectivity or activation in various brain regions, without a clear pattern (51, 106, 113, 115, 116, 118, 123, 125–127, 131, 132, 135, 140–142). Studies with larger sample sizes indicate that ASD participants have higher within-region functional connectivity in subcortical regions and lower within-region connectivity in the default mode network and ventral attention network (**Figure 5A**) compared to TD groups. Between-network (**Figures 5C, D**) findings were also highly mixed (51, 105–108, 111–114, 116, 118, 120, 121, 124, 126, 128–131, 134–137, 141, 163). There was an abundance of between-network connectivity findings in the amygdala, thalamus, default mode network, insula, and prefrontal cortex (105–108, 111, 113, 114, 118, 121, 126, 128, 134, 136, 137, 163). **Figures 5C, D** shows higher between-region functional connectivity across these brain regions. Between-network connectivity involving the amygdala and primary visual cortex (V1) is mostly decreased in ASD groups, whereas between-network connectivity involving the imitation network, language regions, paracingulate cortex, and thalamus is mostly increased in ASD groups compared to TD groups.

MRS-based papers (n = 19, **Supplementary Table 6**; **Figure 6**) overall reported higher metabolite markers of excitatory neurotransmission (145–147, 149, 150, 154–157, 159), lower metabolite markers of inhibitory transmission (156, 161, 162, 198), and lower markers of overall brain health in ASD groups (145, 147, 151–153, 158, 160). ASD groups appear to have lower markers of neuronal health such as n-acetylaspartate (NAA), choline-containing compounds, and creatine (**Figure 6A**). The anterior cingulate cortex (ACC) figured the most prominently in MRS papers (146, 152, 155–157, 160). Contrary to global findings, findings specified for region and MRS metric suggested that markers of excitation and inhibition were mixed and heterogenous across brain regions (**Figure 6**).

fNIRS-based papers (n = 20, **Supplementary Table 7**; **Figure 7**) demonstrated overall lower oxygenated hemoglobin (oxy-Hb) levels in ASD groups (164–168, 175–177, 179, 181), with one report indicating increased rightward lateralization (180). The ASD groups demonstrate a notably lower oxygenated hemoglobin levels in the dorsolateral prefrontal cortex (DLPFC), prefrontal cortex (PFC), and various temporal regions compared to TD groups (167, 168, 172–174, 177, 178). Lower oxy-Hb across brain regions indicates decreased task-related activation in the ASD group overall (**Figure 7**).

The lack of spatial specificity in these waveform-based imaging modalities prevented us from producing brain maps for MEG or EEG. MEG-based papers (n = 20, **Supplementary Table 9**; **Figure 8A**) reported decreased auditory ERP amplitude in ASD (188, 202). A small number of MEG studies suggested shorter ERF latency and higher total power (188, 196, 197, 201, 203). Contrary to global findings suggesting higher overall MEG power, region and domain-specific power/frequency findings in MEG papers are mixed; with most cortical regions showing higher power, while investigations of frontal regions and the whole brain noted lower activity and power (**Figure 8A**). Additionally, there are mixed region-specific connectivity and ERF findings. However, there is consensus that ASD participants have lower amplitude to auditory-evoked fields.

Consistently with MEG, EEG-based papers (n = 32, **Supplementary Table 8**; **Figure 8B**) reported decreased auditory and visual ERP amplitude and decreased alpha band power (208, 210, 218, 225–229, 232). ASD groups demonstrated higher whole-brain EEG connectivity (**Figure 8B**). Likewise, a decrease is seen in EEG frequency/power in diffuse anterior, central, and posterior regions of the brain. In addition, **Figure 8B** indicated mixed findings for EEG frequency/power in the whole brain. Lower auditory ERP amplitude is also observed.

### 3.3 Multimodal region-specific findings

Across modalities, notable patterns emerged when assessing all findings specific to cortical gray matter regions (**Table 2**). In the anterior cingulate cortex, ASD groups demonstrated higher markers of neuronal excitation (glutamate, Glu; and glutamine, Gln) (146, 157, 159) and higher functional connectivity within the thalamus and ventrolateral prefrontal cortex compared to TD groups (136). The insula in ASD showed altered functional connectivity with various cortical regions in resting state and task-based paradigms (107, 118, 121). The right insula specifically showed increased white matter volume and decreased cortical thickness (66, 102), as well as lower mean diffusivity in the insular cortex (102). Findings for the prefrontal cortex showed altered resting state functional connectivity with various cortical regions (108, 111, 113, 134, 136, 163). This region also exhibited lower oxy-Hb signal during administration of task involving social stimuli (167, 168). Studies reported less gray matter in the left anterior PFC (78) and lower markers of neuronal health in the medial PFC (145).

When assessing all findings specific to subcortical gray matter regions, the amygdala, thalamus and cerebellum were described more frequently than other regions (**Table 3**). Findings for the amygdala demonstrated larger volume in ASD groups compared to TD groups, even in early infancy (79, 85, 86). Additionally, amygdala-specific findings suggested decreased regional functional connectivity and between-network connectivity with several cortical structures implicated in emotional processing, including the medial prefrontal cortex, striatum, thalamus, cingulate cortex, and cerebellum, as well as the inferior occipital gyrus (114, 133, 134). One study found lower NAA levels in the amygdala in ASD participants compared to TD participants (158). The thalamus showed increased functional connectivity with subregions of the auditory cortex, Heschl’s gyrus, primary sensory cortex and prefrontal cortex (128, 129, 131). The cerebellum demonstrated decreased resting-state functional connectivity with amygdala, and locally, as indicated by decreased regional homogeneity in the right anterior cerebellum (134, 143). The cerebellum likewise demonstrated decreased GABA/Cr ratio and a negative correlation between GABA/Cr ratio and functional connectivity with the thalamus (156, 163). Only one study reported volume differences in the cerebellum across age in ASD compared to TD groups (87).

The region-specific findings for white matter structures and functional networks are presented in **Table 4**. Findings for the corpus callosum in ASD demonstrated both higher and lower fractional anisotropy, and a various age- and sex- associated differences in volume, fractional anisotropy, and axial diffusivity (91–93, 102, 104). Volumetric findings for the corpus callosum were mixed: both larger volumes and higher rates of hypoplastic corpus callosum were reported (69, 90). Findings for the default mode network showed significantly altered connectivity in both directions with the salience network (105, 137), as well as larger volume in default mode network structures (144).

## 4 Discussion

The aim of this literature review was to determine patterns of differences in brain structure, function and neurochemistry between autistic and typically developing youth, and to assess converging patterns and inconsistencies within and across multiple neuroimaging modalities. In our literature synthesis of 172 papers, we found considerable heterogeneity in reported findings across all neuroimaging modalities but were able to identify some consistent patterns of difference between ASD and TD groups.

ASD groups had larger overall subcortical gray matter volume compared to TD, as well as higher mean and radial diffusivity. Literature further indicates that ASD youth had higher metabolite markers of excitatory neurotransmission, lower metabolite markers of inhibitory neurotransmission, and lower markers of overall brain health. EEG and MEG studies showed that ASD youth had decreased auditory ERP amplitude, and visual ERP amplitude in EEG-studies only. The anterior cingulate cortex, insula, prefrontal cortex, amygdala, thalamus, cerebellum, corpus callosum, and default mode network appeared consistently as regions of interest across all seven neuroimaging modalities.

### 4.1 Within-modality findings

Our findings corroborate a substantial body of existing literature (236–238) suggesting that the neurobiology of ASD is highly heterogeneous. Findings across every modality were notably inconsistent, as demonstrated by mixed findings in the bubble plots and brain maps. Differences in a neuroimaging metric (e.g. connectivity, activation, volume) between groups could rarely be defined in one direction. These issues were particularly salient in functional MRI papers, where patterns in connectivity, resting-state activation, and task-based activation were different in nearly every publication. A similar picture arose for structural MRI, with a wide variety of regions and metrics reported across studies.

These inconsistencies within imaging modalities may be explained by multiple factors. Firstly, there was considerable heterogeneity in the study populations, as illustrated by differences in the mean age and age ranges within and across neuroimaging modalities. While our secondary exclusion criteria (limiting participants to under 18-year-olds and case/control arms to N>25) theoretically decreased the heterogeneity of our samples and contributed to a lower risk of spurious findings due to very small sample sizes, enforcing a larger sample size potentially increased the heterogeneity of our findings by adding heterogeneity into ASD symptomatology. However, the innate heterogeneity of ASD will be present regardless of sample size, and we believe the importance of reducing the risk of spurious findings to be greater. Comparisons between specific age groups were complicated by large variations in analytical choices adopted by research groups. Issues related to the handling of sex/gender and IQ were common: a substantial proportion of studies had only male participants or a minority of female participants, and many studies did not report in IQ or excluded participants with IQ scores below 75 or 80 points. IQ measures were not evenly distributed between ASD and TD groups, indicating differences in selection into study samples. Since differences in cognitive performance have been shown to be related to autism and the broader autism phenotype (239), not matching on IQ introduces a considerable confound. Another potential method of controlling for cognitive performance-linked elements of ASD could be to match participants on parental measures of cognitive performance, or education level as a proxy for parental IQ when participant IQ cannot be measured. Further, race and ethnicity were rarely considered in the analyses, nor reported in the manuscripts. Not considering important factors such as ethnicity, sex, and IQ in study designs and analyses could introduce selection and confounding bias into analyses on neurobiological phenotypes, which may result in different findings reported in many of the studies.

Moreover, a decade of neuroimaging research has contributed significantly to the growth of analytical methods for complex neuroimaging data: the field has moved from mostly region of interest and hypothesis-driven studies to focus more on whole brain, hypothesis-free analyses. Both study types have the potential to introduce bias and inconsistency into reported findings. There are clear advantages statistically in the pre-hoc selection of brain regions of interest in the analysis of large quantities of neuroimaging data (240), though it is evident that such selection frequently depends on *a priori* assumptions about brain and behavior which can lead to potential bias in theoretically favored regions (241), at the expense of theoretically disfavored regions. Alternatively, whole-brain approaches, while not depending on an *a priori* selection of brain regions, have been known to unreliably delimit brain regions compared to ROI techniques (242, 243), and apply inconsistent corrections for multiple comparisons (244).

Methods for DTI tractography have improved (245), EEG and MEG technology has advanced (245), and there is increased use of MRS, MEG and NIRS modalities. The advancement of technology and analytical methods across the past decade adds another layer of variability into reported findings (246–248). That said, if robust differences were to be present between ASD and TD groups at the level of resolution of our current neuroimaging methods, emerging technologies and methods should result in an increase in the signal to noise ratio, with the result of strengthening and supporting earlier findings.

Inconsistency in image processing and acquisition within modalities adds another layer of variability into the reported findings. There is a broad range of methodological choices in this respect which might drive variability in results including the length of scans, any inter-operator variability in data collection, the much-debated use of global signal regression in image preprocessing, the type of artifact removal, the selection of image processing software, and any variability in image processing pipeline. It is nearly impossible to find two neuroimaging studies exactly alike along these domains, and each unique decision made in image collection, preprocessing, and analysis stands to drive variability in results across studies.

Alternatively, the differences in neuroimaging profiles described in this review may reflect actual biological differences in brain structure and function of autistic youth, rather than result from variability in methods. Observations within modalities also uncovered compelling consistencies. Structural imaging findings revealed that autistic participants have larger subcortical gray matter volumes and higher mean and radial diffusivity compared to typically developing peers (65, 70, 94, 96, 99, 102, 104, 108, 113, 133). Functional MRI findings hinted at patterns of lower between-network connectivity in areas related to the amygdala and V1 (114, 121, 134) but increased between-network connectivity in networks related to the imitation network, language regions, paracingulate and thalamus (106, 113, 116, 127, 129, 131). Additionally, autistic youth have higher metabolite markers of excitatory neurotransmission (145–147, 149, 150, 154–157, 159), lower metabolite markers of inhibitory neurotransmission (156, 161, 162, 198), lower markers of overall brain health and metabolism (145, 147, 151–153, 158, 160), decreased EEG-based visual ERP amplitude (218, 227, 228), and both EEG- and MEG-based decrease in auditory ERP/ERF amplitude (188, 202, 208, 210, 211, 232). These findings align with several currently existing theories on the causes of ASD, including the excitation/inhibition theory, which posits that a combination of genetic and environmental variables contribute to increased glutamatergic and/or decreased GABAergic transmission across the brain (35, 36). We also found evidence to support existing theories (249–251) that autistic youth have atypical connectivity patterns, although we were not able to identify consistent patterns of directionality. Our findings further indicated that gross structural changes or activity patterns are not likely to contribute to the neurobiological phenotype of ASD, but rather altered microstructural connectivity is more likely to be part of the phenotype of ASD (252, 253).

These findings might be taken together to motivate investigation into the relationship between genetics, cerebral microstructure, neuronal health, excitation/inhibition, and functional connectivity in autistic youth. Employing multimodal approaches to explore these concepts could provide complementary insights into both structural and functional findings. Additionally, longitudinal multimodal research may clarify whether the structural and functional differences observed in autistic youth are unidirectional or bidirectional. Whether the differences in neuroimaging profiles between autistic and neurotypical participants in this body of research translates to actual differences in neurobiological and behavioral phenotypes across the autism spectrum cannot be easily determined without considering the myriad biological, environmental, stochastic, and confounding factors at play.

### 4.2 Region-specific findings

Several brain regions appeared numerous times in the literature and displayed consistent differences between autistic and typically developing youth. These regions include the anterior cingulate cortex, insula, prefrontal cortex, amygdala, thalamus, cerebellum, corpus callosum, and default mode network. We found that autistic participants have altered neurochemical expression and functional connectivity in the ACC, which is consistent with post-mortem (254), fMRI (255), and multi-modal imaging (256) research that connects altered ACC cytoarchitecture and signaling with ASD diagnosis, autism symptoms and altered connectivity. Atypical connectivity patterns are also at play in the insula and prefrontal cortex. Impaired insular function could affect reward processing; additionally, abnormal insula activity has been implicated in social deficits related to ASD (257). The medial prefrontal cortex, a key hub in the default mode network, also plays an important role in cognitive functions like working memory, planning, inhibition, and social behavior (258, 259). Structural and functional abnormalities in other prefrontal subregions including the dorsolateral prefrontal cortex (260) and orbitofrontal cortex (261) are often associated with repetitive behaviors and social impairment in autistic individuals. Similarly, altered connectivity between the cortex and subcortical regions like the amygdala and thalamus are closely linked with ASD symptoms. Our findings that amygdala volume and connectivity are altered in autistic participants is consistent with over 20 years of research supporting “the amygdala theory of autism,” (262) which posits that structural and functional amygdala abnormalities explain social deficits in ASD. However, it is unlikely that changes to this region alone account for such a broad array of symptoms and severity (263). In the thalamus, changes in connectivity to other brain regions could affect sensory processing and attention, which likely contribute to core ASD symptoms (264). Cerebellar differences are also likely to affect key ASD features, such as motor activity, language ability, and higher cognitive functions (265). Our findings that autistic youth have structural alterations in the corpus callosum is consistent with research indicating that CC abnormalities are related to ASD symptoms; however, the directionality of these volumetric differences is inconsistent (266). Additional longitudinal research is necessary to tease apart how structural and functional abnormalities develop in autistic youth.

It is important to consider these region-specific findings may not be sensitive nor specific to ASD. The brain regions that we describe are also frequently found in multiple mental health and neurological conditions. Thus, these may reflect more general neurocognitive and social functions that are not necessarily specific to ASD. Autistic youth are at higher risk for internalizing psychopathology (267, 268), sleep problems (269), and attention problems (270) than their neurotypical peers; co-occurring psychopathology in autistic participants could contribute to the heterogeneity of findings. Further, some neuroimaging modalities are more prone to detect signal in certain brain regions: examples include fNIRS, where cap placement and hair interference can influence the accurate measurement in specific brain regions (i.e., the frontal and temporal compared to the parietal and occipital lobes) (271), or MRS, where researchers carefully specify a confined region of interest prior to scanning, often based on clear hypotheses (272, 273).

The converging patterns in neuroimaging profiles based on our literature review may be helpful in fine-tuning existing theories, but it is inappropriate to assume that all autistic youth have similar neuroimaging phenotypes, or that neuroimaging findings are sufficient for making an ASD diagnosis. The heterogeneity in the behavioral phenotype of ASD likely plays a major role in the heterogeneity of the findings within and between studies.

### 4.3 Strengths and limitations

This literature review aimed to provide a birds-eye view of the wealth of neuroimaging research literature investigating neurobiological mechanisms of ASD and to identify consistencies across different modalities. A major strength of this study is that we included seven different imaging modalities and were able to carry out complementing qualitative syntheses of the literature. Region-specific brain maps and bubble plots provided a more quantitative counterpart to the region-specific findings. We expected that the weight of the findings across different neuroimaging modalities would reinforce each other and have some convergence. Our study has a number of limitations that should be carefully considered in the interpretation of our findings. First, our literature review does not follow the validated structure of a systematic or scoping review, and we did not conduct a formal quality assessment on the articles we included in analysis. Still, we followed recommendations for systematic gathering of literature and synthesizing the evidence rigorously. Second, publication bias operates at multiple levels: statistically significant positive and negative (excluding null) findings tend to be reported in the literature itself. Furthermore, in order to organize findings across such a scope of literature, we elected to largely exclude statistically insignificant and null findings. Notably, we applied a qualitative assessment to summarize the evidence. This is in itself not a limitation, but we will note that effect estimates and pooled statistical significance underlying the synthesis of the literature are not reflected in this review.

Finally, reconciling region-of-interest and whole brain approaches in neuroimaging literature is a challenge for the ASD field as well. There is a noteworthy bias with respect to the selection of imaging modality and specific regions-of-interest from which to extract neuroimaging signal. Selection of a neuroimaging modality and regions of interest should always be justified by theoretical assumptions and previous literature. It is a further, more general limitation and source of variability in any neuroimaging research that regions-of-interest, whether established by segmentation or parcellation, cytoarchitectonics, historical neuroanatomy studies, brain atlas or otherwise; these specified regions are not defined consistently across studies.

### 4.4 Implications

Our work reveals crucial implications for future research in ASD. The heterogeneity we observed, especially in the MRI-based studies, was substantial enough to motivate a closer look at sources of potentially avoidable variability in research. Such sources include differences in sample selection and demographics, ASD diagnostic criteria, cognitive ability and IQ, comorbid symptoms or diagnoses, image processing, and the inconsistent use of sedation in the MRI scanning of children with ASD.

Aside from these research implications, our work has potential clinical implications. Findings on the variability of structural measures in ASD compared to TD across the brain are relevant to both the relationship between the structural and electrical levels of analysis in the brain, and to the application of the novel therapy Repetitive Transcranial Magnetic Stimulation (rTMS). rTMS is a noninvasive electrical impulse-based therapy with established use in major depression (274, 275), gaining in popularity and clinical approval in other mental health disorders (276). In relation to EEG, and MEG, principles of electrophysics suggest that structural characteristics of underlying brain tissue at the scalp modulate the dynamic electrical field from underlying neuronal activity (277). Targets selected for rTMS would thus depend on structural properties of the brain, because these targets depend on wave properties of the underlying electrical field. Therefore, variable findings on structural differences in ASD suggest that substantial variability exists in the brain structure and electrical activity profiles of ASD, and this variability may be leveraged for personalized targeting in rTMS.

Ongoing work exists in the application of rTMS to reduce the symptomatology of ASD when these symptoms are burdensome or harmful to the individuals living with these symptoms (278, 279). This therapy requires specific brain targets, which most frequently are selected on theory and literature. There is potential for identified regions in this review, particularly those which potentially relate to the neurobiology of ASD across structural, functional, neurochemical domains to inform the selection of targets for rTMS therapies. Still, it is crucial to consider that we have not linked the brain regions in this literature review to any ASD symptomatology. Current ongoing research is exploring the relationship between brain regions and ASD symptomatology at the individual level by precision rTMS neuromodulation.

### 4.5 Future directions

Future studies may build on our initial review to perform meta-analyses on more targeted questions that allow pooling of the effect estimates, which was not possible within the multimodal scope of this current review. The most rigorous approach would be to obtain raw data from all the studies and perform meta-analyses across modalities.

Furthermore, we did not involve ASD stakeholders in the making of this literature review. ASD and neurodiversity stakeholders in research and wider communities have in past years increasingly called for inclusion of stakeholders in research, to guide the questions that are asked in research and safeguard the needs of autistic and neurodiverse persons in research. We tried to incorporate perspectives from previously published work by autistic scholars and ASD stakeholders as much as possible but recognize that this does not equate the inclusion of original voices. A more comprehensive inclusion of input and commentary from those within the ASD community should be considered an essential element of future research examining the neurobiology of ASD.

It is evident that the approaches to quantify both diagnosis and severity of ASD were not consistent across studies. ADOS and ADI-R serve as the “gold standard” for diagnosis and assessment, tending to produce high levels of inter-rater reliability and agreement with clinician diagnosis (280–282). Nevertheless, it is quite clear that this model is not always followed rigorously, and in many cases not followed at all. In future work a clearer selection of papers based on ASD diagnostic metric and method is needed. Similarly, it is essential to note that we opted to include 15 articles in our primary analysis that did not divide participants into formally diagnosed case/control groups. This was a decision meant to permit a synthesis which captures both participants too young for diagnosis (family history), and a broader spectrum of the behavioral phenotype of ASD, potentially capturing subclinical levels of autism (ASD traits). In future work, a more careful evaluation of ASD diagnostic metric and method, and relative symptom level is required to develop a more sophisticated understanding of the ASD brain phenotype in relationship to the ASD behavioral phenotype. Moreover, future work could investigate the relationship between neuroimaging outcomes and behavioral features such as sensory sensitivity, social behavior, and motor symptoms. We did not address these phenotypic traits in our qualitative analysis, but a deeper understanding of the relationship between brain and behavior would undoubtedly strengthen our understanding of ASD.

The expansion of the investigation to include literature across the lifespan is needed to develop a broader understanding of the ASD brain phenotype. Specifically, within-subject longitudinal research that tracks the neurodevelopment of ASD well beyond our age cutoff of 18 years would be the optimal approach for investigating the neurobiology of ASD across the lifespan. Considering prenatal development by longitudinal or cross-sectional study would also offer insight into a crucially formative developmental phase, which we did not consider in this analysis.

### 4.6 Conclusion

ASD is a neurodevelopmental disorder marked by heterogeneity in its neuroimaging profile: inconsistent results are frequently seen within imaging modalities, comparable study populations and research designs. Despite variability, converging features do emerge, especially when considering brain region, age, imaging modality, and imaging modality metric. Within modalities, consistent differences between the ASD and TD imaging profile were found at both the global and region-specific levels.

Considering the rich neurodiversity in the ASD community, it is not surprising to find rich diversity in the range of neurobiological phenotypes associated with autism. Heterogeneity remains the rule, though multimodal methods show promise for elucidating the complexities associated with ASD phenotypes. In any case, multimodal neuroimaging is positioned to produce interesting and novel discoveries in the coming decade of investigations to advance our collective understanding of ASD, behavioral phenotypes, and neurodevelopment more broadly.
