# Supplementary material for: Heterogeneity and convergence across seven neuroimaging modalities: a review of the autism spectrum disorder literature
Source: Front Psychiatry. 2024 Oct 16;15:1474003. doi: 10.3389/fpsyt.2024.1474003 (PMC11521827; doi:10.3389/fpsyt.2024.1474003)
Supplement: Supplementary file 1 [file DataSheet1.docx]

Supplementary Material

**Heterogeneity and convergence across seven neuroimaging modalities: a review of the autism spectrum disorder literature**

Halliday et al.

Index

[Supplementary Table 1. PubMed Search Terms. 2](#_Toc176348693)

[Supplementary Table 2. Inclusion Numbers Per Modality. 3](#_Toc176348694)

[Supplementary Table 3. Structural MRI (sMRI) Findings. 4](#_Toc176348695)

[Supplementary Table 4. DTI Findings. 9](#_Toc176348696)

[Supplementary Table 5. fMRI Findings. 12](#_Toc176348697)

[Supplementary Table 6. MRS Findings. 19](#_Toc176348698)

[Supplementary Table 7. fNIRS Findings. 22](#_Toc176348699)

[Supplementary Table 8. MEG Findings. 25](#_Toc176348700)

[Supplementary Table 9. EEG Findings. 28](#_Toc176348701)

[Supplementary Figure 1. Subcortical Brain Maps. 32](#_Toc176348702)

[References 33](#_Toc176348703)

# Supplementary Table 1. PubMed Search Terms.

| **Domain** | **Terms** |
| --- | --- |
| ASD *all searches contained terms for ASD, followed by terms listed in table | ("Autis*"[tiab] OR "Aspergers"[tiab] OR "ASD"[tiab] OR "PDD-NOS"[tiab] OR "Autism Spectrum Disorder" [Mesh] OR "Pervasive Developmental Disorder Not Otherwise Specified"[tiab] OR "Autistic Disorder"[Mesh]) NOT (“Adult*”[tiab] OR “Meta-Analysis”[tiab] OR “Review”[tiab] OR “case stud*”[tiab] OR “clinical trial*”[tiab] OR “Editorial*”[tiab] OR “Comment*”[tiab] OR "atrial septal defect*"[tiab] OR "amorphous solid dispersion*"[tiab] OR "average surface distance"[tiab] OR "adjacent segment disease"[tiab] OR "medicat*"[tiab] OR "mutat*"[tiab] OR "gene*"[tiab] OR "deep learning"[tiab] OR "computer assisted diagnos*”[tiab] OR "learn*"[tiab] OR "machine learning"[tiab]) |
| sMRI | AND ("Magnetic Resonance Imaging"[MeSH] OR "MRI"[tiab] OR "SMRI"[tiab] OR "Structural Magnetic Resonance Imaging"[tiab] OR "Structural MRI"[tiab] OR "Grey Matter Volume"[tiab] OR "grey Matter"[tiab] OR "white matter volume"[tiab] OR "white matter"[tiab] or "total brain volume" [tiab] OR "cortical thickness"[tiab] OR "voxel-based morphometry"[tiab]) NOT ("fMRI"[tiab] OR "functional MRI"[tiab] OR "rsMRI"[tiab] OR "resting state MRI"[tiab] OR "near-infrared spectroscopy" OR "NIRS") |
| DTI | AND ("Diffusion Tensor Imaging"[MeSH] OR "Diffusion Weighted Imaging" [tiab] OR "DTI" [tiab] OR "DWI" [tiab] OR "Diffusion Tensor MRI*"[tiab] OR "Diffusion Tensor"[tiab] OR "fractional anisotropy"[tiab] OR "mean diffusivity"[tiab]) |
| fMRI | AND (“functional neuroimaging"[MeSH] OR "MRI"[tiab] OR "fMRI"[tiab] OR "Magnetic Resonance Imaging"[MeSH] OR "functional MRI"[tiab] OR "functional magnetic resonance imaging"[tiab] OR "rsMRI"[tiab] OR "resting state MRI"[tiab] OR "resting state magnetic resonance imaging"[tiab] OR "resting state fMRI"[tiab] OR "rsfMRI"[tiab] OR "resting state functional magnetic resonance imaging"[tiab]) NOT ("sMRI"[tiab] OR "structural MRI" OR "near-infrared spectroscopy" OR "NIRS") |
| MRS | AND ("MRS"[tiab] OR "magnetic resonance spectroscopy"[MeSH] OR "magnetic resonance spectroscopies"[tiab] OR "MR spectroscop*"[tiab]) |
| fNIRS | AND (“NIRS"[tiab] OR "near-infrared spectroscopies"[tiab] OR "Near-Infrared Spectroscopy"[tiab] OR "NIR Spectroscopy"[tiab] OR "NIR Spectroscopies"[tiab] OR "Spectroscopies, NIR"[tiab] OR "Spectroscopy, NIR"[tiab] OR "Spectrometry, Near-Infrared"[tiab] OR "Near-Infrared Spectrometries"[tiab] OR "Near-Infrared Spectrometry"[tiab] OR "Spectrometries, Near-Infrared"[tiab] OR "Spectrometry, Near Infrared"[tiab]) |
| MEG | AND ("Magnetoencephalography"[MeSH] OR "Magnetoencephalogram"[tiab] OR "Magnetoencephalograms"[tiab] OR "MEG"[tiab]) |
| EEG | AND ("electroencephalography"[MeSH] OR "EEG"[tiab]) |
| CT | AND ("CT"[tiab] OR "Computerized Tomography"[tiab] OR "Tomography, X-Ray Computed"[MeSH]) |
| PET | ("PET"[tiab] OR "PET scan*"[tiab] OR "positron emission tomography"[MeSH] OR "positron-emission tomography"[MeSH] OR "positron emission tomography scan*"[tiab]) NOT "dog"[tiab] |
| SPECT | ("Tomography, Emission-Computed, Single-Photon" [MeSH] OR "SPECT"[tiab]) |
| Ultrasound | ("ultrasound"[MeSH] OR "ultrasound"[tiab] OR "sonogra*"[MeSH] OR "sonogra*"[tiab]) |

ASD, autism spectrum disorder; CT, computed tomography; DTI, diffusion tensor imaging; EEG, electroencephalography; fMRI, function magnetic resonance imaging; fNIRS, functional near-infrared spectroscopy; MEG, magnetoencephalography; MeSH, medical subject heading; MRS, magnetic resonance spectroscopy; PET, positron emission tomography; SPECT, single-photon emission computed tomography; sMRI, structural magnetic resonance imaging; tiab, title or abstract.

Supplementary Table 2. Inclusion Numbers Per Modality. Each article is assigned to one modality category according to the search result it originally appeared in. Some articles contained findings relevant to multiple modalities. The number of articles that are relevant to other modalities are indicated in parentheses.

| **Imaging Modality** | **Identified Through Database Search** | **Included in Title/Abstract Screening** | **Assessed for Eligibility** | **Included in full reading** | **Included in Qualitative Analysis (Overlapping Modality)** |
| --- | --- | --- | --- | --- | --- |
| **sMRI** | 1831 | 892 | 527 | 357 | 26 (3 DTI) |
| **fMRI** |  |  |  |  | 41 (3 DTI, 1 MRS, 1 EEG) |
| **DTI** | 144 | 144 | 93 | 74 | 14 (3 sMRI, 3 fMRI, 1 MEG, 1 MEG/MRS) |
| **MRS** | 64 | 59 | 30 | 27 | 19 (1 MEG/DTI) |
| **fNIRS** | 38 | 36 | 29 | 23 | 20 |
| **MEG** | 101 | 84 | 58 | 58 | 20 (1 DTI, 1 DTI/MRS) |
| **EEG** | 433 | 434 | 258 | 153 | 32 (1 fMRI) |
| **CT** | 113 | 92 | 1 | 0 | 0 |
| **PET** | 33 | 33 | 9 | 0 | 0 |
| **SPECT** | 7 | 3 | 0 | 0 | 0 |
| **Ultrasound** | 30 | 28 | 3 | 0 | 0 |
| **Total** | **2794** | **1805** | **1008** | **692** | **172** |

# Supplementary Table 3. Structural MRI (sMRI) Findings.

| **Cross-sectional** | | | | | | | | |
| --- | --- | --- | --- | --- | --- | --- | --- | --- |
| **Author** | **Year** | **Study Design; Task/Stimulus** | **N** | **Age** | **Sex** | **Region** | **ASD Metric** | **Outcome** |
| Pote et al. (1) | 2019 | Cross-sectional; Sleep | Total: 50  FH ASD: 24  NFH ASD: 26 | FH: 4.79 months  NFH: 4.81 months | FH: 13 (54)  NFH: 14 (54) | UK | SCQ, DAWBA | FH infants have larger cerebellar volumes. |
| Shiohama et al. (2) | 2022 | Cross-sectional; Sleep | Total: 126  ASD: 81  TD: 45 | ASD: 2.0 (0.75-3.0)  TD: 1.8 (0.75-3.0) | ASD: 11 (14)  TD: 7 (16) | USA | DSM-IV or DSM-5 | ASD group has smaller nucleus accumbens volume compared to non-ASD. Total gray matter, total white matter and total intracranial volume are not significantly different between ASD and non-ASD groups. ASD group has larger cerebral ventricle and choroid plexus volumes than non-ASD group. ASD has decreased cortical thickness of the left caudal anterior cingulate cortex, but increased cortical thickness of the right medial orbitofrontal cortex compared to non-ASD. |
| Xiao et al. (3) | 2014 | Cross-sectional; Sedation | Total: 78  ASD: 50  TD: 28 | ASD: 2.5 (0-8)  TD: 2.35 (0-8) | ASD: 8 (16)  TD: 6 (21) | China | DSM-IV-TR, CARS, ADI | ASD toddlers have increased gray and white matter volume overall. ASD toddlers have increased white matter volume in right superior temporal gyrus, left middle temporal gyrus, right insula, and right Heschl’s gyrus. Gray matter volumetric increase is more localized in right superior temporal gyrus of temporal lobe. |
| Zhang et al. (4) | 2023 | Cross-sectional; Sedation | Total: 100  ASD: 50  TD: 50 | ASD: 2.76 (2-4)  TD: 2.77 (2-4) | ASD: 14 (28)  TD: 17 (34) | China | DSM-5 and Gesell test | ASD group has increased volume in the corpus callosum. ASD females specifically have increased corpus callosum volume compared to ASD ales and the TD group. |
| Nordahl et al. (5) | 2020 | Cross-sectional; Sleep | Total: 420  ASD: 300  TD: 120 | ASD: 3.1 (2.0-5.0)  TD: 2.6 (2.0-4.4) | ASD: 91 (30)  TD: 57 (48) | USA | ADOS-2, ADI-R | ASD group has larger right amygdala volume than TD group, specifically an ASD subgroup with high psychopathology and moderate impairment. |
| Guiliano et al. (6) | 2018 | Cross-sectional; Sedation | Total: 80  ASD: 40  TD: 40 | ASD: 4.1 (2.8-5.8)  TD: 4.1 (2.0-6.0) | ASD: 20 (50)  TD: 20 (50) | Italy, USA | DSM-5, ADOS-G | No overall difference in total and sub-region corpus callosum (CC) volumes between TD and ASD groups. There are increased CC volumes in younger ASD males. |
| Bai et al. (7) | 2023 | Cross-sectional; Sedation | Total: 106  ASD: 67 TD: 39 | ASD: 4.62 (2-8)  TD: 4.61 (2-8) | ASD: 19 (28)  TD: 14 (36) | China | Clinical assessment, ADOS-2, ABC | ASD group has larger total intracranial volume and larger volume of the right medial superior frontal gyrus and left fusiform gyrus. ASD has significantly less connected social cognitive subnetwork, including the default mode network (medial prefrontal cortex, precuneus, anterior cingulate gyrus, hippocampus), limbic (amygdala, olfactory cortex, orbitofrontal cortex) and facial processing regions (fusiform gyrus, inferior occipital gyrus) and language regions (superior temporal gyrus and temporal pole). |
| Duan et al. (8) | 2020 | Cross-sectional; TD group watched videos (rest), ASD group sedated | Total: 78  ASD: 40 TD: 38 | ASD: 5.25 (3.61-7.57)  TD: 5.64 (2.78-6.78) | ASD: 8 (20) TD: 10 (26) | China | DSM-5, ADOS | ASD group has decreased inter-hemispheric structural covariation and increased intra-hemispheric structural covariation in subcortical regions, specifically the thalamus, nucleus accumbens, and pallidum. |
| Erbetta et al. (9) | 2015 | Cross-sectional; Sedation | Total: 83  ASD: 41  TD: 42 | ASD: 7.2 (2-15.4)  TD: 7.2 (1.5-14) | ASD: 12 (29) TD: 21 (50) | Italy | DSM-IV, ADOS-G, ADI-R | There are more structural abnormalities among participants in the ASD group, specifically higher rates of mega cisterna magna and hypoplastic corpus callosum. |
| Yang et al. (10) | 2016 | Cross-sectional; Rest | Total: 101  ASD: 60 TD: 41 | ASD: 8.35 (4.49-11.99)  TD: 8.83 (4.75-12.16) | ASD: 0 (0)  TD: 0 (0) | USA | DSM-IV | ASD group does not demonstrate age-related gray matter reduction in the superior temporal sulcus and superior parietal gyrus that is seen in TD group. ASD group also has increased age-related gyrification in the superior parietal gyrus, paracingulate gyrus, middle frontal gyrus, and superior temporal gyrus. |
| Sharp et al. (11) | 2023 | Cross-sectional; Rest | Total: 6933  Low SRS: 2791  Mid SRS: 2417  High SRS: 1797 | Low SRS: 9.88 (9-10)  Mid SRS: 9.90 (9-10)  High SRS: 9.90 (9-10) | Low SRS: 1471 (45)  Mid SRS: 1145 (37)  High SRS: 690 (21) | USA | SRS | Did not find strong evidence for an association of autism trait severity with differences in subcortical morphology. |
| D'Mello et al. (12) | 2015 | Cross-sectional; Rest | Total: 70  ASD: 35 TD: 35 | ASD: 10.4 (8-13) TD: 10.4 (8-13) | ASD: 5 (14) TD: 9 (26) | USA | DSM-IV, ADOS-G, ADI-R | ASD group has smaller gray matter in Crus I/II and larger vermis VIIIA and vermis VIIIB volumes compared to TD. ASD group has significantly more CSF than TD group. |
| Duerden et al. (13) | 2014 | Cross-sectional; Rest | Total: 63  ASD: 33  TD: 30 | ASD: 10.6 (7-15)  TD: 10.7 (7-15) | ASD: 4 (12)  TD: 4 (13) | Canada | ADOS-G, ADI-R | Thalamic nuclei volumes are larger in ASD compared to TD. Total cerebral volume, grey and white matter volume does not differ between ASD and TD groups. There are no differences in surface area and cortical thickness between ASD and TD groups. |
| Sussman et al. (14) | 2015 | Cross-sectional; Watching movie (rest) | Total: 210  ASD: 72  TD: 138 | ASD: 4-18  TD: 4-18 | ASD: 11 (15)  TD: 22 (16) | Canada | ADI-R, ADOS | ASD males have smaller total brain volume than TD males, but there are no differences between ASD and TD females. Total brain volume increases linearly with age in TD group, but it follows a non-linear quadratic trajectory in ASD group with a peak at 12 years of age. Gray matter volume is larger in ASD group than in TD group. White matter volume is larger in ASD males compared to TD males, but smaller in ASD females compared to TD females. ASD group has smaller hippocampi, thalamus, and globus pallidus volumes compared to TD group. ASD has significantly larger Crus I relative volume than TD group. Mean cortical thickness and surface area do not significantly differ between groups. ASD group has reduced age-related cortical thinning in the left orbitofrontal cortex and left posterior cingulate gyrus. |
| Mizuno et al. (15) | 2019 | Cross-sectional; Rest | Total: 232  ASD: 91  TD: 141 | ASD: 11.4  TD: 11.1 | ASD: 0 (0)  TD: 0 (0) | Japan | DSM-5, AQ, SCQ | ASD group has decreased volume in left postcentral gyrus. |
| Baribeau et al. (16) | 2019 | Cross-sectional; Rest | Total: 191  ASD: 159  TD: 32 | ASD: 11.7 (6.12-18.0)  TD: 10.9 (6.1-17.6) | ASD: 30 (19)  TD : 17 (53) | Canada | Clinical assessment, ADI-R, ADOS, SCQ | ASD group has decreased cortical thickness in left lateral regions and right insula, as well as decreased ventral striatum volume. |
| Ni et al. (17) | 2018 | Cross-sectional; Rest | Total: 146  ASD: 85  TD: 61 | ASD: 12.5 (7-17)  TD: 12.4 (7-17) | ASD: 0 (0)  TD: 0 (0) | China | DSM-IV, ICD-10, ADI-R | There are no significant differences in the absolute total gray matter, white matter, and total brain and intra-cranial volumes among the ASD and TD groups. ASD adolescents have significantly less gray matter in the left anterior prefrontal and left lateral occipital/superior parietal lobes than TD children, but not after controlling for ASD dysregulation. |
| Foster et al. (18) | 2015 | Cross-sectional; Rest | Total: 84  ASD: 38  TD: 46 | ASD: 12.4 (6-17)  TD: 12.6 (7-17) | ASD: 0 (0)  TD: 0 (0) | Canada | DSM-IV-TR, ADOS-2, ADI-R | ASD group has increased gray matter in the frontal lobe, temporal lobe, and some parietal and occipital subcortical areas, and decreased gray matter in the temporoparietal junction. |
| Irimia et al. (19) | 2017 | Cross-sectional; Rest | Total: 193  ASD: 110  TD: 83 | ASD: 12.32 (7-18)  TD:12.76 (8-18) | ASD: 55 (50)  TD: 40 (48) | USA | ADI-R, ADOS | Total intracranial volume does not differ between ASD and TD groups. There is no difference between ASD and TD groups for grey matter volume, grey matter thickness, cortical area and mean curvature. |
| Ni et al. (20) | 2020 | Cross-sectional; Rest | Total: 137  ASD: 90  TD: 67 | ASD: 12.9 (7-17)  TD: 12.3 (7-17) | ASD: 0 (0)  TD: 0 (0) | China | DSM-IV, ICD-10, ADI-R | ASD group has increased gyrification in right middle frontal cortex and right orbitofrontal cortex. There is also increased gyrification over the frontoparietal-dorsal attention network, left somatosensory network, left default mode network, and parts of right salience network. |
| Sharda et al. (21) | 2017 | Cross-sectional; Rest | Total: 96  ASD: 46  TD: 50 | ASD: 12.9 (6-16)  TD: 12.8 (6-16) | ASD: 0 (0)  TD: 0 (0) | Canada | ADOS, ADI-R | ASD group has increased cortical thickness in the left temporal lobe, left angular gyrus, and frontal lobe, and has increased cortical surface area (CSA) in right posterior temporal occipital area and right supplementary motor area. ASD group also has decreased covariance of cortical thickness in frontal temporal ROIs. |
| Mensen et al. (22) | 2017 | Cross-sectional; Rest | Total: 180  ASD: 90  TD: 90 | ASD: 13.8 TD: 13.2 | ASD: 16 (18)  TD: 16 (18) | Netherlands | DSM-IV, ADI-R | ASD group has a ~3% decrease cortical volume compared to the TD group driven by changes in the following regions: fusiform gyrus, inferior parietal gyrus, inferior temporal gyrus, medial temporal gyrus, paracingulate, anterior cingulate cortex, superior frontal gyrus, marginal gyrus, insula, and precuneus. ASD group has a ~5% decrease in surface area. |
| **Longitudinal** | | | | | | | | |
| **Author** | **Year** | **Study Design; Task/Stimulus** | **N (at baseline)** | **Age (at baseline)** | **Sex (at baseline)** | **Region** | **ASD Metric** | **Outcome** |
| Shen et al. (23) | 2022 | Longitudinal: 2^nd^ timepoint after 6 months, 3^rd^ timepoint after 12 months; Sleep | Total: 408  FH: 270  NFH: 109 | FH: (0.5-2)  NFH: (0.5-2) | FH: 105 (38.8)  NFH: 43 (39.4) | USA | ADOS, ADI-R | Infants who develop ASD have typically sized amygdala at 6 months, but then exhibit significantly faster amygdala growth between 6 and 24 months. By 12 months, ASD group has significantly larger amygdala volumes than any other group. Amygdala growth rate between 6-12 months is significantly associated with deficits at 24 months |
| Wolff et al. (24) | 2015 | Longitudinal: 2^nd^ timepoint after 6 months, 3^rd^ timepoint after 12 months; Sleep | Total: 378  ASD: 57  TD: 321 | ASD: 0.55 TD: 0.55 | ASD: 10 (18)  TD: 133 (41) | USA | SCQ, ADI-R, RBS-R, ADOS | FH infants that go on to develop ASD have larger corpus callosum area and thickness compared to NFH infants without ASD at 6 months old, particularly in the anterior corpus callosum. Differences in thickness do not persist over time at age 24 months. |
| Shen et al. (25) | 2017 | Longitudinal: 2^nd^ timepoint after 6 months, 3^rd^ timepoint after 12 months; Sleep | Total: 343  ASD: 47  FH: 174  NFH: 122 | ASD: 0.55  FH: 0.55  NFH: 0.57 | ASD: 5 (10.6)  FH: 79 (45.4)  NFH: 46 (37.7) | USA | ADI-R, Family history | Infants who develop ASD have significantly greater extra-axial CSF volume at 6 months compared to both NFH and FH. Extra axial CSF volume is elevated for ASD at all three timepoints, increasing with ASD severity. Extra-axial CSF volume at six months predicts ASD diagnosis with 69% accuracy. |
| Libero et al. (26) | 2019 | Longitudinal: 2^nd^ time point after 1 year; Sleep | Total: 154  ASD: 105  TD: 49 | ASD: 3 (2-3.5) TD: 3 (2-3.5) | ASD: 0 (0) TD: 0 (0) | USA | ADOS-G, ADI-R | ASD group has higher brain volume, reduced gyrification in regions in frontal gyrus, inferior temporal gyrus, paracentral cortex, parahippocampal gyrus, caudal entorhinal cortex, precentral gyrus, and postcentral gyrus. Also, higher surface area in frontal gyrus and cingulate cortex. |
| Reinhardt et al. (27) | 2020 | Longitudinal: Up to two follow up visits, each 1 year apart; Sleep | Total: 310  ASD: 200  TD: 110 | ASD: 3.14  TD: 3.14 | ASD: 63 (31.5) TD: 50 (45.5) | USA | ADOS, ADI-R, DSM-IV | Hippocampus volumes are greater in ASD than TD when adjusting for hemispheric volume. In ASD, difference between right and left hippocampus volumes greater than for TD. |
| Lee et al. (28) | 2021 | Longitudinal: Three subsequent time points, approximately 1 year apart; Asleep at T1-T3, awake at T4 | Total: 384  ASD: 249 TD: 135 | ASD: 3.22  TD: 3.12 | ASD: 95 (38) TD: 61 (45) | USA | ADOS | Boys in ASD group have cerebral overgrowth with a disproportionate megalencephaly subgroup throughout childhood that shows no evidence of volumetric regression or normalization. Girls in ASD group have cerebral volumes similar to girls in TD group, but gray and white matter growth trajectories are slower throughout childhood. |
| Lee et al. (29) | 2022 | Longitudinal: Three subsequent time points, approximately 1 year apart; Asleep at T1-T3, awake at T4 | Total: 414  ASD: 282 TD: 132 | ASD: 3.15  TD: 3.25 | ASD: 93 (33) TD: 61 (46) | USA | ADOS-G or ADOS-2, SRS | ASD and TD children exhibit significant volumetric differences in amygdala-connected networks throughout childhood, and differences in volume increase over time. Autistic individuals have slightly larger total brain volume than TD children at baseline. The amygdala-connected network significantly differs between males and females. ASD males at baseline have larger volumes of bilateral subgenual, anterior cingulate cortex, and right lateral fronto-orbital gyrus than TD males. ASD females have larger volumes of left fusiform gyrus, left superior temporal gyrus, and left entorhinal cortex compared to TD females. |

ABC, Autism Behavior Checklist; ADI, Autism Diagnostic Interview; ADI-R, Autism Diagnostic Interview, Revised; ADOS, Autism Diagnostic Observation Schedule; ADOS-G, Autism Diagnostic Observation Schedule–Generic; ADOS-2, Autism Diagnostic Observation Schedule–Second Edition; CARS, Childhood Autism Rating Scale; DAWBA, Development and Well-Being Assessment; DSM-IV, Diagnostic and Statistical Manual of Mental Disorders, Fourth Edition; DSM-IV-TR, Diagnostic and Statistical Manual of Mental Disorders, Fourth Edition, Text Revision; DSM-5, Diagnostic and Statistical Manual of Mental Disorders, Fifth Edition; FH, family history of autism spectrum disorder; ICD-10, International Classification of Diseases, Tenth Revision; NFH, no family history of autism spectrum disorder; RBS-R, Repetitive Behavior Scales–Revised; SCQ, Social Communication Questionnaire; SRS, Social Responsiveness Scale.

# Supplementary Table 4. DTI Findings.

| **Cross-sectional** | | | | | | | | |
| --- | --- | --- | --- | --- | --- | --- | --- | --- |
| **Author** | **Year** | **Study Design; Task/Stimulus** | **N** | **Age** | **Sex** | **Region** | **ASD Metric** | **Outcome** |
| Solso et al. (30) | 2016 | Cross-sectional; Sleep | Total: 94  ASD: 61  TD: 33 | ASD: 2.5 (1-4)  TD: 2.2 (1-4) | ASD: 13 (21)  TD: 13 (39) | USA | ADOS, VABS | ASD group has higher tract FA and volume in frontal regions than TD before age 3. ASD group shows flatter slope of age-related change in corpus collosum and frontal tracts. No significant difference for posterior tracts in TD and ASD toddlers. |
| Xiao et al. (3) | 2014 | Cross-sectional; Sedation | Total: 78  ASD: 50  TD: 28 | ASD: 2.5 (0-8) TD: 2.35 (0-8) | ASD: 8 (16)  TD: 6 (21.4) | China | DSM-IV-TR, CARS, ADI | ASD toddlers have increased FA in the corpus callosum, posterior cingulate cortex, and limbic lobe. ASD toddlers also have decreased MD in the left corpus callosum, posterior cingulate, limbic lobe, and insular cortex. |
| Andrews et al. (31) | 2019 | Cross-sectional; Sleep | Total: 181  ASD: 127  TD: 54 | ASD: 3.23 (2.16-4.13)  TD: 3.03 (2.09-4.11) | ASD: 42 (33)  TD:26 (48) | USA | DSM-IV, ADOS, ADI-R | ASD children have significantly increased FA in the corpus callosum, inferior frontal-occipital fasciculi, inferior longitudinal fasciculus, superior longitudinal fasciculus, middle cerebellar peduncles, superior cerebellar peduncles, and the corticospinal tract. Females with ASD have increased AD compared to TD females in the corpus callosum, while males with ASD have decreased AD in the corpus callosum. |
| Yu et al. (32) | 2020 | Cross-sectional; Sedation | Total: 149  ASD: 31  TD: 118 | ASD: 4.11 (2-7)  TD: 3.36 (0-8) | ASD: 0 (0)  TD: 66 (56) | China | ADI-R, CARS, CABS, DSM-IV-TR | There is higher residual variance of white matter microstructure in ASD compared to white matter maturational curves in TD children, specifically in commissural and brainstem regions. |
| Aoki et al. (33) | 2017 | Cross-sectional; Rest | Total: 109  ASD: 59  TD: 50 | ASD: 8.9 (1.7)  TD: 9.4 (1.5) | ASD:8 (11)  TD: 38 (73) | USA | DSM-IV-TR, ADOS, ADI-R | ASD children show significantly lower FA, higher MD, higher RD, higher AD in the corpus callosum. |
| Li et al. (34) | 2017 | Cross-sectional; Rest | Total: 73  ASD: 34  TD: 39 | ASD: 9.27 (6-16)  TD: 10.05 (6-16) | ASD: 4 (12)  TD: 10 (26) | China | DMS-V, ADI-R | ASD has significantly increased FA in the right cingulate fasiculus. FA values for cingulate fasiculus are positively correlated with age in ASD group, but not the TD group. |
| Duerden et al. (13) | 2014 | Cross-sectional; Rest | Total: 63  ASD: 33  TD: 30 | ASD: 10.6 (7-15)  TD: 10.7 (7-15) | ASD: 4 (12)  TD: 4 (13) | Canada | ADOS-G, ADI-R | There are no differences in mean FA, MD, AD and RD between ASD and TD groups. |
| Roberts et al. (35) | 2013 | Cross-sectional; Rest | Total: 92  ASD: 53  TD: 39 | ASD: 10.42 TD: 11.02 | Sex/gender not reported | USA | DSM-IV, ADOS, SCQ, SRS | Compared to TD sample, FA did not increase with age but showed increased AD and RD at time of measurement. |
| Roberts et al. (36) | 2014 | Cross-sectional; Rest | Total: 59  ASD: 34  TD: 25 | ASD: 11.47  TD: 11.42 | ASD: 4 (12) TD: 11 (44) | USA | ADOS, SRS, SCQ | ASD group has higher axial diffusivity and mean diffusivity but not radial diffusivity in the left arcuate fasciculus compared to TD group. There are no group differences for AD, MD, RD, or FA in the right arcuate fasciculus. |
| Roberts et al. (37) | 2020 | Cross-sectional; Rest | Total: 117  ASD: 77 TD: 40 | ASD: 11.4 (7-17) TD: 11.5 (8-17) | ASD: 10 (13) TD: 4 (10) | USA | Previous diagnosis with DSM-IV or DSM-5, ADOS or ADOS-2, SCQ, SRS-2, ASRS | ASD group has decreased FA in Heschl’s gyrus. |
| Irimia et al. (19) | 2017 | Cross-sectional; Rest | Total: 193  ASD: 110  TD: 83 | ASD: 12.32 (7-18)  TD 12.76 (8-18) | ASD: 55 (50)  TD: 40 (48) | USA | ADI-R, ADOS | There is a significant bilateral difference for white matter connectivity density based on a sex-by-diagnosis interaction, in the lateral aspect of the temporal lobe, the temporo-parieto-occipital junction and the medial parietal lobe. |
| Carper et al. (38) | 2015 | Cross-sectional; Rest | Total: 80  ASD: 44  TD: 36 | ASD: 13.2  TD: 12.8 | ASD: 8 (18)  TD: 9 (25) | USA | DSM-IV-TR, ADOS, ADI-R | ASD children have increased RD and MD in the corticospinal tract compared to TD children. |
| Solders et al. (39) | 2017 | Cross-sectional; Rest | Total: 67  ASD: 57  TD: 50 | ASD: 13.28 (7-17)  TD: 12.75 (7-17) | ASD: 13 (23)  TD: 12 (24) | USA | ADI-R, ADOS, DSM-5 | FA is reduced in ASD compared to TD group throughout right hemisphere. |
| Hau et al. (40) | 2019 | Cross-sectional; Rest | Total: 115  ASD: 61  TD: 54 | ASD: 13.3 (7.4-18.0)  TD: 13.1 (6.9-17.7) | ASD: 13 (21)  TD: 9 (17) | USA | DSM-5, ADOS, ADI-R | ASD children have higher MD and RD in the left cingulum compared to TD children. ASD group has atypical age-related decline in right posterior cingulate u-fiber volume, which is not present in TD group. |
| Nair et al. (41) | 2015 | Cross-sectional; Rest | Total: 142  ASD: 71  TD: 71 | ASD: 13.8 (7-17)  TD:13.0 (7-17) | ASD: 8 (11)  TD: 16 (23) | USA | ADOS, ADI-R | ASD group has increased MD in the thalamus, paracingulate gyrus, supplementary motor area, anterior cingulate cortex, superior frontal gyrus, temporal lobe, and right superior parietal lobe. ASD group also has decreased FA in ASD in the thalamus, anterior cingulate cortex, and supplementary motor area. |
| Fishman et al. (42) | 2015 | Cross-sectional; Rest | Total: 70  ASD: 35  TD: 35 | ASD:13.7  (9.2-17.7)  TD: 13.2  (8.7-17.6) | ASD: 3 (8)  TD: 7 (20) | USA | DSM-IV-TR, ADOS, ADI-R | ASD group has reduced FA and elevated MD in bilateral tracts connecting inferior frontal gyrus and lateral dorsal posterior medical cortex, compared to TD group. |
| Carper et al. (43) | 2016 | Cross-sectional; Rest | Total: 85  ASD: 41  TD: 44 | ASD: 13.68 (7.4-18.0)  TD: 13.43 (8.0-17.7) | ASD: 9 (22)  TD: 9 (20) | USA | DSM-IV-TR, ADOS, ADI-R | ASD group overall has reduced FA rightward asymmetry, but reduced leftward MD asymmetry within occipital lobe and parietal lobe when compared to TD sample. |
| **Longitudinal** | | | | | | | | |
| **Author** | **Year** | **Study Design; Task/Stimulus** | **N (at baseline)** | **Age (at baseline)** | **Sex (at baseline)** | **Region** | **ASD Metric** | **Outcome** |
| Andrews et al. (44) | 2021 | Longitudinal: One or two subsequent time points, 1 year apart; Sleep | Total: 194  ASD: 125  TD: 69 | ASD: 2.0-3.5  TD: 2.0-3.5 | ASD: 40 (32)  TD: 31 (45) | USA | DSM-IV, ADOS, ADI-R | ASD group has slower development of FA in the cingulum bundle, superior longitudinal fasciculus, internal capsule, and splenium compared to TD. |
| Zhang et al. (45) | 2022 | Longitudinal: 2^nd^ time point 4 years later; Rest | Total: 254  ASD: 182  TD: 72 | ASD: 8.08  TD: 9.00 | ASD: 10 (6)  TD: 24 (33.3) | China | DSM-5, ADOS, ADI-R | Children with ASD show lower FA at T1, but no significant difference at T2. Children with ASD show increased MD in the superior longitudinal fasiculus, inferior longitudinal fasiculus, anterior thalamic radiation, corticospinal tract, and cingulus. Children with ASD show increased RD in the superior longitudinal fasiculus, inferior longitudinal fasiculus, anterior thalamic radiation, corticospinal tract, and cingulus. |

AD, axial diffusivity; ADI, Autism Diagnostic Interview; ADI-R, Autism Diagnostic Interview, Revised; ADOS, Autism Diagnostic Observation Schedule; ADOS-G, Autism Diagnostic Observation Schedule–Generic; ADOS-2, Autism Diagnostic Observation Schedule–Second Edition; ASRS, Autism Spectrum Rating Scales; CABS, Clancy Autism Behavior Scale; CARS, Childhood Autism Rating Scale; DSM-IV, Diagnostic and Statistical Manual of Mental Disorders, Fourth Edition; DSM-IV-TR, Diagnostic and Statistical Manual of Mental Disorders, Fourth Edition, Text Revision; DSM-5, Diagnostic and Statistical Manual of Mental Disorders, Fifth Edition; FA, fractional anisotropy; MD, mean diffusivity; RD, radial diffusivity; SCQ, Social Communication Questionnaire; SRS, Social Responsiveness Scale; SRS-2, Social Responsiveness Scale, Second Edition; VABS, Vineland Adaptive Behavior Scales.

# Supplementary Table 5. fMRI Findings.

| **Cross-sectional** | | | | | | | | |
| --- | --- | --- | --- | --- | --- | --- | --- | --- |
| **Author** | **Year** | **Study Design; Task/Stimulus** | **N** | **Age** | **Sex** | **Region** | **ASD Metric** | **Outcome** |
| Wang et al. (46) | 2022 | Cross-sectional; Sleep | Total: 73  ASD: 32 TD: 41 | ASD: 0.52 TD: 0.52 | ASD: 8 (25) TD: 15 (37) | USA | ADI-R | At 24 months ASD shows significantly increased integration, decreased segregation, and decreased small worldness on surface area measures compared to TD. There are no significant differences in cortical thickness between ASD and TD. Surface area nodal betweenness and nodal degree in the left lateral occipital cortex decrease from 6 to 24 months in ASD compared to TD. |
| Zielinski et al. (47) | 2022 | Cross-sectional; Sleep | Total: 244  ASD: 122 TD: 122 | ASD: 3.13 (2-4) TD: 3.13 (2-4) | ASD: 61 (50) TD: 61 (50) | USA | ADOS-G, ADI-R, DSM-IV-TR, SCQ | The salience network and executive control network have decreased volume and structural covariance in the ASD group, whereas the default mode network has increased volume and structural covariance. Additionally, ASD males have greater structural covariance than TD males across all three networks, while ASD females have decreased covariance compared to TD females. |
| Shen et al. (48) | 2016 | Cross-sectional; Sleep | Total: 72  ASD: 43  TD: 29 | ASD: 3.5  TD: 3.5 | ASD: 0 (0)  TD: 0 (0) | USA | ADOS, ADI-R | ASD group has weaker functional connectivity between the amygdala and medial prefrontal cortex, bilateral temporal lobes, striatum, thalamus, cingulate cortex, cerebellum, as well as weaker functional connectivity between the primary visual cortex and the pre/post central gyrus, striatum, and thalamus. |
| Xiao et al. (49) | 2022 | Cross-sectional; Passive speech paradigm during sleep | Total: 71  ASD: 41 TD: 30 | ASD: (2-5)  TD: (2-5) | ASD: 6 (15) TD: 12 (40) | USA | ASD metric not clearly explained | TD group has robust activation in temporal language regions (bilateral superior temporal cortex) during affective speech, ASD group does not. |
| He et al. (50) | 2018 | Cross-sectional; Sedation | Total: 57  ASD: 28 TD: 29 | ASD: 4.99 (3-7) TD: 4.99 (3-6) | ASD: 0 (0) TD: 0 (0) | China | DSM-IV-TR, ADOS, ADI-R, SRS | ASD group has decreased dynamic functional connectivity variance between the posterior cingulate cortex and the right precentral gyrus. |
| He et al. (51) | 2021 | Cross-sectional; TD group watched videos (rest), ASD group sedated | Total: 78  ASD: 40 TD: 38 | ASD: 5.25 (3.6-7.6) TD: 5.64 (2.7-6.8) | ASD: 8 (20) TD: 10 (26) | China, Spain | DSM-5, ADOS | ASD group has decreased small worldness. |
| Shi et al. (52) | 2013 | Cross-sectional; Rest | Total: 100 ASD: 49  TD: 51 | ASD: 9.6 (6-15) TD: 9.7 (6-15) | ASD: 9 (18)  TD: 12 (24) | USA | ADI-R, ADOS | ASD group has decreased modularity (short-range connectivity) overall and increased short-range connectivity within limbic areas, frontal, and temporal lobes. ASD group also has increased intra-connectedness in the frontal lobe, but decreased connectivity between the frontal lobe and other regions. |
| Floris et al. (53) | 2016 | Cross-sectional; Rest | Total: 118  ASD: 42 TD: 76 | ASD: 10.18 (8.01-12.99) TD: 10.16 (8.07-12.58) | ASD: 8 (19) TD: 22 (29) | UK, USA | DSM-IV, ADOS-G, ADI-R | ASD group has more rightward lateralization in the motor circuit, driven by both right hyperconnectivity and left hypoconnectivity. |
| Di Martino et al. (54) | 2013 | Cross-sectional; Rest | Total: 106  ASD: 56 TD: 50 | ASD: 10.1 (7-13) TD: 10.7 (7-13) | ASD: 7 (13) TD: 13 (26) | USA | DSM-IV-TR, ADOS | ASD group has increased degree centrality in limbic areas, specifically the amygdala, hippocampus, and planum temporale. |
| Dajani et al. (55) | 2019 | Cross-sectional; Rest | Total: 86  ASD: 43 TD: 43 | ASD: 10.37 (8-13) TD: 10.50 (8-13) | ASD: 9 (21) TD: 12 (28) | USA | Previous diagnosis, ADOS-G or ADOS-2, ADI-R | No effect of group on within or between network connectivity differences, even when groups are broken down by comorbidity. DSM-5 classifications do not demonstrate distinct functional connectivity patterns. |
| Supekar et al. (56) | 2021 | Cross-sectional; Rest | Total: 96  ASD: 48  TD: 48 | ASD: 10.9  TD: 10.9 | ASD: 7 (15)  TD: 7 (15) | USA | ADI-R | ASD group has decreased functional connectivity between the salience network and central executive network, as well as the salience network and default mode network. |
| Gooskens et al. (57) | 2019 | Cross-sectional; Stop-signal task | Total: 79  ASD: 26 TD: 53 | ASD: 11.33 (8-12) TD: 10.76 (8-12) | ASD: 9 (35) TD: 24 (45) | Netherlands, UK, Germany, Switzerland | DSM-IV, ADI-R | No difference in brain activation between groups during stop signal task. |
| McNaughton et al. (58) | 2023 | Cross-sectional; Peer interactive chat task | Total: 114  ASD: 43  TD: 71 | ASD: 11.84 (7-15) TD: 11.63 (7-15) | ASD: 7 (16) TD: 28 (39) | USA | ADOS | ASD group has increased task-related activation in the cerebellum. |
| Kilroy et al. (59) | 2021 | Cross-sectional; Action Observation Task, Action Execution Task, Imitation Task, Mentalizing Task | Total: 66  ASD: 30 TD: 33 | ASD: 11.96 (8-17) TD: 12.02 (8-17) | ASD: 7 (23) TD: 11 (33) | USA | ADOS, ADI-R | ASD group has decreased activity compared to TD in the right inferior frontal gyrus during the observation task. ASD group has decreased activity compared to TD in the right superior frontal gyrus/supplementary motor area and right inferior frontal gyrus during the emotional faces imitation task. ASD group has decreased activation compared to TD in the dorsomedial prefrontal cortex, the left inferior frontal gyrus, tringularius, orbitalis, and the left middle frontal gyrus/premotor cortex during the mentalizing task. |
| Butera et al. (60) | 2023 | Cross-sectional; Watching emotional and non-emotional videos of faces | Total: 65  ASD: 28  TD: 37 | ASD: 12 (8-17)  TD: 12 (8-17) | ASD: 6 (21)  TD: 11 (30) | USA | ADOS, ADI-R | ASD group has increased functional connectivity between the left insula and left prefrontal cortex when viewing non-emotional facial expressions. |
| Bos et al. (61) | 2014 | Cross-sectional; Rest | Total: 56  ASD: 27  TD: 29 | ASD: 11.8 (8-18)  TD: 12.2 (8-18) | ASD: 0 (0)  TD: 0 (0) | Netherlands | ADI-R | There are no group differences in within-network connectivity for ten resting-state networks representing the default mode, frontoparietal, striatal, cingulate gyrus, and cerebellar-thalamic networks. However, right insula within-network connectivity increases with age in ASD group and decreases in age for TD group. The ASD group also has reduced connectivity between the default mode network and right superior frontal gyrus/paracingulate, as well as reduced connectivity between striatal network and left middle temporal gyrus. |
| Yerys et al. (62) | 2017 | Cross-sectional; Rest | Total: 163  ASD: 81 TD: 82 | ASD: 12.4 (6-17) TD: 12.4 (6-17) | ASD: 17 (21) TD: 15 (18) | USA | DSM-IV | ASD group has lower whole brain absolute mean connectivity strength than TD group. ASD group has higher within-system relative functional connectivity than TD in ventral attention and retrosplenial-temporal systems. ASD group has lower cross-system functional connectivity between the ventral attention and somatomotor-mouth systems. |
| Yerys et al. (63) | 2019 | Cross-sectional; Rest | Total: 159  ASD: 77 TD: 82 | ASD: 12.4 (6-17) TD: 12.4 (6-17) | ASD: 17 (22) TD: 15 (18) | USA | DSM-IV | ASD group has weaker functional connectivity in the frontoparietal network and within salience ventral attention subnetworks. |
| Lin et al. (64) | 2020 | Cross-sectional; Rest | Total: 117  ASD: 62  TD: 55 | ASD: 12.29 (7-17)  TD: 12.56 (7-17) | ASD: 0 (0)  TD: 0 (0) | Taiwan | DSM-IV-TR, ADI-R | Resting state functional connectivity patterns differ between ASD and TD groups in multiple regions: anterior cerebellum (somatomotor network) connectivity is lower in ASD; bilateral pre-post central gyri (somatomotor network) connectivity is lower in ASD; dorsal anterior cingulate cortex connectivity (salience, ventral attention network) is higher in ASD; thalamus (salience, ventral attention) connectivity is higher in ASD; left inferior frontal gyrus (fronto-parietal network) connectivity is higher in ASD. |
| Doyle-Thomas et al. (65) | 2015 | Cross-sectional; Rest | Total: 95  ASD: 58 TD: 37 | ASD: 12.5 (7-18) TD: 12.7 (7-18) | ASD: 0 (0) TD: 0 (0) | Canada | DSM-IV, ADOS-G, ADI-R | ASD group has decreased connectivity between the posterior cingulate cortex (PCC) and the left middle frontal gyrus, angular gyrus, and inferior frontal gyrus compared to TD group. ASD group has increased connectivity between the PCC and the inferior parietal lobule, superior frontal gyrus, precentral gyrus, right middle frontal gyrus, and superior parietal lobule. PCC and medial prefrontal cortex connectivity increases with age in TD group, but decreases with age in ASD group. |
| Fan et al. (66) | 2021 | Cross-sectional; Semantic Judgement Task | Total: 128  ASD: 61 TD: 67 | ASD child: 10.5 (8-12) ASD adolescent: 14.9 (13-17) TD child: 10.4 (8-12) TD adolescent: 15.1 (13-17) | ASD: 4 (7) TD: 11 (16) | Taiwan, USA | DSM-5, ADI-R | Child and adolescent ASD groups have decreased connectivity between the cuneus, inferior frontal gyrus, and middle temporal gyrus. |
| Vogan et al. (67) | 2018 | Cross-sectional; Letter Matching Task | Total: 57  ASD: 27 TD: 30 | ASD: 12.56 (9-16) TD: 12.96 (9-16) | ASD: 5 (19) TD: 8 (27) | Canada | ADOS | TD children have significantly stronger positive linear relationships between activation (i.e. BOLD-signal) and cognitive load (i.e. task-difficulty) compared to ASD children in the bilateral prefrontal cortex, inferior parietal lobule, and precuneus. |
| Mash et al. (68) | 2020 | Cross-sectional; Rest | Total: 198  ASD: 104  TD: 94 | ASD: 12.6 (7.1-17.1)  TD: 13.0 (7.1-18.0) | ASD: 18 (17) TD: 20 (21) | USA | ADOS | ASD group has increased BOLD power in right temporal regions and widespread overconnectivity between the thalamus and cortex. TD group has positive correlations between EEG alpha power and regional BOLD power; these were weak or negative in ASD group. |
| Green et al. (69) | 2016 | Cross-sectional; Rest, passive auditory and tactile stimuli | Total: 61  ASD: 28 TD: 33 | ASD: 12.95 (7-17) TD: 12.93 (7-17) | ASD: 1 (4) TD: 5 (15) | USA | Previous diagnosis, ADOS-2, ADI-R | ASD and TD groups have similar salience network connectivity. ASD group has increased connectivity between the anterior insula and other brain regions, particularly sensory-motor areas. |
| Zhao et al. (70) | 2022 | Cross-sectional; Rest | Total: 111  ASD: 48 TD: 63 | ASD: 13.0 (10-18) TD: 12.9 (10-18) | ASD: 9 (19) TD: 20 (32) | China | DSM-5 | ASD group has increased regional homogeneity in the bilateral anterior cingulate cortex, left caudate, right posterior cerebellum, and bilateral brainstem. ASD group has decreased regional homogeneity in the left paracingulate, left inferior parietal lobule, bilateral postcentral gyrus, and right anterior cerebellum. |
| Carper et al. (38) | 2015 | Cross-sectional; Rest | Total: 80  ASD: 44  TD: 36 | ASD: 13.2  TD: 12.8 | ASD: 8 (18)  TD: 9 (25) | USA | DSM-IV-TR, ADOS, ADI-R | ASD group has increased functional connectivity between the paracingulate gyrus and inferior prefrontal cortex, middle frontal gyri, inferior parietal lobe, and superior middle gyrus. |
| Nair et al. (41) | 2015 | Cross-sectional; Rest | Total: 142  ASD: 71  TD: 71 | ASD: 13.8 (7-17)  TD:13.0 (7-17) | ASD: 8 (11)  TD: 16 (23) | USA | ADOS, ADI-R | ASD group has overconnectivity in the cingulate gyrus, paracingulate gyrus, auditory cortex, and motor cortex. ASD group also has underconnectivity in the amygdala and occipital lobe. |
| Mash et al. (71) | 2019 | Cross-sectional; Rest | Total: 119  ASD: 62  TD: 57 | ASD: 13.7 (6-18)  TD: 13.1 (6-18) | ASD: 10 (16)  TD: 11 (19) | USA | ADI-R, ADOS-2 | ASD group has overconnectivity in the following functional networks: default mode, executive, frontoparietal, auditory, visual, sensorimotor, cerebellar, and subcortical. |
| Lawrence et al. (72) | 2020 | Cross-sectional; Rest | Total: 169  ASD: 80 TD: 89 | ASD: 13.41 (8-17)  TD: 13.43 (8-17) | ASD: 46 (58) TD: 48 (54) | USA | Clinical assessment, ADOS-2, ADI-R, SRS-2 | The female ASD group has greater functional connectivity between the default mode network and central executive network than the ASD male group. TD males and females only have different connectivity patterns in the salience network. |
| Fishman et al. (42) | 2015 | Cross-sectional; Rest | Total: 70  ASD: 35  TD: 35 | ASD:13.7  (9.2-17.7)  TD: 13.2  (8.7-17.6) | ASD: 3 (8)  TD: 7 (20) | USA | DSM-IV-TR, ADOS, ADI-R | ASD group has decreased within-network connectivity in regions belonging to the imitation network (primary somatosensory cortex/intraparietal sulcus, premotor cortex, inferior parietal lobe, lateral occipital complex, and fusiform gyrus) compared to TD group. There is significant overconnectivity in ASD between imitation nodes and extraneous regions (precuneus and posterior cingulate, angular gyrus, frontal eye fields, superior orbital gyrus, and frontopolar prefrontal cortex). |
| Abbott et al. (73) | 2016 | Cross-sectional; Rest | Total: 75  ASD: 37  TD: 38 | ASD: 13.9 (8-17)  TD: 13.0 (8-17) | ASD: 5 (14)  TD: 8 (21) | USA | ADOS, ADI-R, DSM-5 | ASD group has overconnectivity between the default mode network, salience network, and central executive network. |
| Fishman et al. (74) | 2018 | Cross-sectional; Rest | Total: 105  ASD: 55 TD: 50 | ASD: 13.7 (7.4-17.8) TD: 13.4 (8.0-17.6) | ASD: 9 (16) TD: 9 (18) | USA | DSM-5, ADOS-2, ADI-R | ASD group has decreased connectivity between the amygdala and inferior occipital gyrus, and increased connectivity between the amygdala and primary motor cortex/primary somatosensory area. The age-related increases in amygdala connectivity seen in TD group are absent or reversed in ASD group. |
| Jao Keehn et al. (75) | 2021 | Cross-sectional; Rest | Total: 93  ASD: 50 TD: 43 | ASD: 13.5 (7.4-18.0) TD: 13.6 (8.1-17.7) | ASD: 8 (16) TD: 5 (12) | USA | DSM-5, ADI-R, ADOS | ASD group has decreased connectivity between the visual cortices and the anterior insula. |
| Keown et al. (76) | 2013 | Cross-sectional; Rest | Total: 58  ASD: 29 TD: 29 | ASD: 13.8  TD: 13.5 | ASD: 4 (14) TD: 7 (24) | USA | ADOS | ASD group has strong local connectivity in posterior cingulate cortex, precuneus, medial prefrontal cortex, and primary visual cortex. ASD group has low connectivity in temporal lobe (except posterior regions). |
| Gao et al. (77) | 2019 | Cross-sectional; Rest | Total: 102  ASD: 52 TD: 50 | ASD: 13.7 (9.2-18.0) TD: 13.6 (8.0-17.6) | ASD: 8 (15) TD: 7 (14) | USA | DSM-5, ADI-R, ADOS or ADOS-2 | ASD group has increased/atypical within-network connectivity in language regions (inferior parietal lobule, angular gyrus, and precuneus/posterior cingulate cortex, between bilateral dorsal precuneus and left supramarginal gyrus, and between right inferior parietal lobule and the left pericentral region). ASD group also has increased connectivity between language regions and both the posterior cingulate cortex and middle occipital and lingual gyri. |
| Jung et al. (78) | 2021 | Cross-sectional; Mild aversive auditory and tactile stimuli | Total: 79  ASD: 49 TD: 30 | ASD: 13.78 (8-18) TD: 13.65 (8-18) | ASD: 12 (24) TD: 9 (30) | USA | Clinical assessment | ASD group has increased activity in the middle frontal gyrus, inferior frontal gyrus, frontal pole, prefrontal cortex, temporal regions, and occipital cortex associated with aversive stimuli. TD group demonstrates the opposite response to aversive stimuli. |
| Khan et al. (79) | 2015 | Cross-sectional; Rest | Total: 56  ASD: 28 TD: 28 | ASD: 14.27  TD: 13.3 | ASD: 4 (14) TD: 7 (25) | USA | ADOS, ADI-R, SRS, SCQ | ASD group has cerebral-cerebellar (superior temporal cortex, primary sensory cortex, premotor cortex, and occipital cortex) functional overconnectivity. |
| Wood et al. (80) | 2021 | Cross-sectional; Rest | Total: 70  ASD: 35 TD: 35 | ASD: 14.57 (8-17)  TD: 13.09 (8-17) | ASD: 9 (26) TD: 12 (34) | USA | ADI-R, ADOS, Clinical assessment | Higher thalamic GABA/Cr is correlated more strongly with lower functional connectivity between the thalamus and the somatosensory cortex, occipital cortex, anterior prefrontal cortex, and cerebellum in ASD group compared to TD group. |
| Linke et al. (81) | 2018 | Cross-sectional; Rest | Total: 78  ASD: 40 TD: 38 | ASD: 14.02 (9-18)  TD: 13.66 (8-17) | ASD: 8 (20)  TD: 6 (16) | USA | DSM-5, ADOS, ADI-R | ASD group has increased connectivity between the auditory cortex and the thalamus. Connectivity between left and right auditory cortices is positively correlated with thalamic connectivity in the ASD group. |
| Solomon et al. (82) | 2014 | Cross-sectional; Preparing to Overcome Potency Task | Total: 54  ASD: 27 TD: 27 | ASD: 15.4 (12-18)  TD: 16.0 (12-18) | ASD: 5 (19)  TD: 5 (19) | USA | ADOS | Between early and late adolescence, the ASD group shows an increase in functional connectivity between the ventrolateral prefrontal cortex and anterior cingulate cortex, while the TD group shows a decrease. Older ASD group has decreased parietal lobe activation compared to TD during cognitive control task. |
| Di Martino et al. (83) | 2014 | Cross-sectional; Rest | Total: 1112  ASD: 539  TD: 573 | ASD: 17.0 (7.0-64.0)  TD: 17.0 (7.0-64.0) | ASD: 65 (12)  TD: 99 (17) | USA | Clinical assessment, ADOS, ADI-R | ASD group has overall decreased functional connectivity, with some hyperconnectivity in subcortical regions. ASD group also has atypical interhemispheric connectivity. |
| **Longitudinal** | | | | | | | | |
| **Author** | **Year** | **Study Design; Task/Stimulus** | **N (at baseline)** | **Age (at baseline)** | **Sex (at baseline)** | **Region** | **ASD Metric** | **Outcome** |
| Liu et al. (84) | 2020 | Longitudinal: 2^nd^ time point at 9 months of age; Sleep | Total: 125  FH: 71  NFH: 54 | ASD: 1.5 months  TD: 1.5 months | FH: 29 (41)  NFH: 26 (48) | USA | ASD metric not clearly explained | At 1.5 months, FH infants have lower interhemispheric connectivity between left Heschl's gyrus and right sensorimotor regions compared to NFH infants. At 9 months, FH infants show increased connectivity between left Heschl's gyrus, left postcentral gyrus, and parietal regions. 9-month old FH infants also have decreased connectivity between right auditory cortex and frontal regions, but increased connctivity between thalamus and right superior temporal gyrus. FH infants do not show significant changes in connectivity between 1.5 and 9 months, whereas NFH infants show increases and decreased in connectivity between regions listed here. |
| Linke et al. (85) | 2023 | Longitudinal: 2^nd^ time point approximately 3 years after baseline; Sleep | Total: 116  ASD: 70  TD: 46 | ASD: 9.6 months  TD: 9.9 months | ASD: 16 (23)  TD: 21 (46) | USA | DSM-IV-TR, ADI-R, SCQ | ASD group has increased functional connectivity between the thalamus and Heschl’s gyrus. ASD group also has increased BOLD signal in the auditory cortex suring natural sleep. |

ADI-R, Autism Diagnostic Interview, Revised; ADOS, Autism Diagnostic Observation Schedule; ADOS-G, Autism Diagnostic Observation Schedule–Generic; ADOS-2, Autism Diagnostic Observation Schedule–Second Edition; BOLD, blood oxygen level dependent; DSM-IV, Diagnostic and Statistical Manual of Mental Disorders, Fourth Edition; DSM-IV-TR, Diagnostic and Statistical Manual of Mental Disorders, Fourth Edition, Text Revision; DSM-5, Diagnostic and Statistical Manual of Mental Disorders, Fifth Edition; FH, family history of autism spectrum disorder; GABA/Cr, gamma-aminobutyric acid to creatine ratio; NFH, no family history of autism spectrum disorder; SCQ, Social Communication Questionnaire; SRS, Social Responsiveness Scale.

# Supplementary Table 6. MRS Findings.

| **Cross-sectional** | | | | | | | | |
| --- | --- | --- | --- | --- | --- | --- | --- | --- |
| **Author** | **Year** | **Study Design; Task/Stimulus** | **N** | **Age** | **Sex** | **Region** | **ASD Metric** | **Outcome** |
| Mori et al. (86) | 2013 | Cross-sectional; Sedation | Total: 108  ASD: 77 TD: 31 | ASD: 4.1 (3-6) TD: 4.0 (3-6) | ASD: 20 (26) TD: 8 (26) | Japan | DSM-IV-TR | ASD group has decreased NAA in the amygdala and orbitofrontal cortex. No effect of group on Cho or Cr concentrations. |
| Goji et al. (87) | 2017 | Cross-sectional; Sedation | Total: 53  ASD: 34 TD: 19 | ASD: 5.12 (2-12) TD: 5.6 (2-11) | ASD: 6 (18) TD: 7 (37) | Japan | DSM-IV-TR | Children with Asperger’s have decreased NAA, Cr, Cho, and mI in the anterior cingulate cortex but not the cerebellum. |
| Ito et al. (88) | 2017 | Cross-sectional; Sedation | Total: 177  ASD: 112 TD: 65 | ASD: 6.4 (4-14)  TD: 6.7 (2-15) | ASD: 20 (18) TD: 25 (38) | Japan | DSM-IV-TR | ASD group has decreased GABA/Cr in the anterior cingulate cortex and cerebellum, and increased Glu/Cr in the cerebellum. |
| DeMayo et al. (89) | 2021 | Cross-sectional; Watching video (rest) | Total: 59  ASD: 24 TD: 35 | ASD: 8.87 (4-12) TD: 8.46 (4-12) | ASD: 1 (4) TD: 14 (40) | Australia | ADOS-2, SRS-2 | GABA concentration is positively correlated with age in ASD, but not TD, youth. |
| He et al. (90) | 2021 | Cross-sectional; Rest | Total: 106  ASD: 44 TD: 62 | ASD: 10.32 TD: 9.69 | ASD: 2 (5) TD: 16 (26) | USA | ADOS-G, ADI-R, DSM-5 | ASD group has increased Glx in the primary somatosensory cortex, but not the thalamus. |
| Puts et al. (91) | 2017 | Cross-sectional; Watching video (rest) | Total: 72  ASD: 37 TD: 35 | ASD: 10.69 (8-12)  TD: 10.09 (8-12) | ASD: 6 (16) TD: 8 (23) | USA | ADOS-G, ADI-R, DSM-IV | ASD group has decreased GABA in the primary somatosensory cortex, but not the occipital lobe. |
| Naaijen et al. (92) | 2017 | Cross-sectional; Rest | Total: 133  ASD: 51 OCD: 29 TD: 53 | ASD: 10.78 (8-13) TD: 10.45 (8-13) | ASD: 12 (24) TD: 17 (32) | Netherlands, UK, Germany | RBS, ADI-R, DSM-IV-TR; DISC, DWA, or K-SADS | ASD group has increased Glu concentration in the anterior cingulate cortex. |
| Naaijen et al. (93) | 2018 | Cross-sectional; Rest | Total: 141  ASD: 54 OCD: 31 TD: 56 | ASD: 10.72 (8-13)  TD: 10.44 (8-13) | ASD: 11 (20) TD: 18 (32) | Netherlands, UK, Germany | RBS, ADI-R; DISC, DAWBA, or K-SADS | ASD group has decreased NAA in the striatum, which is positively correlated with increased striatal volume. |
| Roberts et al. (37) | 2020 | Cross-sectional; Rest | Total: 117  ASD: 77 TD: 40 | ASD: 11.4 (7-17) TD: 11.5 (8-17) | ASD: 10 (13) TD: 4 (10) | USA | Previous diagnosis with DSM-IV or DSM-5, ADOS or ADOS-2, SCQ, SRS-2, ASRS | ASD group has decreased GABA in the left hemisphere, but there is no difference in the right hemisphere. |
| Edmondson et al. (94) | 2020 | Cross-sectional; Watching video (rest) | Total: 41  ASD: 21 TD: 20 | ASD: 11.8 (9.8-14.5) TD: 11.3 (9.0-15.0) | ASD: 4 (19) TD: 4 (20) | USA | ADOS-2, SCQ, DSM-5 | No effect of group on GABA levels in the frontal eye fields, temporal-parietal junction (TPJ), or visual cortex. ASD group has decreased NAA, Cr, and Glu in the TPJ. |
| Hardan et al. (95) | 2016 | Cross-sectional; Rest | Total: 34  ASD: 17 TD: 17 | ASD: 12.5 (8-15) TD: 11.6 (8-15) | ASD: 0 (0) TD: 0 (0) | USA | ADI-R, ADOS, Clinical assessment, SRS | ASD group has decreased NAA in the anterior frontal white matter and anterior middle centrum semiovale. |
| Doyle-Thomas et al. (96) | 2014 | Cross-sectional; Rest | Total: 36  ASD: 20 TD: 16 | ASD: 11.5 (7-18) TD: 12.9 (7-18) | ASD: 5 (25) TD: 8 (50) | Canada, USA | DSM-IV, ADOS ADI-R | ASD group has higher Glx/Cr concentration in the putamen. No difference in metabolite concentrations between groups in the caudate or thalamus. |
| Carvalho Pereira et al. (97) | 2018 | Cross-sectional; Rest | Total: 34  ASD: 20 TD: 14 | ASD: 13 (11-18) TD: 13 (10-18) | Sex/gender not reported | UK, Portugal | ADI-R, ADOS, DSM-5, SCQ, SRS | ASD group has decreased NAA, Cr, and Glx/NAA in the medial prefrontal cortex. |
| Rojas et al. (98) | 2014 | Cross-sectional; Watching video (rest) | Total: 32  ASD: 17 SIB: 14 TD: 17 | ASD: 14.01 TD: 12.44 | ASD: 3 (18) TD: 9 (53) | USA | ADOS, ADI-R or SCQ, DSM-IV, SRS, BAPQ | ASD group has a decreased GABA/Cr ratio in the perisylvian region. |
| Wood et al. (80) | 2021 | Cross-sectional; Rest; Rest | Total: 70  ASD: 35 TD: 35 | ASD: 14.57 (8-17)  TD: 13.09 (8-17) | ASD: 9 (26) TD: 12 (34) | USA | ADI-R, ADOS, Clinical assessment | No group difference in GABA/Cr, Glx/Cr, or GABA/Glx in the thalamus or somatosensory cortex. |
| Joshi et al. (99) | 2013 | Cross-sectional; Rest | Total: 14  ASD: 7 TD: 7 | ASD: 14 (12-17) TD: 14 (12-17) | ASD: 0 (0) TD: 0 (0) | USA | DSM-IV, SRS | ASD group has increased Glu in the anterior cingulate cortex. |
| Cochran et al. (100) | 2015 | Cross-sectional; Rest | Total: 27  ASD: 13  TD: 14 | ASD: 14.9 (13.1-17.5) TD: 14.7 (13.1-17.4) | ASD: 0 (0) TD: 0 (0) | USA | DSM-IV, ADOS, SRS | ASD group has increased Gln, but there is no group difference in Glu, GABA, NAA, mI, Cho or Cr levels in the anterior cingulate cortex. |
| Drenthen et al. (101) | 2016 | Cross-sectional; Rest | Total: 32  ASD: 15 TD: 17 | ASD: 16.2 (14-18) TD: 15.3 (12-17) | ASD: 1 (7) TD: 1 (6) | Netherlands, USA | DSM-IV, ADOS | ASD group has increased Glu/Cr and decreased GABA/Glu in the occipital lobe. No group difference in Cr levels. |
| **Longitudinal** | | | | | | | | |
| **Author** | **Year** | **Study Design; Task/Stimulus** | **N (at baseline)** | **Age (at baseline)** | **Sex (at baseline)** | **Region** | **ASD Metric** | **Outcome** |
| Corrigan et al. (102) | 2013 | Longitudinal: Two subsequent time points, 3 years apart; Sedation | Total: 68  ASD: 45 DD: 13 TD: 10 | ASD: 4.0 (3-4) TD: 3.8 (3-4) | ASD: 7 (16) TD: 2 (20) | USA | ADI-R, ADOS-G, DSM-IV-TR | ASD and DD groups have lower NAA, Cho, Cr, and Glx in the basal ganglia and anterior commissure across ages 3-10. |
| Hollestein et al. (103) | 2021 | Longitudinal: 2^nd^ time point after 1 year; Rest | Total: 74  ASD: 24 OCD: 15 TD: 35 | ASD: 11.38 (8-16)  TD: 10.70 (8-16) | ASD: 7 (29) TD: 14 (40) | Netherlands, Germany, Switzerland, UK | ADI-R, DSM-IV | Glu in the anterior cingulate cortex decreases more between time points in ASD group compared to TD group. |

ADI-R, Autism Diagnostic Interview, Revised; ADOS, Autism Diagnostic Observation Schedule; ADOS-G, Autism Diagnostic Observation Schedule–Generic; ADOS-2, Autism Diagnostic Observation Schedule–Second Edition; ASRS; Autism Spectrum Rating Scale; BABQ, Broad Autism Phenotype Questionnaire; Cho, choline-containing compounds; Cr, creatine/phosphocreatine; DAWBA, Developmental and Well-Being Assessment; DD, developmentally disabled; DISC, Diagnostic Interview Schedule for Children; DSM-IV, Diagnostic and Statistical Manual of Mental Disorders, Fourth Edition; DSM-IV-TR, Diagnostic and Statistical Manual of Mental Disorders, Fourth Edition, Text Revision; DSM-5, Diagnostic and Statistical Manual of Mental Disorders, Fifth Edition; GABA, gamma-aminobutyric acid; GABA/Cr, gamma-aminobutyric acid to creatine ratio; GABA/Glu, gamma-aminobutyric acid to glutamate ratio; GABA/Glx, gamma-aminobutyric acid to combined glutamate/glutamine signal ratio; Glu, glutamate; Glu/Cr, glutamate to creatine ratio; Glx, combined glutamate/glutamine signal; Glx/Cr, combined glutamate/glutamine signal to creatine ratio; mI, myo-inositol; NAA, n-acetylaspartate; RBS, Repetitive Behavior Scale; SCQ, Social Communication Questionnaire; SRS, Social Responsiveness Scale; SRS-2, Social Responsiveness Scale, Second Edition.

# Supplementary Table 7. fNIRS Findings.

| **Cross-sectional** | | | | | | | | |
| --- | --- | --- | --- | --- | --- | --- | --- | --- |
| **Author** | **Year** | **Study Design; Task/Stimulus** | **N** | **Age** | **Sex** | **Region** | **ASD Metric** | **Outcome** |
| Edwards et al. (104) | 2017 | Cross-sectional; Passive auditory stimuli | Total: 38 FH: 21  NFH: 17 | FH: 3.62 months  NFH: 3.58 months | FH: 8 (38)  NFH: 7 (41) | USA | ASD metric not clearly explained | FH female infants show significantly higher oxy-HB responses to auditory stimuli compared to NFH females, FH males, and NFH males in the left anterior region, but not in other regions. |
| Braukmann et al. (105) | 2018 | Cross-sectional; Social and non-social videos and images | Total: 29 FH: 16  NFH: 13 | FH: 5.37 (4.70-6.51) months  NFH: 5.27 (4.63-6.01) months | FH: 9 (56)  NFH: 4 (31) | Netherlands | ASD metric not clearly explained | FH participants showed increased oxy-Hb for non-social dynamic condition in channel 22 (right superior temoral sulcus-temporoparietal junction).  No significant activation in ox-Hb for social condition or deoxy-Hb for social and non-social. |
| Bhat et al. (106) | 2019 | Cross-sectional; Social Interaction Paradigm | Total: 15  FH: 9  NFH: 6 | FH: 7.0 (6-9) months NFH: 7.5 (6-9) months | FH: 2 (22) NFH: 5 (83) | USA | Social Interaction Paradigm | FH infants have lower right-hemispheric oxy-Hb signals (lower functional activation) than NFH infants in both social and post-social situations. FH infants show lower left-hemispheric deoxy-Hb signals (lower functional activation) than NFH infants during pre-social and social situations. Inter- and intra-hemispheric functional connectivity is greater in FH infants in pre- and post-social situations, both lower in social situations when compared to NFH infants. |
| Hou et al. (107) | 2022 | Cross-sectional; Video clips of humans and robots | Total: 98  ASD: 45 TD: 53 | ASD: 4.89 (4-6) TD: 5.06 (4-6) | ASD: 4 (9) TD: 17 (32) | China | ADOS, CARS | ASD children show significantly lower oxy-Hb in right dorsolateral prefrontal cortex compared to neurotypical controls when "socially interacting" with sequential video clips of a robot.  No significant difference in right dorsolateral prefrontal cortex oxy-Hb between the ASD and neurotypical groups when "socially interacting" with video clips of a human. |
| Kikuchi et al. (108) | 2013 | Cross-sectional; Watching video (rest) | Total: 30  ASD: 15 TD: 15 | ASD: (3.75-6.83)  TD: (3.75-6.83) | ASD: 2 (13) TD: 2 (13) | Japan | ADOS-G, DISCO, DSM-IV | Children with ASD have a greater oxy-Hb connectivity signal in aPFC than TD controls, and the strength of the signal correlated to symptom severity defined by ADOS. |
| Jia et al. (109) | 2018 | Cross-sectional; Watching video (rest) | Total: 24  ASD: 12 TD: 12 | ASD: 6.1 (4-9) TD: Age matched | ASD: 3 (25) TD: 3 (25) | China | DSM-IV, ABC | Children with ASD showed more normalized regional spatial complexity than TD children. Children with ASD also showed more variance in the normalization of regional spatial complexity for the right temporal lobe compared to TD children. |
| Li & Yu (110) | 2016 | Cross-sectional; Watching video (rest) | Total: 24  ASD: 12 TD: 12 | ASD: 6.1 (4.8-8.0) TD: Age matched | ASD: 3 (25) TD: 3 (25) | China | Clinical assessment, ABC | Children with HFA have multiple weak interregional connections (lower oxy-Hb signal) between right and left PFC and bilateral regions of TC, and these weaknesses are predictive of diagnosis. |
| Jia et al. (111) | 2018 | Cross-sectional; Watching video (rest) | Total: 66  ASD: 35 TD: 31 | ASD: 5.96 (4-9)  TD: 6.56 (4-9) | ASD: 12 (34) TD: 11 (35) | China | ABC | The DFA exponent in the ASD group is smaller for oxy-Hb in the left temporal region and smaller for deoxy-Hb in bilateral temporo-occipital regions compared to the TD group. The correlation between age and DFA for oxy-Hb and deoxy-Hb is weaker in the ASD group in frontal, temporal, and occipital regions. |
| Lin et al. (112) | 2022 | Cross-sectional; Rest | Total: 47  ASD: 25 TD: 22 | ASD: 9.3 TD: 9.5 | ASD: 7 (28) TD: 4 (18) | China | DSM-IV | ASD children have weaker coupling between oxy-Hb and dexoy-Hb in each hemisphere compared to TD children, specifically in the temporal cortex. |
| Zhang et al. (113) | 2021 | Cross-sectional; Rest | Total: 47  ASD: 25 TD: 22 | ASD: 9.3 TD: 9.5 | ASD: 7 (28) TD: 4 (18) | China | DSM-IV | Participants with ASD show increased complexity of brain activity as measured by oxy-Hb signals compared to TD participants, especially in the left frontal lobe. |
| Yasumura et al. (114) | 2013 | Cross-sectional; Stroop | Total: 26  ASD: 11  TD: 15 | ASD: 10.51 (10-11) TD: 9.56 (9-11) | ASD: 4 (36) TD: 9 (60) | Japan | DSM-IV | Children with ASD did not significantly differ from TD in color interference, task error, reaction time, or percent correct. Oxy-Hb signals during tasks were only modestly higher in the right hemisphere for ASD children compared to TD and ADHD. |
| Iwanaga et al. (115) | 2013 | Cross-sectional; Mental state task | Total: 32  ASD: 16 TD: 16 | ASD: 11.5 (8-14) TD: 11.4 (8-14) | ASD: 2 (13) TD: 4 (25) | Japan | DSM-IV, PARS, ASSQ-R | ASD children have lower oxy-Hb signal (activation) in the prefrontal cortex than TD children during a mental state task (Theory of Mind), but there is no difference between groups during an object description task. |
| Mori et al. (116) | 2015 | Cross-sectional; Emotional face imitation task | Total: 20  ASD: 10 TD: 10 | ASD: 11.5 (9-14) TD: 11.8 (9-14) | ASD: 0 (0) TD: 0 (0) | Japan | Clinical assessment, DSM-IV-TR | Autistic subjects have a lower concentration of oxygenated hemoglobin in the pars opercularis of the inferior frontal gyrus during facial imitation task. This is significantly elevated after emotional facial expression imitation training. |
| Schudlo et al. (117) | 2021 | Cross-sectional; Tactile temperature task | Total: 32  ASD: 15 TD: 17 | ASD: 12.5 (10-15) TD: 12.9 (10-15) | ASD: 0 (0) TD: 0 (0) | Canada | ADOS-2, ADI-R | ASD group exhibits a lower peak relative oxy-Hb signal in the prefrontal and parietal cortices in response to a noxious cold stimulus (submerging hand in cold water) compared to TD group. |
| Sun et al. (118) | 2023 | Cross-sectional; Rest | Total: 66  ASD: 25 TD: 22 | ASD: 9 TD: 9.5 | ASD: 7 (28)  TD: 4 (18) | China | DSM-IV | Normalized mutual information (a measure of functional connectivity) as measured by oxy-HB signal between bilateral temporal regions is lowest in children with ASD, then in children with typical development, then in young, healthy adults. |
| Yeung et al. (119) | 2019 | Cross-sectional; n-Back task | Total: 36  ASD: 18 HFA: 18 | ASD: 14.11  TD: 14.21 | ASD: 1 (6) TD: 6 (33) | China | ADI-R, DSM-5, SRS-2 | ASD adolescents exhibit a larger mean increase of oxy-Hb in the right prefrontal regions in response to greater working memory load (i.e. rightward lateralization), whereas TD adolescents have oxy-Hb increases in both left and right prefrontal regions. |
| Yeung et al. (120) | 2019 | Cross-sectional; Category fluency test | Total: 36  ASD: 20 TD: 16 | ASD: 14.44 (11-18) TD: 14.27 (11-18) | ASD: 0 (0) TD: 0 (0) | China | DSM-5, ADI-R | ASD individuals exhibit oxy-Hb signal activation in the lateral and medial frontal lobe during an image processing/word production task, as opposed to TD controls that exhibit activation primarily in lateral frontal regions. This effect differed between groups only in the animal word retrieval category. |
| Jung et al. (121) | 2016 | Cross-sectional; Viewing images of faces and robots during n-back task | Total: 20  ASD: 8  TD: 12 | ASD: 15.6 (7-36) TD: 14.5 (7-36) | ASD: 0 (0) TD: 0 (0) | USA | ADOS | When viewing human faces, ASD children have no difference in oxy-Hb signal between left and right hemispheres (specifically temporo-occipital regions), whereas TD group has higher oxy-Hb signal in the right hemisphere than in the left hemisphere (i.e. right hemisphere lateralization). |
| **Longitudinal** | | | | | | | | |
| **Author** | **Year** | **Study Design; Task/Stimulus** | **N (at baseline)** | **Age (at baseline)** | **Sex (at baseline)** | **Region** | **ASD Metric** | **Outcome** |
| Pecukonis et al. (122) | 2020 | Longitudinal: Time points at 3mo, 6mo, 9mo, 12mo, 18mo, 24mo, and 36mo; Listening to speech stimuli during silent video | Total: 31  FH: 17 NFH: 14 | FH: 7 (5-8) months NFH: 7 (5-8) months | FH: (50)  NFH: 7 (50) | USA | One full sibling with ASD, SCQ | While repetitive and random syllable strings elucidate similar brain response patterns in both FH and NFH children, NFH children have the strongest activation in anterior portions of the brain, where FH children have similar activation globally. FH infants later diagnosed with ASD have reduced brain responses in bilateral anterior regions. |
| Zhang et al. (123) | 2022 | Longitudinal: Time points at 5mo and 10mo; Rest | Total: 45  FH: 26  NFH: 19 | FH: 5 months  NFH: 5 months | FH: 15 (58) NFH: 6 (33) | China/  Belgium | One full or half sibling with ASD, | FH infants exhibit increased local and nodal efficiency and connectivity in whole brain compared to NFH controls at 5 months of age. There are no significant differences at 10 months of age. |

DFA: detrended fluctuation analysis

# Supplementary Table 8. MEG Findings.

| **Cross-sectional** | | | | | | | | |
| --- | --- | --- | --- | --- | --- | --- | --- | --- |
| **Auditory Event Related Field** | | | | | | | | |
| **Author** | **Year** | **Study Design; Task/Stimulus** | **N** | **Age** | **Sex** | **Region** | **ASD Metric** | **Outcome** |
| Yoshimura et al. (124) | 2017 | Cross-sectional; Auditory oddball | Total: 93  ASD: 47 TD: 46 | ASD: 5.0 (3.3-6.0)  TD: 4.9 (3.1-6.6) | ASD: 11 (23) TD: 11 (24) | Japan | DISCO, ADOS-G, K-ABC, DSM-IV | ASD group has lower early magnetic mismatch field amplitude in left superior temporal and left transverse temporal gyri compared to TD group. |
| Yoshimura et al.(125) | 2021 | Cross-sectional; Passive auditory stimuli | Total: 75  ASD: 29 TD: 46 | ASD: 6.2 (5.0-8.2) TD: 5.9 (5.3-7.6) | ASD: 8 (28) TD: 5 (11) | Japan | ADOS-G, DISCO, K-ABC, DSM-IV or DSM-5 | ASD group has shorter P1m latency in left hemisphere after pure tone stimulus; no effect of group on P1m intensity. |
| Yoshimura et al. (126) | 2016 | Cross-sectional; Auditory oddball | Total: 70  ASD: 35 TD: 35 | ASD: 6.2 (2.7-10.1) TD: 6.3 (3.2-9.3) | ASD: 8 (23) TD: 8 (23) | Japan | ADOS, DISCO, K-ABC, DSM-IV | TD group has growth of P1m auditory evoked response across development. There is no growth of P1m amplitude in the ASD group, and P1m latency is shorter. |
| Stroganova et al. (127) | 2020 | Cross-sectional; 40 Hz click train | Total: 70  ASD: 35 TD: 35 | ASD: 9.69 (7.2-12.3) TD: 10.08 (7.3-12.9) | ASD: 0 (0) TD: 0 (0) | Russia | DSM-5, SRS, AQ, SCQ | ASD and TD groups have similar auditory steady state response (ASSR) to 40Hz click train, but the sustained field in Heschl's gyrus is reduced in the ASD group. |
| Edgar et al. (128) | 2013 | Cross-sectional; Passive auditory stimuli | Total: 141  ASD: 105 TD: 36 | ASD: 10.07 (6-16) TD; 10.90 (6-16) | ASD: 11 (10) TD: 19 (53) | USA | ADOS, SRS, SCQ | ASD group has delayed M100 latency and decreased M100 amplitude over the superior temporal gyrus. TD group also demonstrated greater inter-trial coherence (ITC, or phase-locking). ASD group also has increased pre-stimulus noise and post-stimulus gamma abnormalities. |
| Roberts et al.(35) | 2013 | Cross-sectional; Passive auditory stimuli | Total: 92  ASD: 53 TD: 39 | ASD: 10.42 TD: 11.02 | Sex/gender not reported | USA | DSM-IV, ADOS, SCQ, SRS | ASD group has increased M50 latency compared to TD group. M50 latency decreases with age in ASD and TD. |
| Roberts et al. (37) | 2020 | Cross-sectional; Passive auditory stimuli | Total: 117  ASD: 77 TD: 40 | ASD: 11.4 (7-17) TD: 11.5 (8-17) | ASD: 10 (13) TD: 4 (10) | USA | Previous diagnosis with DSM-IV or DSM-5, ADOS or ADOS-2, SCQ, SRS-2, ASRS | ASD group has more variation in M50 latency modulation. |
| **Connectivity** | | | | | | | | |
| **Author** | **Year** | **Study Design; Task/Stimulus** | **N** | **Age** | **Sex** | **Region** | **ASD Metric** | **Outcome** |
| Duan et al. (129) | 2017 | Cross-sectional; Watching videos (rest) | Total: 58  ASD: 28 TD: 30 | ASD: 5.5 TD: 5.3 | ASD: 5 (18) TD: 5 (17) | Japan | ADOS, DSM-IV | ASD group has stronger connectivity between temporal and frontal/parietal lobes. TD group has stronger short-range connectivity across the brain. Characteristic path length and clustering coefficients are correlated with age in TD group, but not ASD group. |
| Kikuchi et al. (130) | 2013 | Cross-sectional; Watching video (rest) | Total: 70  ASD: 35 TD: 35 | ASD: 5.5 (3.3-7.8) TD: 5.4 (3.3-7.1) | ASD: 6 (17) TD: 6 (17) | Japan | ADOS, DISCO, K-ABC, DSM-IV | ASD group has a significantly lower lateralization index of the gamma-1 band coherence (parietotemporal network) compared to TD group, indicating atypical rightward-lateralized connectivity via gamma oscillation. |
| Kikuchi et al. (131) | 2015 | Cross-sectional; Watching video (rest) | Total: 100  ASD: 50 TD: 50 | ASD: 5.6 (3.2-7.7) TD: 5.6 (3.0-8.1) | ASD: 11 (22) TD: 11 (22) | Japan | ADOS, DISCO, K-ABC, DSM-IV | ASD group has reduced connectivity between left anterior and right posterior brain areas. |
| Alho et al. (132) | 2023 | Cross-sectional; Sinewave speech stimulus | Total: 55 ASD: 27 TD: 28 | ASD: 14.0 (7-17) TD: 13.6 (7-17) | ASD: 4 (15) TD: 6 (21) | USA | Previous diagnosis, ADOS, SCQ | ASD group has increased feed-forward connectivity from auditory cortex to parietal lobe and decreased phase-amplitude coupling in the parietal lobe during speech task. |
| **Frequency/Power** | | | | | | | | |
| **Author** | **Year** | **Study Design; Task/Stimulus** | **N** | **Age** | **Sex** | **Region** | **ASD Metric** | **Outcome** |
| Hiraishi et al. (133) | 2014 | Cross-sectional; Watching video (rest) | Total: 76  ASD: 38 TD: 38 | ASD: 5.6 (3.2-7.7) TD: 5.6 (3.0-8.1) | ASD: 0 (0) TD: 0 (0) | Japan | ADOS, DISCO, K-ABC, DSM-IV | TD group has increased right-lateralized theta, alpha, and beta oscillations in the temporal lobe. Leftward lateralization increases with age in the theta, alpha, and gamma bands in the temporal lobe of TD group, but there is no correlation between lateralization index and age in ASD group. |
| Takahashi et al. (134) | 2015 | Cross-sectional; Watching video (rest) | Total: 115  ASD: 43 TD: 72 | ASD: 5.7 (3.3-7.7) TD: 5.5 (3.3-9.2) | ASD: 9 (21) TD: 21 (29) | Japan | ADOS, DISCO, K-ABC, DSM-IV | ASD group has increased multiscale entropy (MSE, measure of brain signal complexity) at younger ages, but TD group has greater increase in MSE over time. |
| Port et al. (135) | 2019 | Cross-sectional; Rest | Total: 166  ASD: 119 TD: 47 | ASD: 9.98 TD: 9.38 | ASD: 14 (12) TD: 15 (32) | USA | Previous diagnosis, ADOS-2, SCQ, SRS-2, ADI-R | ASD group has greater resting power in the alpha, theta and delta bands in bilateral and central posterior, parietal, temporal, and anterior regions compared to TD group. ASD group has lower alpha and theta power in left frontal and central regions compared to TD group. |
| Edgar et al. (136) | 2023 | Cross-sectional; Rest, watching video | Total: 67  ASD: 30 TD: 37 | ASD: 10.0 (6.9-12.6) TD: 9.8 (6.9-12.6) | ASD: 5 (17) TD: 6 (16) | USA | DSM-5, ADOS-2, SCQ, ASRS, SRS-2, OARS, ADI-R | ASD and TD groups both demonstrate peak alpha frequency when eyes are open in a dark room. |
| Manyukhina et al. (137) | 2022 | Cross-sectional; Rest | Total: 98  ASD: 49 TD: 49 | ASD: 10.81 (6-15) TD: 10.29 (6-15) | ASD: 0 (0) TD: 0 (0) | Russia, Sweden | DSM-5, SRS | Spectral slope is flatter (increased excitation compared to inhibition) in low IQ ASD group compared to high IQ ASD group and TD group. |
| Edgar et al. (138) | 2019 | Cross-sectional; Rest | Total: 304  ASD: 183 TD: 121 | ASD: 10.6 (6-17) TD: 10.2 (6-17) | ASD: 0 (0) TD: 0 (0) | USA | Previous diagnosis with DSM-IV or DSM-5, ADOS or ADOS-2, ADI-R, SCQ, SRS-2 | ASD group has stable high peak alpha frequency, while TD group has peak alpha frequency that increases with age |
| Orekhova et al. (139) | 2023 | Cross-sectional; Visual motion task | Total: 79  ASD: 42 TD: 37 | ASD: 10.3 (7-15) TD: 11.0 (7-15) | ASD: 0 (0) TD: 0 (0) | Russia, Sweden | DSM-5, SRS | ASD group does not have an age-associated decrease in gamma response frequency in the primary visual cortex and does not have changes to gamma power or gamma frequency during visual task (TD group has both). |
| Green et al. (140) | 2021 | Cross-sectional; Rest | Total: 121  ASD: 70 TD: 51 | ASD: 12.2 (7-17) TD: 12.9 (6-18) | ASD: 0 (0) TD: 0 (0) | USA | Previous diagnosis with DSM-IV or DSM-5, ADOS or ADOS-2, SCQ | ASD group does not have positive correlations between peak alpha frequency and age or thalamus volume and age (TD group has both). |
| **Longitudinal** | | | | | | | | |
| **Auditory Event Related Field** | | | | | | | | |
| **Author** | **Year** | **Study Design; Task/Stimulus** | **N (at baseline)** | **Age (at baseline)** | **Sex (at baseline)** | **Region** | **ASD Metric** | **Outcome** |
| Green et al. (141) | 2022 | Longitudinal: Two subsequent time points, 1 year apart; Passive auditory and visual stimuli while watching video | Total: 127  ASD: 66  TD: 61 | ASD: 7.7 (6-9) TD: 7.5 (6-9) | ASD: 9 (14) TD: 11 (18) | USA | DSM-5, ADOS-2, SCQ, SRS-2 | No group difference in M50 or M100 latency or amplitude at any time point. M100 is observed more often in ASD group than TD group at T1, but there is no group difference at T2 or T3. TD group has increased phase-locking between all three time points, but ASD group only has increased phase-locking between T1 and T2. ASD group also has greater pre-stimulus power than the TD group in the right hemisphere at T1 and T2, which normalizes by T3. |

# Supplementary Table 9. EEG Findings.

| **Cross-sectional** | | | | | | | | |
| --- | --- | --- | --- | --- | --- | --- | --- | --- |
| **Auditory Event Related Potential** | | | | | | | | |
| **Author** | **Year** | **Study Design; Task/Stimulus** | **N** | **Age** | **Sex** | **Region** | **ASD Metric** | **Outcome** |
| Dwyer et al. (142) | 2021 | Cross-sectional; Passive auditory task while watching video | Total: 211  ASD: 130 TD: 81 | ASD: 3.21 (2.12 - 4.57) TD: 3.09 (2.15-4.69) | ASD: 20 (15) TD: 29 (36) | USA | MSEL, SCQ | Older participants in ASD group have decreased P1 latency in right hemisphere to softer sounds than younger ASD group; TD group has no association between P1 latency and age. TD group has increased N2 latency compared to ASD group after louder sounds, which increases with age. |
| Donkers et al. (143) | 2019 | Cross-sectional; Passive auditory oddball | Total: 67  ASD: 28 TD: 39 | ASD: 7.6 (4-12) TD: 7 (4-12) | ASD: 6 (21) TD: 9 (23) | USA | CARS, ADOS | ASD and DD group have decreased N2 amplitude and ASD group has decreased P3a amplitude in response to auditory oddball. |
| Madsen et al. (144) | 2015 | Cross-sectional; Passive auditory stimuli | Total: 70  ASD: 31  TD: 39 | ASD: 11.1 (8-12)  TD: 10.8 (8-12) | ASD: 7 (23)  TD: 12 (31) | Denmark | ADOS, ADI-R, DSM-IV | No group difference in P50 amplitude or latency. The Asperger’s subgroup of ASD has reduced P50 amplitude to the first stimulus, but not the second. |
| Vlaskamp et al. (145) | 2017 | Cross-sectional; Passive auditory stimuli while watching movie | Total: 73  ASD: 35  TD: 38 | ASD: 11.1 (8-12) TD: 10.8 (8-12) | ASD: 7 (20) TD: 11 (29) | Denmark | DSM-IV, ADOS, ADI-R, Medical records | ASD group has decreased mismatch negativity (MMN) in response to auditory paradigm. ASD group also has increased P3a amplitude following MMN. |
| Cotter et al. (146) | 2023 | Cross-sectional; Audiovisual task | Total: 216  ASD: 84 TD: 132 | ASD: 11.4 (6.1-17.5) TD: 11.5 (6-17.5) | ASD: 12 (14) TD: 70 (53) | USA | ADOS-2, ADI-R | ASD group has decreased auditory evoked potential amplitudes bilaterally. Both groups exhibit rightward lateralized responses to auditory tones, but laterality is decreased in ASD group. |
| **Sensory/Tactile Event Related Potential** | | | | | | | | |
| **Author** | **Year** | **Study Design; Task/Stimulus** | **N** | **Age** | **Sex** | **Region** | **ASD Metric** | **Outcome** |
| Espenhahn et al. (147) | 2021 | Cross-sectional; Passive tactile stimuli while watching movie | Total: 69  ASD: 28 TD: 41 | ASD: 5.4 (3-6) TD: 5.3 (3-6) | ASD: 6 (21) TD: 13 (32) | Canada | ADOS, SRS-2 | No group difference in amplitude or latency of sensory-evoked P50, N80, or P100. ASD group has shorter sensory-evoked N140 (tactile stimulation) latency compared to TD group, suggesting faster processing speed in ASD children. |
| **Visual Event Related Potential** | | | | | | | | |
| **Author** | **Year** | **Study Design; Task/Stimulus** | **N** | **Age** | **Sex** | **Region** | **ASD Metric** | **Outcome** |
| Norcia et al. (148) | 2021 | Cross-sectional; Passive auditory and visual stimuli | Total: 74  ASD: 34 TD: 40 | ASD: 7.0 (4-9) TD: 6.3 (4-9) | ASD: 6 (18) TD: 8 (20) | USA | ADOS-2, ADI-R | ASD group has decreased steady-state visual evoked potentials and attenuated multimodal auditory-visual evoked responses. |
| Manning et al. (149) | 2022 | Cross-sectional; Visual motion game | Total: 100  ASD: 50  TD: 50 | ASD: 10.91 (6.54-14.94)  TD: 10.61 (6.55-14.98) | ASD: 10 (20)  TD: 23 (46) | UK | ADOS, AQ | ASD and TD children do not differ in their response to visual motion tasks: both groups demonstrate a response-locked centroparietal component linked to decision making, with no difference in latency or amplitude. |
| Parker et al. (150) | 2021 | Cross-sectional; Viewing faces | Total: 56  ASD: 30 TD: 26 | ASD: 11.21 (5-18) TD: 10.5 (4-18) | ASD: 6 (20) TD: 11 (42) | USA | DSM-IV, ADOS, ADI-R | TD group has decreased N170 latency when fixating on eyes (as compared to other facial features). This phenomenon is not present in ASD group. |
| Knight et al. (151) | 2023 | Cross-sectional; Viewing Kanizsa stimuli | Total: 60  ASD: 29  TD: 31 | ASD: 13 (7-17)  TD: 12 (7-17) | ASD: 26 (90) TD: 10 (32) | USA | ADOS-2, SRS | ASD children have decreased visual evoked potential amplitudes at multiple occipital sites in response to illusory contour stimuli when compared to TD children. |
| **Motor Event Related Potential** | | | | | | | | |
| **Author** | **Year** | **Study Design; Task/Stimulus** | **N** | **Age** | **Sex** | **Region** | **ASD Metric** | **Outcome** |
| Wakim et al. (152) | 2023 | Cross-sectional; Audiovisual speeded reaction time (AVSRT) task | Total: 168  ASD: 84  TD: 84 | ASD: 11.1 (6.4-19.8) TD: 11.4 (6.4-19.8) | ASD: 16 (19) TD: 22 (26) | USA | Clinical assessment, ADOS | Overall, motor-related cortical potentials are very similar between groups. Significant group-by-electrode interaction. |
| **Attention/Inhibition Event Related Potential** | | | | | | | | |
| **Author** | **Year** | **Study Design; Task/Stimulus** | **N** | **Age** | **Sex** | **Region** | **ASD Metric** | **Outcome** |
| Faja et al. (153) | 2016 | Cross-sectional; Flanker | Total: 61  ASD: 28 TD: 33 | ASD: 9.16 (7.00-11.50) TD: 9.58 (7.41-11.92) | ASD: 3 (11) TD: 3 (9) | USA | ADOS, ADI-R, DSM-5 | ASD group has larger N2 amplitude in response to flanker task. N2 response on flanker is correlated with a similar information suppression task in ASD group. |
| Magnuson et al. (154) | 2020 | Cross-sectional; Emotional Go/NoGo | Total: 55  ASD: 25  TD: 30 | ASD: 10.0 (6-12)  TD: 9.6 (6-12) | ASD: 5 (20)  TD: 9 (30) | UK | ADOS, ADI-R, DSM-5 | ASD children have increased variability in responses to both inhibitory and emotional stimuli during Go/No-Go task, with greater variability in the amplitudes and latencies of the N200 compared to TD group. ASD children have increased intra-subject variability for the N170 amplitude compared to TD group. |
| Clawson et al. (155) | 2017 | Cross-sectional; Flanker | Total: 135  ASD: 62 TD: 73 | ASD: 13.50 (10-17) TD: 12.66 (10-17) | ASD: 0 (0) TD: 0 (0) | USA | SRS, AQ, ADOS-G, DSM-IV | There are no group differences in error-related negativity (ERN) response in the frontal lobe during flanker task. |
| **Emotional Event Related Potential** | | | | | | | | |
| **Author** | **Year** | **Study Design; Task/Stimulus** | **N** | **Age** | **Sex** | **Region** | **ASD Metric** | **Outcome** |
| Liu et al. (156) | 2023 | Cross-sectional; Viewing images of painful stimuli | Total: 56  High AQ: 28  Low AQ: 28 | High AQ: 15.89 (14-17)  Low AQ: 16.26 (14-17) | High AQ: 17 (61) Low AQ: 12 (43) | China | AQ | Group with higher autistic traits has decreased late positive potential (occipital region) and N2 (frontal lobe) amplitudes. |
| **Connectivity** | | | | | | | | |
| **Author** | **Year** | **Study Design; Task/Stimulus** | **N** | **Age** | **Sex** | **Region** | **ASD Metric** | **Outcome** |
| Bochet et al. (157) | 2021 | Cross-sectional; Viewing animated video (rest) | Total: 113  ASD: 66 TD: 47 | ASD: 3.3 (1.8-5.9) TD: 3.3 (1.8-5.8) | ASD: 11 (16) TD: 8 (17) | Switzerland, UAE | ADOS-G or ADOS-2, Clinical assessment | Duration, time coverage, and occurrence of microstates are highly variable within and between groups. The ASD group specifically spends more time in microstate B and has increased transitions from microstates C to B, E to D. |
| Han et al. (158) | 2017 | Cross-sectional; Rest | Total: 187  ASD: 80  TD: 106 | ASD: 5.69 (3-11)  TD: 5.85 (3-11) | ASD: 15 (19)  TD: 27 (25) | China | DSM-5, ABC | ASD group has decreased clustering coefficient in theta, alpha, and beta bands. TD group has age-related decrease in mean shortest path length, ASD group does not. |
| Zhang et al. (159) | 2022 | Cross-sectional; Rest | Total: 367  ASD: 257 TD: 110 | ASD: 6-11 TD: 6-11 | Sex/gender not reported | China | ADOS | ASD group has decreased long-range functional connectivity and hyperconnectivity between the superior frontal gyrus and left middle frontal gyrus. |
| Askari et al. (160) | 2018 | Cross-sectional; Rest | Total: 183  ASD: 89 TD: 94 | ASD: 9.7 (5-12) TD: 9.3 (5-12) | ASD: 36 (40) TD: 33 (35) | Iran | DSM-IV-TR | ASD group has decreased intra-region connectivity in the left frontal, temporal, parietal, and occipital lobes. |
| Pillai et al. (161) | 2018 | Cross-sectional; Pantomiming use of common tools | Total: 58  ASD: 25 TD: 33 | ASD: 10.17 (8-13) TD: 10.52 (8-13) | ASD: 6 (24) TD: 8 (24) | USA | DSM-IV, ADOS, ADI-R | TD group has decreased functional connectivity/trPLV (task-related Phase Locking Value) during motor task task relative to rest. ASD group has increased functional connectivity/trPLV (opposite direction as TD). |
| **Frequency/Power** | | | | | | | | |
| **Author** | **Year** | **Study Design; Task/Stimulus** | **N** | **Age** | **Sex** | **Region** | **ASD Metric** | **Outcome** |
| Portnova et al. (162) | 2020 | Cross-sectional; Listening to nonverbal emotional stimuli, viewing emotional images | Total: 62  ASD: 30 TD: 32 | ASD: 5.4 TD: 5.4 (4-6) | N/A | Russia | ICD-10, CARS, ADOS | ASD group has atypical peak alpha frequency (PAF) while listening to emotional stimuli (increased compared to TD for some, decreased for others). Differences are mostly present in frontal areas. |
| Dickinson et al. (163) | 2017 | Cross-sectional; Passive visual stimuli | Total: 97  ASD: 59 TD: 38 | ASD: 5.79 (2.08-10.50)  TD: 5.96 (2.42-12.16) | ASD: 13 (22) TD: 13 (34) | USA | Clinician assessment, DSM-IV | ASD group has decreased peak-alpha frequency, which is strongly correlated to non-verbal cognition in regions of interest (F3, F4, C3, C4, O1, and O2). |
| Kozhushko et al. (164) | 2018 | Cross-sectional; Rest | Total: 111  ASD: 41  TD: 70 | ASD: 5.9 (4-9)  TD: 6.4 (4-9) | ASD: 6 (15)  TD: 26 (37) | Russia | ICD-10 | ASD children have increased spectral power in theta and beta bands, which is more enhanced in the ASD group with more severe symptoms. |
| Yeung et al. (165) | 2016 | Cross-sectional; Wisconsin Card Sorting Task | Total: 50  ASD: 25 TD: 25 | ASD: 10.09 (6-16) TD: 11.55 (6-17) | ASD: 16 (64) TD: 22 (88) | China | DSM-IV-TR, ADI-R | TD group demonstrates significantly increased task-related theta current density in 600 to 900 ms time window in multiple frontal regions, including the bilateral anterior cingulate cortex, bilateral pre-supplementary motor area, and left ventrolateral prefrontal cortex during Wisconsin Card Sorting Task. |
| Pierce et al. (166) | 2021 | Cross-sectional; Rest | Total: 62  ASD: 31 TD: 31 | ASD: 11.3 (6.5-14.6) TD: 10.6 (6.6-15.0) | ASD: 6 (19) TD: 9 (29) | USA | ADOS, SCQ, DSM-5 | ASD group has lower resting alpha power in posterior regions than TD group. |
| Hornung et al. (167) | 2019 | Cross-sectional; Rest | Total: 64  ASD: 32  TD: 32 | ASD: 12.7 (7.7-17.1)  TD: 12.4 (7.4-16.5) | ASD: 4 (13)  TD: 4 (13) | USA | ADOS, ADI-R, DSM-IV | Children with ASD have decreased overall theta-band activation in frontal, central and posterior regions. |
| Mash et al. (68) | 2020 | Cross-sectional; Rest | Total: 198  ASD: 104  TD: 94 | ASD: 12.6 (7.1-17.1) TD: 13.0 (7.1-18.0) | ASD: 18 (17) TD: 20 (21) | USA | ADOS | ASD group has decreased parieto-occipital alpha power. |
| Neuhaus et al. (168) | 2021 | Cross-sectional; Passive video watching, rest | Total: 280  ASD: 142 TD: 138 | ASD: 12.32 (8-17)  TD: 13.31 (8-17) | ASD: 61 (43)  TD: 68 (49) | USA | ADOS, ADI-R, DSM-IV | ASD group has decreased alpha power across anterior, central, and posterior regions. |
| **Longitudinal** | | | | | | | | |
| **Frequency/Power** | | | | | | | | |
| **Author** | **Year** | **Study Design; Task/Stimulus** | **N (at baseline)** | **Age (at baseline)** | **Sex (at baseline)** | **Region** | **ASD Metric** | **Outcome** |
| Keehn et al. (169) | 2015 | Longitudinal: Time points at 6mo, 12mo, 18mo, 24mo, and 36mo; Viewing images of familiar and unfamiliar faces | Total: 58  FH: 27 NFH: 31 | FH: 193 (177-214) days NFH:  195 (170-223) days | FH 6 months: 15 (56) NFH 6 months: 16 (52) | USA | ADOS, SCQ, SRS | Event-related gamma-band phase coherence between left and right hemispheres during a face viewing task at 6 and 12 months of age demonstrate that FH infants have greater leftward asymmetry of intra-hemispheric coherence over time, whereas NFH infants have increasing rightward lateralization over time. |
| Kolesnik et al. (170) | 2019 | Longitudinal: Time points at 8mo, 12mo, 2yo, and 3yo; Auditory oddball | Total: 142  FH ASD: 116 NFH ASD: 26 | ASD: 8 months  TD: 8 months | HRA: 52 (49) LRA: 13 (48) | UK | One full sibiling with ASD, ADOS, SRS | During auditory oddball task, FH ASD group has diminished auditory repetition suppression of evoked gamma (i.e. elevated cortical reactivity) compared to FH TD group. FH with ASD group has increased inter-trial coherence in the alpha-beta band compared to FH TD group, but no differences in phase-locking in the theta range. |
| Leno et al. (171) | 2021 | Longitudinal: Time points at 12mo, 18mo, and 24mo; Watching videos of toys and faces | Total: 192  FH: 95 NFH: 97 | FH: 12.98 (11-18) months  NFH: 13.03 (11-17) months | Sex/gender not reported | UK, Canada, USA | ADOS, Clinical assessment | There is no group difference in peak alpha frequency (PAF) at 12 months. |
| Haartsen et al. (172) | 2022 | Longitudinal: 2^nd^ time point 1.8 years later; Watching social and non-social videos | Total: 101  No family history (NFH): 26  Family history (FH): 75 | NFH: 1.2  (14-18 months)  FH: 1.2  (14-18 months) | NFH: 17 (65)  FH: 36 (48) | USA | SRS, SCI, RRB | Theta modulations with social content are similar between infants with and without a family history of ASD, for both power and connectivity. |

Supplementary Figure 1. Subcortical Brain Maps. Brain maps depicting subcortical findings for (A) sMRI, (B) DTI, (C) fMRI, and (D) MRS papers. No subcortical regions were described in fNIRS papers, and brain maps were not created for MEG or EEG findings.


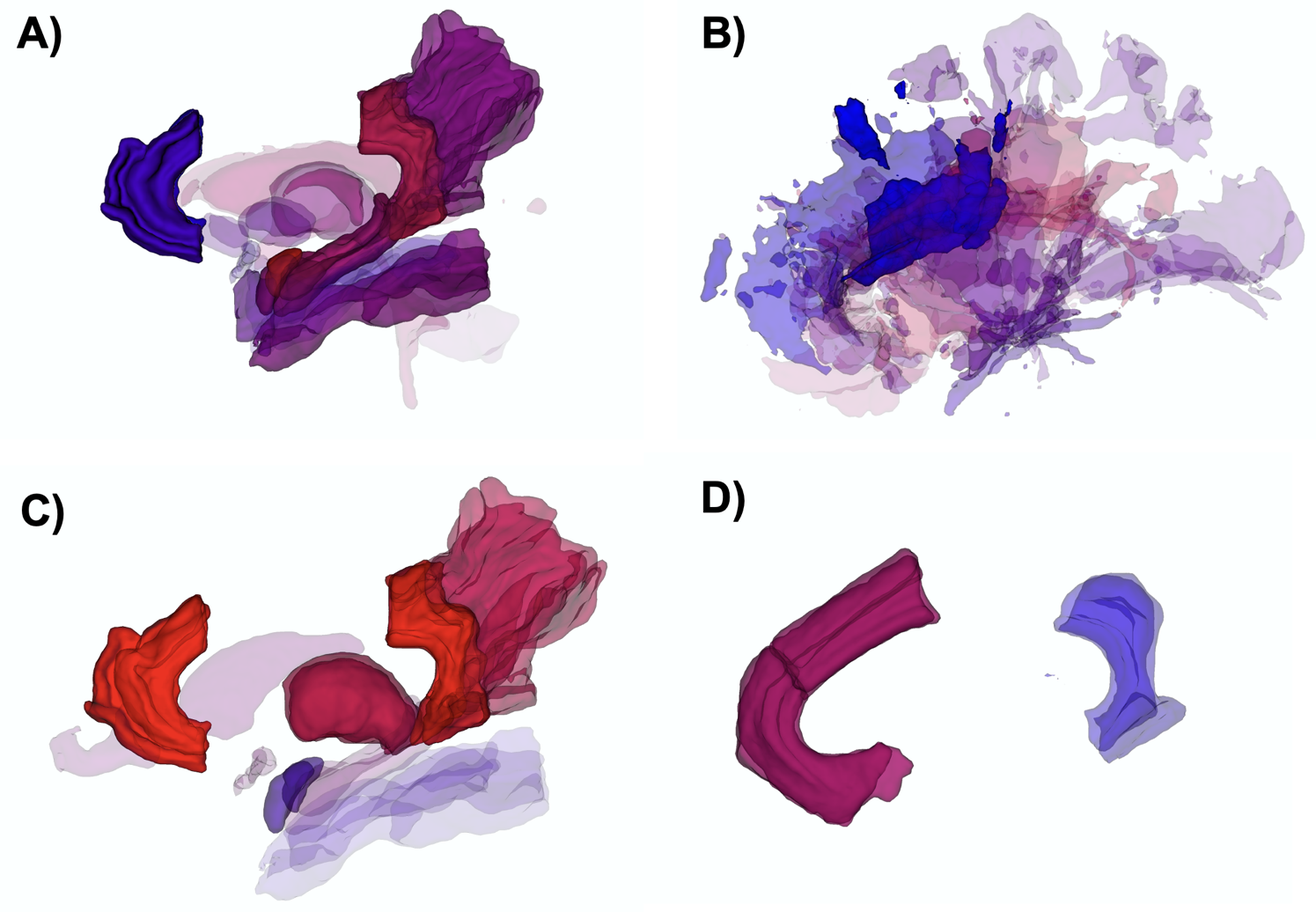


# References

1. Pote I, Wang S, Sethna V, Blasi A, Daly E, Kuklisova‐Murgasova M, et al. Familial risk of autism alters subcortical and cerebellar brain anatomy in infants and predicts the emergence of repetitive behaviors in early childhood. Autism Research. 2019;12(4):614-27.

2. Shiohama T, Ortug A, Warren JLA, Valli B, Levman J, Faja SK, et al. Small Nucleus Accumbens and Large Cerebral Ventricles in Infants and Toddlers Prior to Receiving Diagnoses of Autism Spectrum Disorder. Cerebral Cortex. 2022/03/04;32(6).

3. Xiao Z, Qiu T, Ke X, Xiao X, Xiao T, Liang F, et al. Autism Spectrum Disorder as Early Neurodevelopmental Disorder: Evidence from the Brain Imaging Abnormalities in 2–3 Years Old Toddlers. Journal of Autism and Developmental Disorders 2014 44:7. 2014-01-14;44(7).

4. Zhang Y, Qin B, Wang L, Zhang K, Song C, Chen J, et al. Corpus Callosum Volumes in Children with Autism Spectrum Disorders: Sex-Associated Differences. Journal of Autism and Developmental Disorders 2022 53:6. 2022-03-29;53(6).

5. Nordahl CW, Iosif A-M, Young GS, Hechtman A, Heath B, Lee JK, et al. High Psychopathology Subgroup in Young Children With Autism: Associations With Biological Sex and Amygdala Volume. Journal of the American Academy of Child and Adolescent Psychiatry. 2020/12;59(12).

6. Giuliano A, Saviozzi I, Brambilla P, Muratori F, Retico A, Calderoni S. The effect of age, sex and clinical features on the volume of Corpus Callosum in pre‐schoolers with Autism Spectrum Disorder: a case–control study. European Journal of Neuroscience. 2018/03/01;47(6).

7. Bai C, Wang Y, Zhang Y, Wang X, Chen Z, Yu W, et al. Abnormal gray matter volume and functional connectivity patterns in social cognition‐related brain regions of young children with autism spectrum disorder. Autism Research. 2023/06/01;16(6).

8. Duan X, Wang R, Xiao J, Li Y, Huang X, Guo X, et al. Subcortical structural covariance in young children with autism spectrum disorder. Progress in Neuro-Psychopharmacology and Biological Psychiatry. 2020;99:109874.

9. Erbetta A, Bulgheroni S, Contarino VE, Chiapparini L, Esposito S, Annunziata S, et al. Low-Functioning Autism and Nonsyndromic Intellectual Disability. Journal of Child Neurology. 2015-04-20;30(12).

10. Yang DY-J, Beam D, Pelphrey KA, Abdullahi S, Jou RJ, Yang DY-J, et al. Cortical morphological markers in children with autism: a structural magnetic resonance imaging study of thickness, area, volume, and gyrification. Molecular Autism 2016 7:1. 2016-01-25;7(1).

11. Sharp TH, Elsabbagh M, Pickles A, Bedford R, Sharp TH, Elsabbagh M, et al. The subcortical correlates of autistic traits in school-age children: a population-based neuroimaging study. Molecular Autism 2023 14:1. 2023-02-11;14(1).

12. D'Mello MA, Crocetti D, Mostofsky HS, Stoodley JC. Cerebellar gray matter and lobular volumes correlate with core autism symptoms. NeuroImage: Clinical. 2015;7:631-9.

13. Duerden EG, Card D, Roberts SW, Mak-Fan KM, Chakravarty MM, Lerch JP, et al. Self-injurious behaviours are associated with alterations in the somatosensory system in children with autism spectrum disorder. Brain Structure and Function 2013 219:4. 2013-05-05;219(4).

14. Sussman D, Leung RC, Vogan VM, Lee W, Trelle S, Lin S, et al. The autism puzzle: Diffuse but not pervasive neuroanatomical abnormalities in children with ASD. NeuroImage: Clinical. 2015;8:170-9.

15. Mizuno Y, Kagitani-Shimono K, Jung M, Makita K, Takiguchi S, Fujisawa XT, et al. Structural brain abnormalities in children and adolescents with comorbid autism spectrum disorder and attention-deficit/hyperactivity disorder. Translational Psychiatry. 2019;9(1).

16. Baribeau DA, Dupuis A, Paton TA, Hammill C, Scherer SW, Schachar RJ, et al. Structural neuroimaging correlates of social deficits are similar in autism spectrum disorder and attention-deficit/hyperactivity disorder: analysis from the POND Network. Translational Psychiatry 2019 9:1. 2019-02-04;9(1).

17. Ni H-C, Lin H-Y, Tseng IW-Y, Chiu Y-N, Wu Y-Y, Tsai W-C, Gau S-FS. Neural correlates of impaired self-regulation in male youths with autism spectrum disorder: A voxel-based morphometry study. Progress in Neuro-Psychopharmacology and Biological Psychiatry. 2018;82:233-41.

18. Foster EVN, Doyle-Thomas ARK, Tryfon A, Ouimet T, Anagnostou E, Evans CA, et al. Structural Gray Matter Differences During Childhood Development in Autism Spectrum Disorder: A Multimetric Approach. Pediatric Neurology. 2015;53(4):350-9.

19. Irimia A, Torgerson CM, Jacokes ZJ, Van Horn JD, Irimia A, Torgerson CM, et al. The connectomes of males and females with autism spectrum disorder have significantly different white matter connectivity densities. Scientific Reports 2017 7:1. 2017-04-11;7(1).

20. Ni H-C, Lin H-Y, Chen Y-C, Tseng IW-Y, Gau S-FS. Boys with autism spectrum disorder have distinct cortical folding patterns underpinning impaired self-regulation: a surface-based morphometry study. Brain Imaging and Behavior. 2020;14(6):2464-76.

21. Sharda M, Foster NEV, Tryfon A, Doyle-Thomas KAR, Ouimet T, Anagnostou E, et al. Language Ability Predicts Cortical Structure and Covariance in Boys with Autism Spectrum Disorder. Cerebral Cortex. 2017/03/01;27(3).

22. Mensen VT, Wierenga LM, Dijk Sv, Rijks Y, Oranje B, Mandl RCW, Durston S. Development of cortical thickness and surface area in autism spectrum disorder. NeuroImage : Clinical. 2017;13.

23. Shen MD, Swanson MR, Wolff JJ, Elison JT, Girault JB, Kim SH, et al. Subcortical Brain Development in Autism and Fragile X Syndrome: Evidence for Dynamic, Age- and Disorder-Specific Trajectories in Infancy. American Journal of Psychiatry. 2022-03-25;179(8).

24. Wolff JJ, Gerig G, Lewis JD, Soda T, Styner MA, Vachet C, et al. Altered corpus callosum morphology associated with autism over the first 2 years of life. Brain. 2015/07/01;138(7).

25. Shen DM, Kim HS, Mckinstry CR, Gu H, Hazlett CH, Nordahl WC, et al. Increased Extra-axial Cerebrospinal Fluid in High-Risk Infants Who Later Develop Autism. Biological Psychiatry. 2017;82(3):186-93.

26. Libero EL, Schaer M, Li DD, Amaral GD, Nordahl WC. A Longitudinal Study of Local Gyrification Index in Young Boys With Autism Spectrum Disorder. Cerebral Cortex. 2019;29(6):2575-87.

27. Reinhardt PV, Iosif A-M, Libero L, Heath B, Rogers JS, Ferrer E, et al. Understanding Hippocampal Development in Young Children With Autism Spectrum Disorder. Journal of the American Academy of Child & Adolescent Psychiatry. 2020;59(9):1069-79.

28. Lee KJ, Andrews SD, Ozonoff S, Solomon M, Rogers S, Amaral GD, Nordahl WC. Longitudinal Evaluation of Cerebral Growth Across Childhood in Boys and Girls With Autism Spectrum Disorder. Biological Psychiatry. 2021;90(5):286-94.

29. Lee JK, Andrews DS, Ozturk A, Solomon M, Rogers S, Amaral DG, Nordahl CW. Altered Development of Amygdala-Connected Brain Regions in Males and Females with Autism. Journal of Neuroscience. 2022-08-03;42(31).

30. Solso S, Xu R, Proudfoot J, Hagler JD, Campbell K, Venkatraman V, et al. Diffusion Tensor Imaging Provides Evidence of Possible Axonal Overconnectivity in Frontal Lobes in Autism Spectrum Disorder Toddlers. Biological Psychiatry. 2016;79(8):676-84.

31. Andrews DS, Lee JK, Solomon M, Rogers SJ, Amaral DG, Nordahl CW, et al. A diffusion-weighted imaging tract-based spatial statistics study of autism spectrum disorder in preschool-aged children. Journal of Neurodevelopmental Disorders 2019 11:1. 2019-12-16;11(1).

32. Yu Q, Peng Y, Kang H, Peng Q, Ouyang M, Slinger M, et al. Differential White Matter Maturation from Birth to 8 Years of Age. Cerebral Cortex. 2020/04/14;30(4).

33. Aoki Y, Yoncheva YN, Chen B, Nath T, Sharp D, Lazar M, et al. Association of White Matter Structure With Autism Spectrum Disorder and Attention-Deficit/Hyperactivity Disorder. JAMA Psychiatry. 2017/11;74(11).

34. Li Y, Fang H, Zheng W, Qian L, Xiao Y, Wu Q, et al. A Fiber Tractography Study of Social-Emotional Related Fiber Tracts in Children and Adolescents with Autism Spectrum Disorder. Neuroscience Bulletin 2017 33:6. 2017-07-10;33(6).

35. Roberts TPL, Lanza MR, Dell J, Qasmieh S, Hines K, Blaskey L, et al. Maturational differences in thalamocortical white matter microstructure and auditory evoked response latencies in autism spectrum disorders. Brain research. 2013/11/11;1537.

36. Roberts TPL, Heiken K, Zarnow D, Dell J, Nagae L, Blaskey L, et al. Left Hemisphere Diffusivity of the Arcuate Fasciculus: Influences of Autism Spectrum Disorder and Language Impairment. AJNR: American Journal of Neuroradiology. 2014/03;35(3).

37. Roberts LPT, Bloy L, Ku M, Blaskey L, Jackel RC, Edgar CJ, Berman IJ. A Multimodal Study of the Contributions of Conduction Velocity to the Auditory Evoked Neuromagnetic Response: Anomalies in Autism Spectrum Disorder. Autism Research. 2020;13(10):1730-45.

38. Carper AR, Solders S, Treiber MJ, Fishman I, Müller R-A. Corticospinal Tract Anatomy and Functional Connectivity of Primary Motor Cortex in Autism. Journal of the American Academy of Child & Adolescent Psychiatry. 2015;54(10):859-67.

39. Solders SK, Carper RA, Müller R-A. White matter compromise in autism? Differentiating motion confounds from true differences in diffusion tensor imaging. Autism Research. 2017/10/01;10(10).

40. Hau J, Aljawad S, Baggett N, Fishman I, Carper RA, Müller R-A. The cingulum and cingulate U‐fibers in children and adolescents with autism spectrum disorders. Human Brain Mapping. 2019/08/01;40(11).

41. Nair A, Carper RA, Abbott AE, Chen CP, Solders S, Nakutin S, et al. Regional specificity of aberrant thalamocortical connectivity in autism. Human Brain Mapping. 2015/11;36(11).

42. Fishman I, Datko M, Cabrera Y, Carper RA, Müller R-A. Reduced Integration and Differentiation of the Imitation Network in Autism: A Combined Functional Connectivity Magnetic Resonance Imaging and Diffusion-Weighted Imaging Study. Annals of neurology. 2015/12;78(6).

43. Carper AR, Treiber MJ, Dejesus YS, Müller R-A. Reduced Hemispheric Asymmetry of White Matter Microstructure in Autism Spectrum Disorder. Journal of the American Academy of Child & Adolescent Psychiatry. 2016;55(12):1073-80.

44. Andrews SD, Lee KJ, Harvey JD, Waizbard-Bartov E, Solomon M, Rogers JS, et al. A Longitudinal Study of White Matter Development in Relation to Changes in Autism Severity Across Early Childhood. Biological Psychiatry. 2021;89(5):424-32.

45. Zhang M, Hu X, Jiao J, Yuan D, Li S, Luo T, et al. Brain white matter microstructure abnormalities in children with optimal outcome from autism: a four-year follow-up study. Scientific Reports 2022 12:1. 2022-11-23;12(1).

46. Wang Y, Hu D, Wu Z, Wang L, Huang W, Li G. Developmental abnormalities of structural covariance networks of cortical thickness and surface area in autistic infants within the first 2 years. Cerebral Cortex (New York, NY). 2022/09/09;32(17).

47. Zielinski AB, Andrews SD, Lee KJ, Solomon M, Rogers JS, Heath B, et al. Sex-dependent structure of socioemotional salience, executive control, and default mode networks in preschool-aged children with autism. NeuroImage. 2022;257:119252.

48. Shen DM, Li DD, Keown LC, Lee A, Johnson TR, Angkustsiri K, et al. Functional Connectivity of the Amygdala Is Disrupted in Preschool-Aged Children With Autism Spectrum Disorder. Journal of the American Academy of Child & Adolescent Psychiatry. 2016;55(9):817-24.

49. Xiao Y, Wen TH, Kupis L, Eyler LT, Goel D, Vaux K, et al. Neural responses to affective speech, including motherese, map onto clinical and social eye tracking profiles in toddlers with ASD. Nature Human Behaviour 2022 6:3. 2022-01-03;6(3).

50. He C, Chen Y, Jian T, Chen H, Guo X, Wang J, et al. Dynamic functional connectivity analysis reveals decreased variability of the default‐mode network in developing autistic brain. Autism Research. 2018/11/01;11(11).

51. He C, Cortes JM, Kang X, Cao J, Chen H, Guo X, et al. Individual‐based morphological brain network organization and its association with autistic symptoms in young children with autism spectrum disorder. Human Brain Mapping. 2021/07/01;42(10).

52. Shi F, Wang L, Peng Z, Wee C-Y, Shen D. Altered Modular Organization of Structural Cortical Networks in Children with Autism. PLOS ONE. May 10, 2013;8(5).

53. Floris DL, Lai M-C, Auer T, Lombardo MV, Ecker C, Chakrabarti B, et al. Atypically rightward cerebral asymmetry in male adults with autism stratifies individuals with and without language delay. Human Brain Mapping. 2016/01/01;37(1).

54. Martino DA, Zuo X-N, Kelly C, Grzadzinski R, Mennes M, Schvarcz A, et al. Shared and Distinct Intrinsic Functional Network Centrality in Autism and Attention-Deficit/Hyperactivity Disorder. Biological Psychiatry. 2013;74(8):623-32.

55. Dajani DR, Burrows CA, Odriozola P, Baez A, Nebel MB, Mostofsky SH, Uddin LQ. Investigating functional brain network integrity using a traditional and novel categorical scheme for neurodevelopmental disorders. NeuroImage: Clinical. 2019/01/01;21.

56. Supekar K, Ryali S, Mistry P, Menon V, Supekar K, Ryali S, et al. Aberrant dynamics of cognitive control and motor circuits predict distinct restricted and repetitive behaviors in children with autism. Nature Communications 2021 12:1. 2021-06-10;12(1).

57. Gooskens B, Bos JD, Mensen TV, Shook AD, Bruchhage MKM, Naaijen J, et al. No evidence of differences in cognitive control in children with autism spectrum disorder or obsessive-compulsive disorder: An fMRI study. Developmental Cognitive Neuroscience. 2019;36:100602.

58. Mcnaughton AK, Kirby AL, Warnell RK, Alkire D, Merchant SJ, Moraczewski D, et al. Social-interactive reward elicits similar neural response in autism and typical development and predicts future social experiences. Developmental Cognitive Neuroscience. 2023;59:101197.

59. Kilroy E, Harrison L, Butera C, Jayashankar A, Cermak S, Kaplan J, et al. Unique deficit in embodied simulation in autism: An fMRI study comparing autism and developmental coordination disorder. Human Brain Mapping. 2021/04/04;42(5).

60. Butera C, Kaplan J, Kilroy E, Harrison L, Jayashankar A, Loureiro F, Aziz-Zadeh L. The relationship between alexithymia, interoception, and neural functional connectivity during facial expression processing in autism spectrum disorder. Neuropsychologia. 2023;180:108469.

61. Bos JD, Raalten VRT, Oranje B, Smits RA, Kobussen AN, Belle VJ, et al. Developmental differences in higher-order resting-state networks in Autism Spectrum Disorder. NeuroImage: Clinical. 2014;4:820-7.

62. Yerys BE, Herrington JD, Satterthwaite TD, Guy L, Schultz RT, Bassett DS, et al. Globally weaker and topologically different: resting-state connectivity in youth with autism. Molecular Autism 2017 8:1. 2017-07-26;8(1).

63. Yerys EB, Tunç B, Satterthwaite DT, Antezana L, Mosner GM, Bertollo RJ, et al. Functional Connectivity of Frontoparietal and Salience/Ventral Attention Networks Have Independent Associations With Co-occurring Attention-Deficit/Hyperactivity Disorder Symptoms in Children With Autism. Biological Psychiatry: Cognitive Neuroscience and Neuroimaging. 2019;4(4):343-51.

64. Lin H-Y, Ni H-C, Tseng W-YI, Gau SS-F, Hsiang-Yuan Lin H-CN, Wen-Yih Isaac Tseng, Susan Shur-Fen Gau. Characterizing intrinsic functional connectivity in relation to impaired self-regulation in intellectually able male youth with autism spectrum disorder. Autism. 2020-01-21.

65. Doyle-Thomas KAR, Lee W, Foster NEV, Tryfon A, Ouimet T, Hyde KL, et al. Atypical functional brain connectivity during rest in autism spectrum disorders. Annals of Neurology. 2015/05/01;77(5).

66. Fan L-Y, Booth JR, Liu M, Chou T-L, Gau SS-F. Developmental differences in neural connectivity

for semantic processing in youths with autism. Journal of Child Psychology and Psychiatry. 2021/09/01;62(9).

67. Vogan VM, Francis KE, Morgan BR, Smith ML, Taylor MJ, Vogan VM, et al. Load matters: neural correlates of verbal working memory in children with autism spectrum disorder. Journal of Neurodevelopmental Disorders 2018 10:1. 2018-06-01;10(1).

68. Mash EL, Keehn B, Linke CA, Liu TT, Helm LJ, Haist F, et al. Atypical Relationships Between Spontaneous EEG and fMRI Activity in Autism. Brain Connectivity. 2020;10(1):18-28.

69. Green SA, Hernandez L, Bookheimer SY, Dapretto M. Salience Network Connectivity in Autism Is Related to Brain and Behavioral Markers of Sensory Overresponsivity. Journal of the American Academy of Child & Adolescent Psychiatry. 2016/07/01;55(7).

70. Zhao X, Zhu S, Cao Y, Cheng P, Lin Y, Sun Z, et al. Regional homogeneity of adolescents with high‐functioning autism spectrum disorder and its association with symptom severity. Brain and Behavior. 2022/08/01;12(8).

71. Mash LE, Linke AC, Olson LA, Fishman I, Liu TT, Müller R-A. Transient states of network connectivity are atypical in autism: A dynamic functional connectivity study. Human Brain Mapping. 2019/06/01;40(8).

72. Lawrence KE, Hernandez LM, Bowman HC, Padgaonkar NT, Fuster E, Jack A, et al. Sex Differences in Functional Connectivity of the Salience, Default Mode, and Central Executive Networks in Youth with ASD. Cerebral Cortex. 2020/07/30;30(9).

73. Abbott EA, Nair A, Keown LC, Datko M, Jahedi A, Fishman I, Müller R-A. Patterns of Atypical Functional Connectivity and Behavioral Links in Autism Differ Between Default, Salience, and Executive Networks. Cerebral Cortex. 2016;26(10):4034-45.

74. Fishman I, Linke AC, Hau J, Carper RA, Muller R-A. Atypical Functional Connectivity of Amygdala Related to Reduced Symptom Severity in Children With Autism. Journal of the American Academy of Child & Adolescent Psychiatry. 2018/10/01;57(10).

75. Keehn JJR, Pueschel BE, Gao Y, Jahedi A, Alemu K, Carper R, et al. Underconnectivity Between Visual and Salience Networks and Links With Sensory Abnormalities in Autism Spectrum Disorders. Journal of the American Academy of Child & Adolescent Psychiatry. 2021;60(2):274-85.

76. Keown LC, Shih P, Nair A, Peterson N, Mulvey EM, Müller R-A. Local Functional Overconnectivity in Posterior Brain Regions Is Associated with Symptom Severity in Autism Spectrum Disorders. Cell Reports. 2013;5(3):567-72.

77. Gao Y, Linke A, Keehn RJJ, Punyamurthula S, Jahedi A, Gates K, et al. The language network in autism: Atypical functional connectivity with default mode and visual regions. Autism Research. 2019/09/01;12(9).

78. Jung J, Zbozinek TD, Cummings KK, Wilhelm FH, Dapretto M, Craske MG, et al. Associations between physiological and neural measures of sensory reactivity in youth with autism. Journal of child psychology and psychiatry, and allied disciplines. 2021/10;62(10).

79. Khan AJ, Nair A, Keown CL, Datko MC, Lincoln AJ, Müller R-A. Cerebro-cerebellar resting state functional connectivity in children and adolescents with autism spectrum disorder. Biological psychiatry. 2015/11/11;78(9).

80. Wood ET, Cummings KK, Jung J, Patterson G, Okada N, Guo J, et al. Sensory over-responsivity is related to GABAergic inhibition in thalamocortical circuits. Translational Psychiatry 2021 11:1. 2021-01-12;11(1).

81. Linke CA, Keehn JJR, Pueschel BE, Fishman I, Müller R-A. Children with ASD show links between aberrant sound processing, social symptoms, and atypical auditory interhemispheric and thalamocortical functional connectivity. Developmental Cognitive Neuroscience. 2018;29:117-26.

82. Solomon M, Yoon HJ, Ragland DJ, Niendam AT, Lesh AT, Fairbrother W, Carter SC. The Development of the Neural Substrates of Cognitive Control in Adolescents with Autism Spectrum Disorders. Biological Psychiatry. 2014;76(5):412-21.

83. Martino AD, Yan C-G, Li Q, Denio E, Castellanos FX, Alaerts K, et al. The Autism Brain Imaging Data Exchange: Towards Large-Scale

Evaluation of the Intrinsic Brain Architecture in Autism. Molecular psychiatry. 2014/06;19(6).

84. Liu J, Okada JN, Cummings KK, Jung J, Patterson G, Bookheimer YS, et al. Emerging atypicalities in functional connectivity of language-related networks in young infants at high familial risk for ASD. Developmental Cognitive Neuroscience. 2020;45:100814.

85. Linke CA, Chen B, Olson L, Ibarra C, Fong C, Reynolds S, et al. Sleep Problems in Preschoolers With Autism Spectrum Disorder Are Associated With Sensory Sensitivities and Thalamocortical Overconnectivity. Biological Psychiatry: Cognitive Neuroscience and Neuroimaging. 2023;8(1):21-31.

86. Mori K, Toda Y, Ito H, Mori T, Goji A, Fujii E, et al. A proton magnetic resonance spectroscopic study in autism spectrum disorders: Amygdala and orbito-frontal cortex. Brain and Development. 2013/02/01;35(2).

87. Goji A, Ito H, Mori K, Harada M, Hisaoka S, Toda Y, et al. Assessment of Anterior Cingulate Cortex (ACC) and Left Cerebellar Metabolism in Asperger's Syndrome with Proton Magnetic Resonance Spectroscopy (MRS). PLOS ONE. Jan 6, 2017;12(1).

88. Ito H, Mori K, Harada M, Hisaoka S, Toda Y, Mori T, et al. A Proton Magnetic Resonance Spectroscopic Study in Autism Spectrum Disorder Using a 3-Tesla Clinical Magnetic Resonance Imaging (MRI) System: The Anterior Cingulate Cortex and the Left Cerebellum. Journal of Child Neurology. 2017-04-19.

89. DeMayo MM, Harris AD, Song YJC, Pokorski I, Thapa R, Patel S, et al. Age‐related parietal GABA alterations in children with autism spectrum disorder. Autism Research. 2021/05/01;14(5).

90. He JL, Oeltzschner G, Mikkelsen M, Deronda A, Harris AD, Crocetti D, et al. Region-specific elevations of glutamate + glutamine correlate with the sensory symptoms of autism spectrum disorders. Translational Psychiatry 2021 11:1. 2021-07-29;11(1).

91. Puts NAJ, Wodka EL, Harris AD, Crocetti D, Tommerdahl M, Mostofsky SH, Edden RAE. Reduced GABA and altered somatosensory function in children with autism spectrum disorder. Autism Research. 2017/04/01;10(4).

92. Naaijen J, Zwiers MP, Amiri H, Williams SCR, Durston S, Oranje B, et al. Fronto-Striatal Glutamate in Autism Spectrum Disorder and Obsessive Compulsive Disorder. Neuropsychopharmacology 2017 42:12. 2016-11-21;42(12).

93. Naaijen J, Zwiers MP, Forde NJ, Williams SC, Durston S, Brandei D, et al. Striatal structure and its association with N-Acetylaspartate and glutamate in autism spectrum disorder and obsessive compulsive disorder. European Neuropsychopharmacology. 2018/01/01;28(1).

94. Edmondson DA, Xia P, Keehn RM, Dydak U, Keehn B. A Magnetic Resonance Spectroscopy Study of Superior Visual Search Abilities in Children with Autism Spectrum Disorder. Autism Research. 2020/04/01;13(4).

95. Hardan AY, Fung LK, Frazier T, Berquist SW, Minshew NJ, Keshavan MS, Stanley JA. A proton spectroscopy study of white matter in children with autism. Progress in Neuro-Psychopharmacology and Biological Psychiatry. 2016/04/03;66.

96. Doyle-Thomas KAR, Card D, Soorya LV, Wang AT, Fan J, Anagnostou E. Metabolic mapping of deep brain structures and associations with symptomatology in autism spectrum disorders. Research in Autism Spectrum Disorders. 2014/01/01;8(1).

97. Carvalho Pereira A, Violante IR, Mouga S, Oliveira G, Castelo-Branco M, Carvalho Pereira A, et al. Medial Frontal Lobe Neurochemistry in Autism Spectrum Disorder is Marked by Reduced N-Acetylaspartate and Unchanged Gamma-Aminobutyric Acid and Glutamate + Glutamine Levels. Journal of Autism and Developmental Disorders 2017 48:5. 2017-11-24;48(5).

98. Rojas DC, Singel D, Steinmetz S, Hepburn S, Brown MS. Decreased left perisylvian GABA concentration in children with autism and unaffected siblings. NeuroImage. 2014/02/01;86.

99. Joshi G, Biederman J, Wozniak J, Goldin RL, Crowley D, Furtak S, et al. Magnetic resonance spectroscopy study of the glutamatergic system in adolescent males with high-functioning autistic disorder: a pilot study at 4T. European Archives of Psychiatry and Clinical Neuroscience 2012 263:5. 2012-09-18;263(5).

100. Cochran DM, Sikoglu EM, Hodge SM, Edden RAE, Foley A, Kennedy DN, et al. Relationship among Glutamine, γ-Aminobutyric Acid, and Social Cognition in Autism Spectrum Disorders. Journal of Child and Adolescent Psychopharmacology. 2015-05-15;25(4).

101. Drenthen SG, Barendse ME, Aldenkamp PA, Veenendaal VMT, Puts AJN, Edden AER, et al. Altered neurotransmitter metabolism in adolescents with high-functioning autism. Psychiatry Research: Neuroimaging. 2016;256:44-9.

102. Corrigan NM, Shaw DWW, Estes AM, Richards TL, Munson J, Friedman SD, et al. Brain Chemistry in Children With ASD. JAMA Psychiatry. 2013/09/01;70(9).

103. Hollestein V, Buitelaar JK, Brandeis D, Banaschewski T, Kaiser A, Hohmann S, et al. Developmental changes in fronto-striatal glutamate and their association with functioning during inhibitory control in autism spectrum disorder and obsessive compulsive disorder. NeuroImage: Clinical. 2021/01/01;30.

104. Edwards AL, Wagner BJ, Tager-Flusberg H, Nelson AC. Differences in Neural Correlates of Speech Perception in 3 Month Olds at High and Low Risk for Autism Spectrum Disorder. Journal of Autism and Developmental Disorders. 2017;47(10):3125-38.

105. Braukmann R, Lloyd‐Fox S, Blasi A, Johnson HM, Bekkering H, Buitelaar KJ, Hunnius S. Diminished socially selective neural processing in 5‐month‐old infants at high familial risk of autism. European Journal of Neuroscience. 2018;47(6):720-8.

106. Bhat AN, Mcdonald NM, Eilbott JE, Pelphrey KA. Exploring cortical activation and connectivity in infants with and without familial risk for autism during naturalistic social interactions: A preliminary study. Infant Behavior and Development. 2019;57:101337.

107. Hou S, Liu N, Zou J, Yin X, Liu X, Zhang S, et al. Young children with autism show atypical prefrontal cortical responses to humanoid robots: An fNIRS study. International Journal of Psychophysiology. 2022;181:23-32.

108. Kikuchi M, Yoshimura Y, Shitamichi K, Ueno S, Hiraishi H, Munesue T, et al. Anterior Prefrontal Hemodynamic Connectivity in Conscious 3- to 7-Year-Old Children with Typical Development and Autism Spectrum Disorder. PLoS ONE. 2013;8(2).

109. Jia H, Li Y, Yu D, Jia H, Li Y, Yu D. Normalized spatial complexity analysis of neural signals. Scientific Reports 2018 8:1. 2018-05-21;8(1).

110. Li Y, Yu D. Weak network efficiency in young children with Autism Spectrum Disorder: Evidence from a functional near-infrared spectroscopy study. Brain and Cognition. 2016;108:47-55.

111. Jia H, Li Y, Yu D. Attenuation of long-range temporal correlations of neuronal oscillations in young children with autism spectrum disorder. NeuroImage: Clinical. 2018;20:424-32.

112. Lin F, Hu Y, Huang W, Wu X, Sun H, Li J. Resting‐state coupling between HbO and Hb measured by fNIRS in autism spectrum disorder. Journal of Biophotonics. 2023/03/01;16(3).

113. Zhang T, Huang W, Wu X, Sun W, Lin F, Sun H, et al. Altered complexity in resting-state fNIRS signal in autism: a multiscale entropy approach. Physiological Measurement. 2021-08-27;42(8).

114. Yasumura A, Kokubo N, Yamamoto H, Yasumura Y, Nakagawa E, Kaga M, et al. Neurobehavioral and hemodynamic evaluation of Stroop and reverse Stroop interference in children with attention-deficit/hyperactivity disorder. Brain and Development. 2014;36(2):97-106.

115. Iwanaga R, Tanaka G, Nakane H, Honda S, Imamura A, Ozawa H. Usefulness of near‐infrared spectroscopy to detect brain dysfunction in children with autism spectrum disorder when inferring the mental state of others. Psychiatry and Clinical Neurosciences. 2013;67(4):203-9.

116. Mori K, Toda Y, Ito H, Mori T, Mori K, Goji A, et al. Neuroimaging in autism spectrum disorders: 1H-MRS and NIRS study. The Journal of Medical Investigation. 2015;62(1.2).

117. Schudlo LC, Anagnostou E, Chau T, Doyle-Thomas K. Investigating sensory response to physical discomfort in children with autism spectrum disorder using near-infrared spectroscopy. PLOS ONE. Sep 3, 2021;16(9).

118. Sun H, Lin F, Wu X, Zhang T, Li J. Normalized mutual information of fNIRS signals as a measure for accessing typical and atypical brain activity. Journal of Biophotonics. 2023/06/01;16(6).

119. Yeung KM, Lee LT, Chan SA. Right-lateralized frontal activation underlies successful updating of verbal working memory in adolescents with high-functioning autism spectrum disorder. Biological Psychology. 2019;148:107743.

120. Yeung MK, Lee TL, Chan AS. Frontal lobe dysfunction underlies the differential word retrieval impairment in adolescents with high‐functioning autism. Autism Research. 2019/04/01;12(4).

121. Jung CE, Strother L, Feil-Seifer DJ, Hutsler JJ. Atypical Asymmetry for Processing Human and Robot Faces in Autism Revealed by fNIRS. PLOS ONE. Jul 7, 2016;11(7).

122. Pecukonis M, Perdue LK, Wong J, Tager-Flusberg H, Nelson AC. Exploring the relation between brain response to speech at 6-months and language outcomes at 24-months in infants at high and low risk for autism spectrum disorder: A preliminary functional near-infrared spectroscopy study. Developmental Cognitive Neuroscience. 2021;47:100897.

123. Zhang F, Moerman F, Niu H, Warreyn P, Roeyers H. Atypical brain network development of infants at elevated likelihood for autism spectrum disorder during the first year of life. Autism Research. 2022/12/01;15(12).

124. Yoshimura Y, Kikuchi M, Hayashi N, Hiraishi H, Hasegawa C, Takahashi T, et al. Altered human voice processing in the frontal cortex and a developmental language delay in 3- to 5-year-old children with autism spectrum disorder. Scientific Reports 2017 7:1. 2017-12-07;7(1).

125. Yoshimura Y, Ikeda T, Hasegawa C, An K-M, Tanaka S, Yaoi K, et al. Shorter P1m Response in Children with Autism Spectrum Disorder without Intellectual Disabilities. International Journal of Molecular Sciences 2021, Vol 22, Page 2611. 2021-03-05;22(5).

126. Yoshimura Y, Kikuchi M, Hiraishi H, Hasegawa C, Takahashi T, Remijn GB, et al. Atypical development of the central auditory system in young children with Autism spectrum disorder. Autism Research. 2016/11/01;9(11).

127. Stroganova TA, Komarov KS, Sysoeva OV, Goiaeva DE, Obukhova TS, Ovsiannikova TM, et al. Left hemispheric deficit in the sustained neuromagnetic response to periodic click trains in children with ASD. Molecular Autism 2020 11:1. 2020-12-31;11(1).

128. Edgar JC, Khan SY, Blaskey L, Chow VY, Rey M, Gaetz W, et al. Neuromagnetic Oscillations Predict Evoked-Response Latency Delays and Core Language Deficits in Autism Spectrum Disorders. Journal of Autism and Developmental Disorders 2013 45:2. 2013-08-21;45(2).

129. Duan F, Watanabe K, Yoshimura Y, Kikuchi M, Minabe Y, Aihara K. Detection of atypical network development patterns in children with autism spectrum disorder using magnetoencephalography. PLOS ONE. Sep 8, 2017;12(9).

130. Kikuchi M, Shitamichi K, Yoshimura Y, Ueno S, Hiraishi H, Hirosawa T, et al. Altered brain connectivity in 3-to 7-year-old children with autism spectrum disorder. NeuroImage: Clinical. 2013/01/01;2.

131. Kikuchi M, Yoshimura Y, Hiraishi H, Munesue T, Hashimoto T, Tsubokawa T, et al. Reduced long-range functional connectivity in young children with autism spectrum disorder. Social Cognitive and Affective Neuroscience. 2015/02/01;10(2).

132. Alho J, Khan S, Mamashli F, Perrachione TK, Losh A, McGuiggan NM, et al. Atypical cortical processing of bottom-up speech binding cues in children with autism spectrum disorders. NeuroImage: Clinical. 2023/01/01;37.

133. Hiraishi H, Kikuchi M, Yoshimura Y, Kitagawa S, Hasegawa C, Munesue T, et al. Unusual developmental pattern of brain lateralization in young boys with autism spectrum disorder: Power analysis with child‐sized magnetoencephalography. Psychiatry and Clinical Neurosciences. 2015/03/01;69(3).

134. Takahashi T, Yoshimura Y, Hiraishi H, Hasegawa C, Munesue T, Higashida H, et al. Enhanced brain signal variability in children with autism spectrum disorder during early childhood. Human Brain Mapping. 2016/03/01;37(3).

135. Port RG, Dipiero MA, Ku M, Liu S, Blaskey L, Kuschner ES, et al. Children with Autism Spectrum Disorder Demonstrate Regionally Specific Altered Resting-State Phase–Amplitude Coupling. Brain Connectivity. 2019-06-05.

136. Edgar JC, Franzen RE, McNamee M, Green HL, Shen G, DiPiero M, et al. A comparison of resting‐state eyes‐closed and dark‐room alpha‐band activity in children. Psychophysiology. 2023/06/01;60(6).

137. Manyukhina VO, Prokofyev AO, Galuta IA, Goiaeva DE, Obukhova TS, Schneiderman JF, et al. Globally elevated excitation–inhibition ratio in children with autism spectrum disorder and below-average intelligence. Molecular Autism 2022 13:1. 2022-05-12;13(1).

138. Edgar JC, Dipiero M, McBride E, Green HL, Berman J, Ku M, et al. Abnormal maturation of the resting‐state peak alpha frequency in children with autism spectrum disorder. Human Brain Mapping. 2019/08/01;40(11).

139. Orekhova EV, Manyukhina VO, Galuta IA, Prokofyev AO, Goiaeva DE, Obukhova TS, et al. Gamma oscillations point to the role of primary visual cortex in atypical motion processing in autism. PLOS ONE. Feb 13, 2023;18(2).

140. Green HL, Dipiero M, Koppers S, Berman JI, Bloy L, Liu S, et al. Peak Alpha Frequency and Thalamic Structure in Children with Typical Development and Autism Spectrum Disorder. Journal of Autism and Developmental Disorders 2021 52:1. 2021-02-25;52(1).

141. Green HL, Shen G, Franzen RE, Mcnamee M, Berman JI, Mowad TG, et al. Differential Maturation of Auditory Cortex Activity in Young Children with Autism and Typical Development. Journal of Autism and Developmental Disorders 2022 53:10. 2022-08-12;53(10).

142. Dwyer P, Meo-Monteil DR, Saron DC, Rivera MS. Effects of age on loudness-dependent auditory ERPs in young autistic and typically-developing children. Neuropsychologia. 2021;156:107837.

143. Donkers FC, Carlson M, Schipul SE, Belger A, Baranek GT, Franc CL Donkers MC, Sarah E Schipul, Aysenil Belger, Grace T Baranek. Auditory event-related potentials and associations with sensory patterns in children with autism spectrum disorder, developmental delay, and typical development. Autism. 2019-12-17;24(5).

144. Madsen GF, Bilenberg N, Jepsen JR, Glenthøj B, Cantio C, Oranje B. Normal P50 Gating in Children with Autism, Yet Attenuated P50 Amplitude in the Asperger Subcategory. Autism Research. 2015/08/01;8(4).

145. Vlaskamp C, Oranje B, Madsen GF, Jepsen JRM, Durston S, Cantio C, et al. Auditory processing in autism spectrum disorder: Mismatch negativity deficits. Autism Research. 2017/11/01;10(11).

146. Cotter M, Reisli S, Francisco AA, Wakim K-M, Oakes L, Crosse MJ, et al. Neurophysiological measures of auditory sensory processing are associated with adaptive behavior in children with Autism Spectrum Disorder. Journal of Neurodevelopmental Disorders 2023 15:1. 2023-04-01;15(1).

147. Espenhahn S, Godfrey KJ, Kaur S, Ross M, Nath N, Dmitrieva O, et al. Tactile cortical responses and association with tactile reactivity in young children on the autism spectrum. Molecular Autism 2021 12:1. 2021-04-01;12(1).

148. Norcia AM, Lee A, Meredith WJ, Kohler PJ, Pei F, Ghassan SA, et al. A case–control study of visual, auditory and audio–visual sensory interactions in children with autism spectrum disorder. Journal of Vision. 2021/04/01;21(4).

149. Manning C, Hassall DC, Hunt TL, Norcia MA, Wagenmakers E-J, Evans JN, Scerif G. Behavioural and neural indices of perceptual decision-making in autistic children during visual motion tasks. Scientific Reports. 2022;12(1).

150. Parker CT, Crowley JM, Naples JA, Rolison JM, Wu J, Trapani AJ, Mcpartland CJ. The N170 event‐related potential reflects delayed neural response to faces when visual attention is directed to the eyes in youths with ASD. Autism Research. 2021;14(7):1347-56.

151. Knight JE, Freedman GE, Myers JE, Berruti SA, Oakes AL, Cao ZC, et al. Severely Attenuated Visual Feedback Processing in Children on the Autism Spectrum. The Journal of Neuroscience. 2023;43(13):2424-38.

152. Wakim K-M, Foxe JJ, Molholm S. Cued motor processing in autism and typical development: A high‐density electrical mapping study of response‐locked neural activity in children and adolescents. European Journal of Neuroscience. 2023/08/01;58(3).

153. Faja S, Clarkson T, Webb JS. Neural and behavioral suppression of interfering flankers by children with and without autism spectrum disorder. Neuropsychologia. 2016;93:251-61.

154. Magnuson JR, Iarocci G, Doesburg SM, Moreno S. Increased Intra‐Subject Variability of Reaction Times and Single‐Trial Event‐Related Potential Components in Children With Autism Spectrum Disorder. Autism Research. 2020/02/01;13(2).

155. Clawson A, South M, Baldwin SA, Larson MJ, Clawson A, South M, et al. Electrophysiological Endophenotypes and the Error-Related Negativity (ERN) in Autism Spectrum Disorder: A Family Study. Journal of Autism and Developmental Disorders 2017 47:5. 2017-02-21;47(5).

156. Liu S, Tang F, Dou H, Zhang W. The relationship between autistic traits and empathy in adolescents: An ERP study. Neuroscience Letters. 2023;802:137173.

157. Bochet A, Sperdin HF, Rihs TA, Kojovic N, Franchini M, Jan RK, et al. Early alterations of large-scale brain networks temporal dynamics in young children with autism. Communications Biology 2021 4:1. 2021-08-16;4(1).

158. Han J, Zeng K, Kang J, Tong Z, Cai E, Chen H, et al. Development of Brain Network in Children with Autism from Early Childhood to Late Childhood. Neuroscience. 2017;367:134-46.

159. Zhang Y, Zhang S, Chen B, Jiang L, Li Y, Dong L, et al. Predicting the Symptom Severity in Autism Spectrum Disorder Based on EEG Metrics | IEEE Journals & Magazine | IEEE Xplore. IEEE Transactions on Neural Systems and Rehabilitation Engineering. 2022;30.

160. Askari E, Setarehdan KS, Sheikhani A, Mohammadi RM, Teshnehlab M. Modeling the connections of brain regions in children with autism using cellular neural networks and electroencephalography analysis. Artificial Intelligence in Medicine. 2018;89:40-50.

161. Pillai AS, McAuliffe D, Lakshmanan BM, Mostofsky SH, Crone NE, Ewen JB. Altered task‐related modulation of long‐range connectivity in children with autism. Autism Research. 2018/02/01;11(2).

162. Portnova GV, Maslennikova AV. Atypical EEG Responses to Nonverbal Emotionally Charged Stimuli in Children with ASD. Behavioural Neurology. 2020;2020.

163. Dickinson A, DiStefano C, Senturk D, Jeste SS. Peak alpha frequency is a neural marker of cognitive function across the autism spectrum. European Journal of Neuroscience. 2018/03/01;47(6).

164. Kozhushko JN, Nagornova VZ, Evdokimov AS, Shemyakina VN, Ponomarev AV, Tereshchenko PE, Kropotov DJ. Specificity of spontaneous EEG associated with different levels of cognitive and communicative dysfunctions in children. International Journal of Psychophysiology. 2018;128:22-30.

165. Yeung KM, Han YMY, Sze LS, Chan SA. Abnormal frontal theta oscillations underlie the cognitive flexibility deficits in children with high-functioning autism spectrum disorders. Neuropsychology. 2016;30(3):281-95.

166. Pierce S, Kadlaskar G, Edmondson DA, McNally Keehn R, Dydak U, Keehn B, et al. Associations between sensory processing and electrophysiological and neurochemical measures in children with ASD: an EEG-MRS study. Journal of Neurodevelopmental Disorders 2021 13:1. 2021-01-06;13(1).

167. Hornung T, Chan W-H, Müller R-A, Townsend J, Keehn B. Dopaminergic hypo-activity and reduced theta-band power in autism spectrum disorder: A resting-state EEG study. International Journal of Psychophysiology. 2019;146:101-6.

168. Neuhaus E, Lowry SJ, Santhosh M, Kresse A, Edwards LA, Keller J, et al. Resting state EEG in youth with ASD: age, sex, and relation to phenotype. Journal of Neurodevelopmental Disorders. 2021;13(1).

169. Keehn B, Vogel-Farley V, Tager-Flusberg H, Nelson CA. Atypical Hemispheric Specialization for Faces in Infants at Risk for Autism Spectrum Disorder. Autism Research. 2015/04/01;8(2).

170. Kolesnik A, Begum Ali J, Gliga T, Guiraud J, Charman T, Johnson MH, et al. Increased cortical reactivity to repeated tones at 8 months in infants with later ASD. Translational Psychiatry 2019 9:1. 2019-01-30;9(1).

171. Leno CV, Pickles A, Noordt VS, Huberty S, Desjardins J, Webb JS, Elsabbagh M. 12-Month peak alpha frequency is a correlate but not a longitudinal predictor of non-verbal cognitive abilities in infants at low and high risk for autism spectrum disorder. Developmental Cognitive Neuroscience. 2021;48:100938.

172. Haartsen R, Charman T, Pasco G, Johnson MH, Jones EJH, Haartsen R, et al. Modulation of EEG theta by naturalistic social content is not altered in infants with family history of autism. Scientific Reports 2022 12:1. 2022-12-01;12(1).
